# SARS-CoV-2 and the role of close contact in transmission: a systematic review

**DOI:** 10.12688/f1000research.52439.1

**Published:** 2021-04-09

**Authors:** Igho J. Onakpoya, Carl J. Heneghan, Elizabeth A. Spencer, Jon Brassey, Annette Plüddemann, David H. Evans, John M. Conly, Tom Jefferson

**Affiliations:** 1 Nuffield Department of Primary Care Health Sciences, University of Oxford, Oxford, OX2 6GG, UK; 2 Trip Database Ltd, Newport, NP20 3PS, UK; 3 Department of Medical Microbiology & Immunology,Li Ka Shing Institute of Virology, University of Alberta, Edmonton, AB, T6G 2R3, Canada; 4 University of Calgary and Alberta Health Services,, University of Calgary, Calgary, AB, T2N 4Z6, Canada

**Keywords:** Close contact, transmission, COVID-19, systematic review

## Abstract

**Background:** SARS-CoV-2 transmission has been reported to be associated with close contact with infected individuals. However, the mechanistic pathway for transmission in close contact settings is unclear. Our objective was to identify, appraise and summarise the evidence from studies assessing the role of close contact in SARS-CoV-2 transmission.

**Methods: **This review is part of an Open Evidence Review on Transmission Dynamics of SARS-CoV-2. We conduct ongoing searches using WHO Covid-19 Database, LitCovid, medRxiv, PubMed and Google Scholar; assess study quality based on the QUADAS-2 criteria and report important findings on an ongoing basis.

**Results:** We included 181 studies: 171 primary studies and 10 systematic reviews. The settings for primary studies were predominantly in home/quarantine facilities (31.6%) and acute care hospitals (15.2%). The overall reporting quality of the studies was low to moderate. There was significant heterogeneity in design and methodology. The frequency of attack rates (PCR testing) was 3.5-75%; attack rates were highest in prison and wedding venues, and in households. The frequency of secondary attack rates was 0.3-100% with rates highest in home/quarantine settings. Three studies showed no transmission if index cases had recurrent infection. Viral culture was performed in three studies of which two found viable virus; culture results were negative where index cases had recurrent infections. Ten studies performed genomic sequencing with phylogenetic analysis – the completeness of genomic similarity ranged from 81-100%. Findings from systematic reviews showed that children were significantly less likely to transmit SARS-CoV-2 and household contact was associated with a significantly increased risk of infection.

**Conclusions:** The evidence from published studies demonstrates that SARS-CoV-2 can be transmitted via close contact settings. The risk of transmission is greater in household contacts. There was wide variation in methodology. Standardized guidelines for reporting transmission in close contact settings should be developed to improve the quality reporting.

## Introduction

The SARS-CoV-2 (COVID-19) pandemic is a major public health concern. Based on WHO data, there have been over 120 million confirmed cases and over two and a half million deaths globally as of 20th March 2021 ^1^. Many national governments have implemented prevention and control measures and vaccines are now being approved and administered; the overall global spread of the virus now appears to be slowing. Current evidence from epidemiologic and virologic studies suggest SARS-CoV-2 is primarily transmitted via respiratory droplets and direct and indirect contact ^2, 3^. However, controversy still exists about how the virus is transmitted and the relative frequency of the modes of transmission and if these modes may be altered in specific settings ^4, 5^.

Although close contact is thought to be associated with transmission of SARS-CoV-2, there is uncertainty about the thresholds of proximity for “close contact” and the factors that may influence the transmission in a “close contact”. Furthermore, there is lack of clarity about how research should be conducted in the setting of transmission with close contact which may include transmission via any one of or the combination of respiratory droplets, direct contact, or indirect contact.

Several studies investigating the role of close contact in SARS-CoV-2 transmission have been published but the pathways and thresholds for transmission are not well established. The objective of this review was to identify, appraise and summarize the evidence from primary studies and systematic reviews investigating the role of close contact in the transmission of SARS-CoV-2. Terminology for this article can be found in Box 1.

**Box 1. Terminology**

**Close contact:** Someone who was within 6 feet of an infected person for a cumulative total of 15 minutes or more over a 24-hour period starting from 2 days before illness onset (or, for asymptomatic patients, 2 days prior to test specimen collection) until the time the patient is isolated ^1^; The World Health Organization (WHO) additionally includes direct physical contact with a probable or confirmed case, direct care for a patient with probable or confirmed COVID-19 disease without using proper PPE, and other situations as indicated by local risk assessments.

**Attack rate:** The proportion of those who become ill after a specified exposure ^2^.

**Secondary attack rate:** The probability that infection occurs among susceptible persons within a reasonable incubation period following known contact with an infectious person or an infectious source ^3^.

**Cycle threshold:** The number of cycles required for the fluorescent signal to cross the threshold. Ct levels are inversely proportional to the amount of target nucleic acid in the sample ^4^.

^1^ https://www.cdc.gov/coronavirus/2019-ncov/global-covid-19/operational-considerations-contact-tracing.html#:~:text=Close contact is defined by, time the patient is isolated

^2^ https://www.who.int/foodsafety/publications/foodborne_disease/Annex_7.pdf

^3^Halloran ME. Secondary Attack Rate. In: Peter A, Theodore C, editors. Encyclopedia of Biostatistics. New York: John Wiley & Sons Ltd; 2005

^4^ https://www.ncbi.nlm.nih.gov/pmc/articles/PMC7521909/

## Methods

We are undertaking an open evidence review examining the factors and circumstances that impact on the transmission of SARS-CoV-2, based on our published protocol last updated on the 1 December 2020 (Version 3: 1 December 2020, *Extended data:* Appendix 1 ^6^). This review aims to identify, appraise, and summarize the evidence (from peer-reviewed studies or studies awaiting peer review) examining the role of close contact in the transmission of SARS-CoV-2 and the factors that influence transmissibility. We are conducting an ongoing search in WHO Covid-19 Database, LitCovid, medRxiv, and Google Scholar for SARS-CoV-2 for keywords and associated synonyms. For this review, we also conducted searches on PubMed. The searches for this update were conducted up to 20th December 2020 ( *Extended data:* Appendix 2 ^6^). We did not impose any language restrictions.

We included studies of any design that investigated transmission associated with close contact but excluded predictive or modelling studies. We reviewed the results for relevance and for articles that appeared particularly relevant, we undertook forward citation matching to identify relevant results. We assessed the risk of bias of included primary studies using five domains from the QUADAS-2 criteria ^7^; we adapted this tool because the included studies were not primarily designed as diagnostic accuracy studies. We did not perform formal assessments of the quality of included systematic reviews but summarized their findings, including quality of their included studies as reported by the authors. We extracted the following information from included studies: study design characteristics including the definition used of “close contact”, population, main methods, and associated outcomes including the number of swab samples taken with frequency and timing of samples, and cycle thresholds and samples concentrations. We also extracted information on viral cultures including the methods used. One reviewer (IJO) assessed the risk of bias from primary studies, and these were independently verified by a second reviewer (EAS). One reviewer (IJO) extracted data from the included primary studies, and these were independently checked by a second reviewer (CJH). One reviewer (CJH) extracted data from the included systematic reviews, and these were independently checked by a second reviewer (IJO). Disagreements in the data extraction or bias assessments were resolved by consensus. We presented the results in tabular format, and bar charts used to present the frequency of positive tests. We reported results of specific subgroups of studies where relevant. Because of substantial heterogeneity across the included studies, we considered meta-analyses inappropriate.

## Results

**Figure 1.**
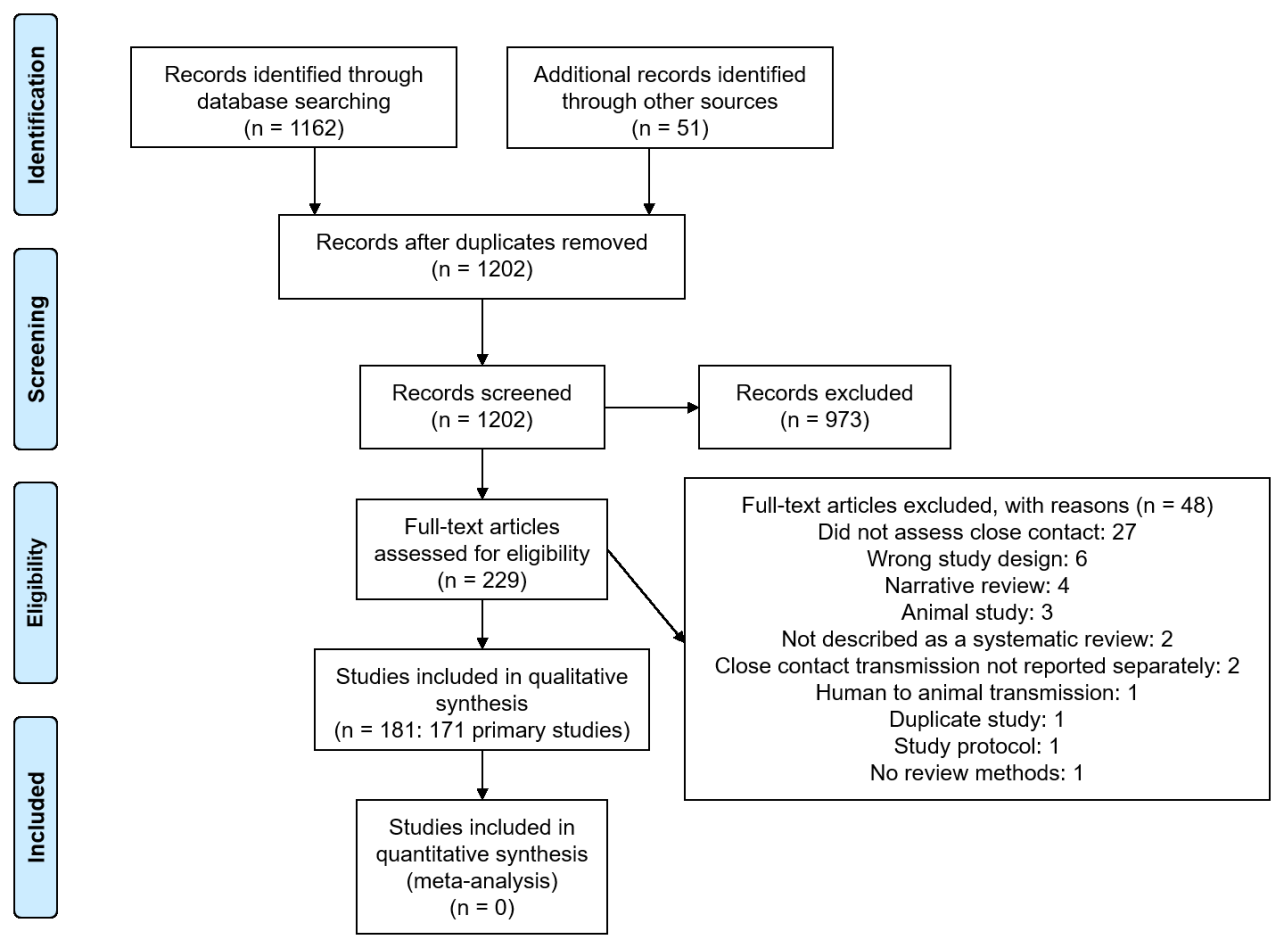
Flow diagram showing the process for inclusion of studies assessing close contact transmission in SARS-CoV-2.

We identified 1202 non-duplicate citations of which 229 were considered eligible ( Figure 1). We excluded 48 full-text studies for various reasons (see *Extended data:* Appendix 3 ^6^ for the list of excluded studies and reasons for exclusion). Finally, we included 181 studies: 171 primary studies and 10 systematic reviews (see *Extended data:* Appendix 4 for references to included studies). The main characteristics of the included primary studies and systematic reviews are shown in Table 1 and Table 2, respectively. 

**Table 1.** Close contact study characteristics.

Study ID	Country	Study Design/Setting	Type of transmission	Population/environment	Test method	Timing of sample collection	Viral culture	Cycle threshold	Other information
Abdulrahman 2020	Bahrain	Observational comparative Country-wide 09/2020	Community	Before and after study of subjects attending 2 religious events	PCR	Not reported	No	>40 was considered negative	A 10-day period before the event was compared to a 10-day period beginning 10 days after the event. All symptomatic individuals and close contacts to a confirmed case were tested. Positive and negative controls were included for quality control purposes.
Adamik 2020	Poland	Observational Home	Household	9756 index cases; 3553 secondary cases	Not reported	Not reported	No	No	Only cases for which clear epidemiological links were registered as household transmission together with their source cases were included. Cases in social care units and households of minimum 15 inhabitants were removed from the analysis, as an initial analysis revealed that those were not representative for the overall population, due to over- represented comorbidities and severe cases.
Agergaard 2020	Denmark	Home quarantine with 1 asymptomatic index case 11/03/2020 to 01/04/2020	Household	Family cluster of 5: Index case arranged a self-imposed 2-week home quarantine along with family of four	PCR Serology	Not reported for PCR	No	Not specified for PCR	Index case recently returned from skiing trip in Austria iFlash SARS-CoV-2 N/S IgM/IgG cut-off: ≥12 AU/ml = positive. DiaSorin SARS-CoV-2 S1/S2 IgG cut-off: ≥15 AU/ml = positive
Angulo-Bazán 2020	Peru	Observational retrospective Household 23/04/2020 to 02/05/2020	Household	52 households in Metropolitan Lima with only one member with COVID-19 Contacts cohabited in same home with index case	RT-PCR (index) Serology	Not reported	No	Not specified	Evaluation was conducted 13.6 ± 3.7 days after the diagnostic test
Armann 2020	Germany	Observational - cross-sectional Schools, homes May to October 2020	Local Household	1538 students and 507 teachers were initially enrolled, and 1334 students and 445 teachers completed both study visits.	Serology	Week 0 and Week 16	No	N/A	an index (S/C) of < 1.4 was considered negative whereas one >/= 1.4 was considered positive) and an ELISA detecting IgG against the S1 domain of the SARS-CoV-2 spike protein (Euroimmun® Anti-SARS CoV-2 ELISA) (a ratio < 0.8 was considered negative, 0.8–1.1 equivocal, > 1.1 positive)
Arnedo-Pena 2020	Spain	Retrospective cohort Homes February-May 2020	Household	347 index cases: 745 household contacts	RT-PCR	Not reported	No	Not specified	COVID-19 cases of community outbreaks and from institutions as nursing homes were excluded. Secondary attack rate was defined as the proportion of secondary cases from the total of contacts that live in the household of index case.
Baker 2020	USA	Observational Acute-care hospital	Nosocomial	44 HCWs who provided care for a hospitalized patient with COVID-19 without PPE due to delayed diagnosis of COVID-19	RT-PCR	Not reported	No	Not specified	Contact and droplet precautions (including eye protection) were instituted
Baettig 2020	Switzerland	Retrospective case series Military canton March 2020	Local	1 index case; 55 contacts	RT-PCR Serology	PCR: Within 24 hrs of index case for symptomatic subjects Serology: 14 days post-exposure	No	Not reported	Positive cases were defined with two positive PCR testing for SARS-CoV-2 from nasopharyngeal swabs.
Bao 2020	China	Observational Entertainment venue January and February 2020	Community	Potentially exposed workers, customers and their family members potentially exposed to COVID‐19 subject at a swimming pool	RT-PCR	Not reported	No	Not specified	Men and women exhibited different usage behaviour in that male bathers occupied the entire area, but mainly stayed at the lounge hall, while female bathers always went home after a bath. The temperature and humidity were significantly higher than what they would have been in an open air‐conditioning environment.
Basso 2020	Norway	Observational study Hospital	Nosocomial	Quarantined HCWs exposed to COVID-19 patient	PCR Serology	Approximately 2 weeks after viral exposure; 3 weeks for serology	No	N/A S/CO ratio ≥1 is positive for antibody	The HCWs were quarantined for 2 weeks due to participation in aerosol-generating procedures (AGPs) with insufficient personal protective equipment (PPE), or close contact viral exposure (defined as ≤2 m for ≥15 min).
Bays 2020	USA	Observational study Community hospital and university medical centre February and March, 2020	Nosocomial	Two index patients and 421 exposed HCWs	RT-PCR	Not reported	No	Not specified	Exposed staff were identified by analyzing the EMR and conducting active case finding in combination with structured interviews. They wore neither surgical masks nor eye protection, and were risk stratified based on examination of the medical record and subsequent phone interviews as follows: high risk: nose or mouth exposed during intubation or bronchoscopy; moderate: nose or mouth exposed and for over 2 minutes; and low: nose or mouth exposed under 2 minutes. Ct was 25 for 1 index case - day 15
Bi 2020	China	Retrospective cohort Home or quarantine facility January-February 2020	Local Household Community	391 SARS-CoV-2 cases and 1286 close contacts	RT-PCR	RT-PCR	No	Not reporred	Close contacts were identified through contact tracing of a confirmed case and were defined as those who lived in the same apartment, shared a meal, travelled, or socially interacted with an index case 2 days before symptom onset. Casual contacts (eg, other clinic patients) and some close contacts (eg, nurses) who wore a mask during exposure were not included in this group.
Blaisdell 2020	USA	Observational study 4 overnight camps June–August 2020	Community	Multilayered prevention and mitigation strategy 642 children and 380 staff members, aged 7–70 years	RT-PCR	4.1 to 9.1 days after camp arrival	No	Not specified	Hygiene measures: Precamp quarantine, pre- and postarrival testing and symptom screening, cohorting, and physical distancing between cohorts. In addition, camps required use of face coverings, enhanced hygiene measures, enhanced cleaning and disinfecting, maximal outdoor programming, and early and rapid identification of infection and isolation.
Böhmer 2020	Germany	Observational Workplace, home January-February 2020	Local Household	1 index case; 241 contacts	RT-PCR WGS	3–5 days post-exposure	No	Not reported	
Boscolo-Rizzo 2020	Italy	Cross-sectional Homes March to April 2020	Household	179 primary cases; 296 household contacts	RT-PCR	Unclear	No	Not reported	
Brown 2020	USA	Survey - cross-sectional Classroom February to March, 2020	Local	Students exposed to an index case (teacher)	Serology	2 weeks post-exposre to index case	No	Reciprocal titers of >400 considered positive Reciprocal titers of >100 but <400 considered indeterminate	
Burke 2020	USA	Observational prospective Homes February to March 2020	Household	10 primary cases; 445 close contacts	Not reported	Within 2 weeks of exposure to infected case	No	Not reported	19 (4%) of the 445 contacts were members of a patient’s household, and five of these 19 contacts continued to have household exposure to the patient with confirmed COVID- 19 during the patient’s isolation period; 104 (23%) were community members who spent at least 10 minutes within 6 feet of a patient with confirmed disease; 100 (22%) were community members who were exposed** to a patient in a health care setting; and 222 (50%) were health care personnel
Canova 2020	Switzerland	Observational case series Primary care setting	Nosocomial	1 index case; 21 HCWs who interacted with index case without PPE	RT-PCR	7 days after the initial exposure	No	Not reporred	
Cariani 2020	Italy	Retrospective Hospital March to April 2020	Nosocomial	HCWs in close contact with SARS-CoV-2- positive cases (patients, co-workers, or relatives), or with symptoms of RTI	RT-PCR	Not reported	No	<40 considered positive	
Charlotte 2020	France	Retrospective Indoor choir rehearsal March 2020	Community	Nonventilated room; sitting less close to one another than usual, but at a distance of <1.82m	RT-PCR	Not reported	No	Not reported	
Chaw 2020	Brunei	Observational Various March 2020	Local Community	Primary cases: Presumably infected at religious event in Malaysia Secondary cases: Epidemiologic link to a primary case	RT-PCR	Not reported	No	Not reported	Household, workplace, social, and a local religious gathering. Initial cluster of SARS-CoV-2 cases arose from 19 persons who had attended the Tablighi Jama’at gathering in Malaysia, resulting in 52 locally transmitted cases.
Chen 2020	China	Aircraft 24 January 2020	Aircraft	Close contact to 2 passengers presenting with a fever and URTI symptoms	RT-PCR	Not reported	No	Not reported	The aircraft was equipped with air handling systems.
Chen 2020a	China	Retrospective observational Home or workplace January-March 2020	Local Household	69 recurrent-positive patients; 209 close contacts	RT-PCR	Every 3 days	No	Not specified	
Chen 2020b	China	Prospective cohort Hospital January-February 2020	Nosocomial	5 index patients; 105 HCWs	RT-PCR Serology	From 14 days post-exposure: 1st & 14th day of quarantine	No	<40 considered positive	
Chen 2020c	China	Observational Various January to March 2020	Local Household Community Nosocomial	157 locally reported confirmed cases, 30 asymptomatic infections; 2147 close contacts	Not reported	Unclear	No	Not reported	Family members, relatives, friends/pilgrims, colleagues/ classmates, medical staff, and general personnel judged by the investigator.
Cheng 2020	Taiwan	Observational Homes, hospital January to March 2020	Household Nosocomial	100 confirmed cases of confirmed; 2761 close contacts	RT-PCR	Unclear	No	Not reported	
Chu 2020	USA	Observational Various January 2020	Community	Close contacts for an early confirmed case of COVID-19	RT-PCR Serology	Unclear	No	Antibody titers >400 considered seropositive.	Office, Community, Urgent care clinic identified via contact tracing
Chu 2020a	USA	Retrospective cohort study Household	Household	Household contacts of primary cases defined as children and adolescents with lab-confirmed COVID-19 (n=224)	Not reported	Not reported	No	Not reported	Did not distinguish between confirmed and probable cases among household contacts. A “primary case” is camp attendee with the earliest onset date in the household and a “secondary case” as a household contact with confirmed or probable COVID-19.
Contejean 2020	France	Observational Comparative Tertiary-care university hospital Feb-Mar 2020	Nosocomial	HCW exposed to COVID-19 patients	RT-PCR	Not reported	No	Not reported: result was +ve if 3/5 of gene targets amplified	Hygiene measures: All employees were encouraged to wear a face mask as often as possible in hospital (particularly in the presence of other persons), to wash/disinfect their hands regularly (and after every contact with other persons), to stay at least 2 meters away from others, to cover their mouth and nose with a tissue or sleeve when coughing or sneezing, to put used tissues in the bin immediately and wash hands afterwards, to avoid touching eyes, mouth. Educational messages were released on the internal website and on posters placed in all hospital premises.
COVID-19 National Emergency Response Center 2020	S. Korea	Observational Various January to March 2020	Local Household Nosocomial	30 cases; 2,370 contacts	RT-PCR	Not reported	No	Not reported	Homes, work, hospitals
Danis 2020	France	Observational case series Chalet, school January to February 2020	Local Household	I adult case with 15 contacts in chalet; 1 paediatric case with 172 school contacts	RT-PCR	Within 5 days of diagnosis of cases	No	Not reported	The index case stayed 4 days in the chalet with 10 English tourists and a family of 5 French residents. One pediatric case, with picornavirus and influenza A coinfection, visited 3 different schools while symptomatic.
Dattner 2020	Israel	Observational Home March to June 2020	Household	637 households, average household size of 5.3	RT-PCR Serology	Serology: 4 weeks post PCR testing	No	Not reported	
de Brito 2020	Brazil	Observational descriptive Household April-May 2020	Household	Socially distanced household contacts of index case	RT-PCR Serology	Serology: 4 weeks post-exposure PCR unclear	No	Not reported	Index case: First member of the cluster who had symptoms and who had a known risk of exposure outside the household during the family's stay in the same condominium; secondary case: Contacts with the index case. Asymptomatic patients: Those who had household contact and positive serology but no symptoms. Probable cases corresponded to confirmed case contacts who developed symptoms compatible with COVID despite negative serology and/or negative RT-PCR results.
Deng 2020	China	Observational Home January to February 2020	Household	27 cases; 347 close contacts	Not reported	Not reported	No	Not reported	
Desmet 2020	Belgium	Observational - cross-sectional School November 2019 to March 2020	Local	84 aged between 6 and 30 months attending daycare	RT-PCR	First weeks of the epidemic in Belgium	No	Not reported	
Dimcheff 2020	USA	Survey: cross-sectional Tertiary-care referral facility June 8 to July 8, 2020	Community Nosocomial Household	HCW exposed to COVID-19 patients either in or outside hospital	Serology	8 weeks post-exposure	No	Not reported	Hygiene measures: Daily COVID-19 symptom screening upon building entry, exclusion of visitors from the facility, and institution of telework in remote offices or at home, isolation of confirmed COVID-19 patients, conversion of COVID-19 wards to negative pressure environments, use of PAPRs) or N95 respirators along with PPE by staff.
Dong 2020	China	Observational Homes	Household	135 cases; 259 close contacts	Not reported	Not reported	No	Not reported	
Doung-ngern 2020	Thailand	Retrospective case-control Various March to April 2020	Local	3 large clusters in nightclubs, boxing stadiums, and a state enterprise office	RT-PCR	Not reported	No	Not reported	Hygiene measures: Consistent wearing of masks, handwashing, and social distancing in public.
Draper 2020	Australia	Observational Various March to April 2020	Local Household Nosocomial	28 cases; 445 close contacts	RT-PCR	Within 2 weeks of exposure to infected case	No	Not reported	Cruise ship, homes, aircraft, hospital
Dub 2020	Finland	Retrospective cohort (2) School and Household	Local Household	School and household contacts of 2 index cases who contracted COVID-19 at school	RT-PCR Serology	Serology: >4 weeks post-exposure	No	MNT titre of ≥ 6 considered positive FMIA titre 3·4 U/ml considered positive	
Expert Taskforce 2020	Japan	Observational prospective Cruise ship February 2020	Local	3,711 persons in cruise ship	RT-PCR	Not reported	No	Not reported	Passengers were allowed a 60-minute period on an exterior deck each day, during which they were instructed to wear masks, refrain from touching anything, and maintain a 1-meter distance from others. Monitors observed these periods. After each group came a 30-minute period in which the areas were disinfected. Room cleaning was suspended. Food and clean linens were delivered to cabin doors by crew, and dirty dishes and linens were picked up at cabin doors by crew. Only symptomatic close contacts were tested initially.
Fateh-Moghadam 2020	Italy	Observational Various March to April 2020	Community	2,812 cases; 6,690 community contacts	Not reported	Not reported	No	Not reported	Institutional settings including nursing homes, hospitals, day and residential centers for the disabled and similar structures, and convents
Firestone 2020	USA	Observational retrospective Motorcycle rally August-September 2020	Local	51 primary event-associated cases, and 35 secondary or tertiary cases	RT-PCR WGS Phylogenetic analysis	Unclear	No	Not reported	**Secondary cases**: Laboratory-confirmed infections in persons who did not attend the rally but who received SARS-CoV- 2–positive test results after having contact with a person who had a primary case during their infectious period. **Tertiary ** **cases** were laboratory-confirmed cases in persons who had contact with a person who had a secondary case during their infectious period. SARS-CoV-2 RNA-positive clinical specimens were obtained from clinical laboratories, and
Fontanet 2020	France	Retrospective cohort study School March to April 2020	Local	661 participants: pupils, their parents and siblings, as well as teachers and non- teaching staff of a high-school	Serology	10 weeks	No	N/A	
Fontanet 2020a	France	Retrospective cohort study Schools April 2020	Local	510 participants: pupils, their parents and siblings, as well as teachers and non- teaching staff of a high-school	Serology	10 weeks	No	N/A	6 primary schools
Gan 2020	China	Observational retrospective survey Various January-February 2020	Local Household Community	1 052 cases in 366 epidemic clusters	Not reported	Not reported	No	Not reported	Family living together, gathering dinner, collective work, ride- thy-car, other aggregation exposure,
Ghinai 2020	USA	Observational 2 Social gatherings January-March 2020	Community	16 cases (7 confirmed and 9 probable) (1 index case)	RT-PCR	Not reported	No	Not reported	A birthday party, funeral, and church attendance.
Gong 2020	China	Observational Various January-February 2020	Household Community	3 clusters: 5 index cases; 9 close contacts	RT-PCR	Not reported	No	Not reported	Travelling and dining, or were living together
Gong 2020	China	Observational Karaoke room January 2020	Local	14 people exposed to 2 index cases in a karaoke room	RT-PCR Serology	PCR: Within 72 hrs post-exposure Serology: 6 weeks post-exposure	No	Not reported	
Hamner 2020	USA	Observational Choir practice March 2020	Local	1 index case; 60 close contacts	RT-PCR	Within 2 weeks of index case	No	Not reported	
Han 2020	S. Korea	Observational Spa facility Mar-April 2020	Community	Contacts for 10 index cases from Spa facility	RT-PCR	Not reported	No	Not reported	
Heavey 2020	Ireland	Observational School March 2020	Local	6 index cases; 1155 contacts	Not reported	Not reported	No	No	Three paediatric cases and three adult cases of COVID-19 with a history of school attendance were identified. Exposed at school in the classroom, during sports lessons, music lessons and during choir practice for a religious ceremony, which involved a number of schools mixing in a church environment.
Helsingen 2020	Norway	RCT Training facilities May-June 2020	Local	Members of the participating training facilities age 18 years or older who were not at increased risk for severe Covid-19	RT-PCR Serology	Serology: 4 weeks after start of study	No	Not reported	Hygiene measures: Avoidance of body contact; 1 metre distance between individuals at all times; 2 metre distance for high intensity activities; provision of disinfectants at all work stations; cleaning requirements of all equipment after use by participant; regular cleaning of facilities and access control by facility employees to ensure distance measures and avoid overcrowding. Changing rooms were open, but showers and saunas remained closed. All participants were mailed a home-test kit including two swabs and a tube with virus transport medium for SARS-CoV-2 RNA
Hendrix 2020	USA	Observational Hair salon May 2020	Local	Contacts for 2 stylists who tested positive for COVID-19	PCR	Not reported	No	Not reported	Hygiene measures: During all interactions with clients at salon A, stylist A wore a double-layered cotton face covering, and stylist B wore a double-layered cotton face covering or a surgical mask.
Hirschman 2020	USA	Observational study Home and social gatherings June 2020	Household Community	2 index cases; 58 primary and secondary contacts	RT-PCR	Unclear	No	Not reported	
Hobbs 2020	USA	Case-control study University Medical Centre September-November 2020	Local Household Community	397 children and adolescents: Cases 154; controls 243	RT-PCR	Not reported	No	Not reported	
Hoehl 2020	Germany	Observational Daycare Centre 12 weeks (June-Sept 2020)	Local Community	Attendees and staff from 50 daycare centres	RT-PCR	Not reported	No	Not reported	Hygiene measures: Barring children and staff with symptoms of COVID-19, other than runny nose, from entering the facilities, as well as denying access to individuals with known exposure to SARS-CoV-2. Access to the facilities was also denied to children if a household member was symptomatic, or was in quarantine due to contact with SARS-CoV-2. Wearing of masks was not mandatory for children or nor staff. The access of caregivers to the facilities was limited.
Hong 2020	China	Observational prospective Home January-April 2020	Household	9 patients with recurrent infection; 13 close contacts	RT-PCR Serology NGS	After re-admission of index patients.	No	Not reported	
Hu 2020	China	Observational retrospective Various January to April 2020	Household Community	1178 cases; 15,648 contacts	Not reported	Not reported	No	Not reported	Homes, social events, travel, other settings
Hua 2020	China	Observational retrospective Home January to April 2020	Household	Children and adult contacts from the 314 families	RT-PCR	Not reported	No	Not reported	
Huang 2020	China	Prospective contact-tracing study Restaurant, home January 2020	Household Community	1 indes case; 22 close contacts	RT-PCR	Within 3 days of index cases	No	Not reported	Close contacts quarantined at home or hospital
Huang 2020a	Taiwan	Retrospective case series Various January-April 2020	Local Household Community Nosocomial	15 primary cases: 3795 close contacts	RT-PCR	Not reported	No	Not reported	Aircraft, home, classroom, workplace, hospital
Islam 2020	Bangladesh	Observational Various March to June 2020	Local Household Community Nosocomial	181 cases; 391 close contacts	Not reported	Not reported	No	Not reported	Household, health care facility, funeral ceremony, public transportation, family members, and others
Jia 2020	China	Observational Home January to February 2020	Household	11 clusters (n=583)	RT-PCR	Not reported	No	<37 considered positive	A **close contact** was defined as a person who did not take effective protection against a suspected or confirmed case 2 d before the onset of symptoms or an asymptomatic infected person 2 d before sampling. Ct-value of 40 or more was defined as negative.
Jiang 2020	China	Observational Home January to February 2020	Household Community	8 index cases, 300 contacts	rRT-PCR WGS Phylogenetic analysis	Every 24 hours for 2 weeks	No	<37 considered positive	Ct value ≥40 was considered negative. The maximum likelihood phylogenetic tree of the complete genomes was conducted by using RAxML software with 1000 bootstrap replicates, employing the general time-reversible nucleotide substitution mode
Jing 2020	China	Retrospective cohort study Homes January-February 2020	Household	195 unrelated close contact groups (215 primary cases, 134 secondary or tertiary cases, and 1964 uninfected close contacts)	RT-PCR	Days 1 and 14 of quarantine	No	Not reported	
Jing 2020a	China	Observational study Homes, public places February 2020	Household Community	68 clusters involving 217 cases	RT-PCR	Not reported	No	Not reported	
Jones 2020	UK France	Observational Super League Rugby August to October 2020	Local	136: 8 index cases: 28 identified close contacts and 100 other players	RT-PCR	Within 14 days of match day	No	Not specified: Ct for index cases 17.8 to 27	**Close contacts** were defined by analysis of video footage for player interactions and microtechnology (GPS) data for proximity analysis. All participants were within a ≤7-day RT-PCR screening cycle
Kang 2020	S. Korea	Observational Night clubs April-May 2020	Local	96 primary cases and 150 secondary cases; 5,517 visitors	Not reported	Not reported	No	Not reported	
Kant 2020	India	Retrospective (contact tracing) Regional Medical Research Centre May 2020	Local Community Nosocomial	1 index case diagnosed post-mortem; number of exposures unclear	RT-PCR	Unclear	No	Not reported	Contacts traced: People from the market where the index case had his shop, his treating physicians, people who attended his funeral, family members and friends
Kawasuji 2020	Japan	Case-control study University Hospital April-May 2020	Nosocomial	28 index cases: 105 close contacts	RT-PCR	Unclear	No	Not reported	Index patients and those with secondary transmission were estimated based on serial intervals in the family clusters.
Khanh 2020	Vietnam	Retrospective Aircraft March 2020	Community	1 index case: 217 close contacts	PCR	4 days after positive test result of index case	No	Not reported	Successfully traced passengers and crew members were interviewed by use of a standard questionnaire, tested for SARS-CoV-2
Kim 2020	S. Korea	Retrospective observational Home setting January-April 2020	Household	107 paediatric index cases: 248 household members of which 207 were exposed	RT-PCR	Within 2 days of COVID-19 diagnosis of the index case	No	Ct value of ≤35 is positive and >40 is negative	Guardian wore a KF94 (N95 equivalent) mask, gloves, full body suit (or waterproof long-sleeve gowns) and goggles.
Kim 2020a	S. Korea	Case series Various January-February 2020	Household Community	1 index case; 4 close contacts	RT-PCR	4 days post-exposure	No	N/A	2 household contacts, 1 church contact, 1 restaurant
Kim 2020b	S. Korea	Retrospective observational University hospital February 2020	Nosocomial	4 confirmed cases: 290 contacts	RT-PCR	Within 8 days of index case diagnosis	No	Ct <35 was considered positive	Medical staff in the triage room used level-D PPE and everyone in the hospital was encouraged to wear masks and follow hand hygiene practices. Contact with confirmed COVID-19 cases was frequent among inpatients and medical support personnel.
Kumar 2020	India	Observational Community March-May 2020	Community	144 source cases:	RT-PCR	Unclear	No	Not reported	Persons with symptoms of ILI and SARI as well as known high- risk contacts of a confirmed COVID-19 patient were included.
Kuwelker 2020	Norway	Prospective case-ascertained study Homes Feb-April 2020	Household	112 index cases; 179 household members	Serology	6–8 weeks after symptom onset in the index case.	No	N/A	Single-person households were excluded from the analysis. Serum samples from index cases and household members were collected 6–8 weeks after symptom onset in the index case.
Kwok 2020	Hong Kong	Retrospective observational Quarantine or isolation February 2020	Local Household	53 cases; 206 close contacts	Not reported	Not reported	No	Not reported	A **secondary case** referred to the first generation of infection induced by an index case following contact with this case
Ladhani 2020	UK	Prospective Care homes April 2020	Nosocomial	6 London care homes reporting a suspected outbreak (2 or more cases); 254 staff members	RT-PCR	Not reported	No	Not reported	254 of 474 (54%) staff members provided a nasal self-swab; 12 were symptomatic at the time of swabbing
Ladhani 2020a	UK	Prospective Care homes April 2020	Nosocomial	6 London care homes reporting a suspected outbreak (2 or more cases); 254 staff members; 264 residents	RT-PCR	Not reported	Yes	Unclear: Ct values <35 were cultured	254 of 474 (54%) staff members provided a nasal self-swab; 12 were symptomatic at the time of swabbing
Laws 2020	USA	Prospective cohort Home setting March-May 2020	Household	1 pediatric index case: 188 household contacts	RT-PCR	Study enrollment (day 0); study close-out (day 14)	No	Not reported	Index case: household member with earliest symptom onset (and positive SARS-CoV-2 RT-PCR test result). Community prevalence in the 2 metropolitan areas was low during this time, and both were under stay-at-home orders. All enrolled index case patients and household contacts were followed prospectively for 14 days. Five households were selected for intensive swabbing requiring collection of respiratory specimens from all household members during four interim visits regardless of symptom presence.
Laxminarayan 2020	India	Observational Various April to August 2020	Local Household Community	3,084,885 known exposed contacts	Not reported	Not reported	No	Not reported	Individual-level epidemiological data on cases and contacts, as well as laboratory test results, were available from 575,071 tested contacts of 84,965 confirmed cases.
Lee 2020	S. Korea	Observational Hospital February-June 2020	Household	12 paediatric cases; 12 guardians as close contact. All guardians used PPE	Not reported	Not reported	No	Not reported	
Lee 2020a	S. Korea	Observational Homes February to March 2020	Household	23 close contacts	PCR	Unclear	No	Not reported	
Lewis 2020	USA	Observational Homes March to April 2020	Household	58 households (Utah, n = 34; Wisconsin n = 24), 58 primary patients and 188 household contacts	RT-PCR Serology	Not reported	No	Not reported	
Li 2020	China	Observational Home setting Feb 2020	Household	Family cluster of 1 index case: 5 household contacts	RT-PCR	One day after index case tested positive	No	Not reported	Unknown when index case started shedding virus
Li 2020a	China	Observational case series Home, hospital January-February 2020	Household Nosocomial	2-family cluster of 1 index case: 7 close contacts	Not reported	Not reported	No	Not reported	
Li 2020b	China	Retrospective observational Home January-February 2020	Household	3-family cluster of 3 index cases: 14 close contacts	RT-PCR	Every 2–3 days until hospital discharge.	No	<38 considered positive	
Li 2020c	China	Retrospective observational Home January-March 2020	Household	30 cases from 35 cluster-onset families (COFs) and 41 cases from 16 solitary-onset families (SOFs)	Not reported	Not reported	No	Not reported	
Li 2020d	China	Observational Household February to March 2020	Household	105 index patients; 392 household contacts	RT-PCR	Within 2 weeks of exposure to infected case	No	Not reported	
Liu 2020	China	Retrospective observational Home setting Feb 2020	Household	Family cluster of 1 index case: 7 household contacts	RT-PCR	Immediately after index case tested positive	No	If both the nCovORF1ab and nCoV-NP showed positive results, COVID-19 infection was considered	Unclear whether the index case was actually first case
Liu 2020a	China	Retrospective case series Hospital January 2020	Nosocomial	30 HCWs with direct contact with patients	RT-PCR	Not reported	No	<40 considered positive	30 cases have a history of direct contact with patients with neo-coronary pneumonia (within 1 m), 1 to 28 contacts, an average of 12 (7,16) contact times, contact time of 0.5 to 3.5 h, the average cumulative contact time of 2 (1.5, 2.7)h.
Liu 2020b	China	Retrospective cohort study Various January-March 2020	Household Community Nosocomial	1158 index cases: 11,580 contacts	RT-PCR	Every several days	No	Not reported	Homes, social venues, various types of transportations
Liu 2020c	China	Prospective observational	Unclear	147 asymptomatic carriers: 1150 close contacts	RT-PCR	Not reported	No	Not reported	RT-PCR for asymptomatic carriers - testing method not described for close contacts
López 2020	USA	Retrospective contact tracing School setting April-July 2020	Local Household	12 index pediatric cases: 101 facility contacts; 184 overall contacts	RT-PCR	Not reported	No	Not reported	Index case: first confirmed case identified in a person at the child care facility Primary case: Earliest confirmed case linked to the outbreak. Overall attack rates include facility-associated cases, nonfacility contact cases iand all facility staff members and attendees and nonfacility contacts
Lopez Bernal 2020	UK	Observational Homes January to March 2020	Household Community	233 households with two or more people; 472 contacts.	PCR	Unclear	No	Not reported	Healthcare workers, returning travellers and airplane exposures were excluded.
Lucey 2020	Ireland	Observational Hospital March-May 2020	Nosocomial	5 HCWs in cluster 1; 2 HCWs in cluster 3; HCW in cluster 2 not specified; 52 patients infected with SARS-CoV-2;	RT-PCR WGS Phylogenetic analysis	Not reported	No	Not reported	SARS-CoV-2 RNA was extracted from nasopharyngeal swabs obtained from COVID-19 cases and their corresponding HCWs were sequenced to completion. HA COVID-19 was classified into two groups according to the length of admission: >7 days and >14 days. The majority of patients required assistance with mobility (65%) and selfcare (77%)
Luo 2020	China	Observational retrospective Public transport January 2020	Community	1 index case; 243 close contacts	RT-PCR	Within 2 weeks of exposure to index case	No	Not reported	The tour coach was with 49 seats was fully occupied with all windows closed and the ventilation system on during the 2.5-hour trip.
Luo 2020a	China	Prospective cohort study Various January to March 2020	Household Community Nosocomial	391 index cases; 3410 close contacts	RT-PCR Serology	Every 24 hours.	No	Not reported	Homes, public transport; healthcare settings, entertainment venues, workplace, multiple settings
Lyngse 2020	Denmark	Retrospective Homes February to July 2020	Household	990 primary cases; 2226 household contacts	Not reported	Within 14 days of exposure to primary case	No	Not reported	Secondary cases: those who had a positive test within 14 days of the primary case being tested positive. 3 phases of epidemic examined. Assumed that the secondary household members were infected by the household primary case, although some of these secondary cases could represent co-primary cases. A longer cutoff time period could result in misclassification of cases among household members with somewhere else being the source of secondary infections.
Ma 2020	China	Observational Medical isolation	Unclear	1665 close contacts	RT-PCR	Not reported	No	Not reported	
Macartney 2020	Australia	Prospective cohort study Educational settings April to May 2020	Local	27 primary cases; 633 contacts	RT-PCR, serology, or both	PCR: 5–10 days after last case contact if not previously collected Serology: day 21 following last case contact.	No	Not reported	**Index case:** The first identified laboratory-confirmed case who attended the facility while infectious. A school or ECEC setting primary case was defined as the initial infectious case or cases in that setting, and might or might not have been the index case. **Primary case:** Initial infectious case or cases in that setting, and might or might not have been the index case **Secondary case:** Close contact with SARS-CoV-2 infection (detected through nucleic acid testing or serological testing, or both), which was considered likely to have occurred via transmission in that educational setting.
Malheiro 2020	Portugal	Retrospective cohort study Homes March to April 2020	Household	Intervention group (n=98), Control (n=453)	Not reported	Not reported	No	Not reported	The intervention group comprised all COVID-19 confirmed cases that were either identified as close contacts of an index caseor returned from affected areas and placed under mandatory quarantine, with daily follow-up until laboratory confirmation of SARS-CoV-2 infection. The control group included all COVID-19 confirmed cases that were not subject to contact tracing nor to quarantine measures preceding the diagnosis.
Maltezou 2020	Greece	Retrospective observational Home setting February to June 2020	Household	203 SARS-CoV-2-infected children; number of index cases and close contacts unclear	RT-PCR	Not reported	No	Ct >38 considered negative	A **family cluster** was defined as the detection of at least 2 cases of SARS-CoV-2 infection within a family. First case was defined as the first COVID-19 case in a family. High, moderate, or low viral load (Ct <25, 25–30 or >30, respectively)
Maltezou 2020a	Greece	Retrospective observational Home setting February to May 2020	Household	23 family clusters of COVID-19; 109 household members	RT-PCR	Not reported	No	<25, 25–30 or >30	A **family cluster** was defined as the detection of at least 2 cases of SARS-CoV-2 infection within a family. Index case was defined as the first laboratory-diagnosed case in the family.
Mao 2020	China	Cross-sectional study Home, family gatherings January-March 2020	Household Local	67 clusters with 226 cases confirmed cases	RT-PCR	Not reported	No	Not reported	
Martinez-Fierro 2020	Mexico	Cross-sectional June-July 2020	Unclear	19 asymptomatic index cases; 81 contacts	RT-PCR Serology	Not reported	No	Not reported	
Mponponsuo 2020	Canada	Observational Hospital March-April 2020	Nosocomial	5 HCWs were index cases; 39 HCWs (16 underwent testing) and 33 patients were exposed (22 underwent testing)	RT-PCR	Not reported	No	Not reported	All 5 HCWs had E gene cycle threshold (Ct) values between 10.9 and 30.2. Those exposed to the index HCWs were followed for 30 days
Ng 2020	Singapore	Retrospective cohort study Various January-April 2020	Household Local Community	1114 PCR-confirmed COVID-19 index cases in the community in Singapore. 13 026 close contacts (1863 household, 2319 work, and 3588 social)	RT-PCR Serology	If contacts reported symptoms	No	Not reported	**Lower risk contacts:** Other contacts who were with the index case for 10–30 min within 2 m Contacts who reported symptoms were admitted to the hospital for COVID-19 testing by PCR.
Ning 2020	China	Observational study Various January-February 2020	Household Local Community	Local cases: 3,435 close contacts Imported cases: 3,666 close contacts	Not reported	Not reported	No	Not reported	Imported cases, farmers' markets, malls and wildlife exposure
Njuguna 2020	USA	Observational Prison May 2020	Local	98 incarcerated and detained persons	RT-PCR	Not reported	No	Not reported	Unclear how many index or close contacts
Ogawa 2020	Japan	Observational Hospital	Nosocomial	1 index patient; 15 HCWs were contact	RT-PCR Serology	RT-PCR: 10th day after exposure Serology: Before isolation	No	Not specified	Viral culture performed for only the index patient
Paireau 2020	France	Retrospective observational Various January to March 2020	Household Local Nosocomial	735 index cases; 6,082 contacts	RT-PCR	Not reported	No	Not reported	Family, home, work, hospital. Index case: A case whose detection initiated an investigation of its contacts through contact tracing Only contacts who developed symptoms compatible with COVID-19 were tested for SARS-CoV-2
Park 2020	S. Korea	Retrospective observational Various February 2020	Local Household Community	2 index cases; 328 contacts	RT-PCR	24 hrs for 37 first contacts; others within 2 weeks	No	<40 considered positive	Aircraft, home, restaurant, clinic, pharmacy. Contact tracing of COVID-19 cases was conducted from 1 day before symptom onset or 1 day before the case was sampled.
Park 2020a	S. Korea	Observational study Homes January to March 2020	Household Non-household	5,706 COVID-19 index patients; 59,073 contacts	Not reported	Not reported	No	Not reported	
Park 2020b	S. Korea	Observational study Workplace, home March 2020	Local Household	216 employees, 225 household contacts	RT-PCR	Within 2 weeks of report of infected case	No	Not reported	Employees do not generally go between floors, and they do not have an in-house restaurant for meals. Sent a total of 16,628 text messages to persons who stayed >5 minutes near the building X; we tracked these persons by using cell phone location data.
Passarelli 2020	Brazil	Observational Hospital August 2020	Nosocomial	6 index cases; 6 close contacts	RT-PCR	Not reported	No	<40 considered positive	All index cases were asymptomatic hospital visitors
Patel 2020	UK	Retrospective observational Hospital, community March to April 2020	Household	107 cases; 195 household contacts	RT-PCR	Not tested	No	Not reported	
Pavli 2020	Greece	Observational contact tracing Aircraft February to March 2020	Aircraft	6 index cases; 891 contacts	RT-PCR	Not reported	No	Not reported	A COVID-19 case was defined at that time as a case with signs and symptoms compatible with COVID-19 in a patient with laboratory-confirmed SARS-CoV-2 infection, recent travel history to a country with evidence of local transmission of SARS-CoV-2 or close contact with a laboratory-confirmed case
Phiriyasart 2020	Thailand	Observational Homes April 2020	Household	471 household contacts	RT-PCR	Within 5 days of exposure	No	Not reported	
Poletti 2020	Italy	Observational February-April 2020	Unclear	5,484 close contacts from clusters	RT-PCR Serology	Not reported	No	Not reported	Only contacts belonging to clusters (i.e. groups of contacts identified by one positive index case) were included. 1,364 (25%) were tested with only RT-PCR, 3,493 (64%) with only serology at least a month after the reporting date of their index case and 627 (11%) were tested both by RT-PCR and serology.
Pung 2020	Singapore	Observational Various February 2020	Local Community	425 close contacts from 3 clusters; index case unclear	PCR WGS Phylogenetic analysis	Not reported	No	Not reported	Company conference, church, tour group. Close contacts under quarantine for 14 days from last exposure to the individual with confirmed COVID-19, either at home or at designated government quarantine facilities.
Pung 2020a	Singapore	Observational Homes Up till March 2020	Household	277 were primary or co‐primary cases: 875 household contacts	Not reported	Not reported	No	Not reported	Household contacts were tested if they showed symptoms of SARS-CoV-2 infection, or if aged 12 years or below
Qian 2020	Hong Kong	Observational retrospective Various January to February 2020	Local Household Community	Unclear	Not reported	Not reported	No	Not reported	Homes, transport, restaurants, shopping and entertainment venues. Four categories of infected individuals were considered based on their relationship: family members, family relatives, socially connected individuals, and socially non‐connected individuals
Ravindran 2020	Indonesia	Retrospective cohort Wedding March 2020	Local	41 guests; no. of index cases unclear	RT-PCR	Not reported	No	Not reported	**Primary case:** Any person who attended the wedding events in Bali Indonesia during 15–21 March 2020 and who tested positive. **Secondary case:** any person who tested positive on SARS- CoV-2 after the 14 day period and who was a close contact of a COVID-19 case from the wedding events.
Razvi 2020	UK	Observational study Hospital May to June 2020	Nosocomial	2,521 HCWs	Serology	Voluntary first-come, first-served basis	No	N/A	
Rosenberg 2020	USA	Observational retrospective Homes March 2020	Household	229 cases; 498 household contacts	RT-PCR	Not reported	No	Not reported	
Roxby 2020	USA	Observational - cross-sectional Nursing home March 2020	Nosocomial	80 residents and 62 staff members; no index case	RT-PCR	Day 1 and 7 days late	No	No	Residents isolated in their rooms; no communal meals or activities, no visitors allowed in the facility, staff member screening and exclusion of symptomatic staff members implemented. Enhanced hygiene practices were put into effect, including cleaning and disinfection of frequently touched surfaces and additional hand hygiene stations in hallways for workers to use. All residents were tested again 7 days later.
Sang 2020	China	Case series Home February 2020	Household	1 index case; 6 family members	Not reported	Within 24 hrs of index case	No	Not reported	Central air conditioner was always running at home
Schumacher 2020	Qatar	Prospective cohort study Football team June to September 2020	Local	1337; no index cases	RT-PCR Serology	RT-PCR: Every 3–5 days Serology: Every 4 weeks	No	≤30 positive	Strict hygiene measures and regular testing. **Two phases**, the quarantine phase (entry until exit) and the training and match phase (after quarantine exit until the first test done during the week after the last match. Ct >30 but <40 reactive. 1337 subjects were tested at least once; however, some players and staff joined their team and were gradually included in (or left) the programme during the study period.
Schwierzeck 2020	Germany	Observational Hospital paediatric dialysis unit	Nosocomial	1 index case; 48 contacts	RT-PCR	24 hrs after index case	No	Not specified	**Outbreak** was defined as two or more COVID-19 infections resulting from a common exposure
Shah 2020	India	Observational Homes March to July 2020	Household	74 primary cases; 386 household contacts	RT-PCR	Not reported	No	Not reported	
Shen 2020	USA	Observational Social gathering January to February 2020	Household Community	1 index case: 539 social and family contacts	RT-PCR	If contact had symptoms	No	Not specified	
Sikkema 2020	Netherlands	Cross-sectional Hospital March 2020	Nosocomial	1796 HCWs; index case not specified	RT-PCR WGS Phylogenetic analysis	N/A	No	<32 considered positive	HCWs across 3 hospitals.
Son 2020	S. Korea	Observational study Homes January to March 2020	Household	108 primary cases; 3223 contacts	RT-PCR	Unclear	No	Not reported	
Song 2020	China	Observational case series Home January 2020	Household	4 family clusters. 4 index cases: 18 close contacts	RT-PCR	0 to 72 hrs after index case tested positive	No	Not reported	
Speake 2020	Australia	Observational retrospective Aircraft March 2020	Aircraft	241 passengers some of whom had disembarked from 1 of 3 cruise ships that had recently docked in Sydney Harbour. 6 primary cases initially	RT-PCR WGS Phylogenetic analysis	Within 2 weeks of primary cases	Yes	Not specified	**Primary cases** as passengers with SARS-CoV-2 who had been on a cruise ship with a known outbreak in the 14 days before illness onset and whose specimen yielded a virus genomic sequence closely matching that of the ship’s outbreak strain **Secondary cases: P**assengers with PCR-confirmed SARS- CoV-2 infection who had not been on a cruise ship with a known SARS-CoV-2 outbreak within 14 days of illness onset and in whom symptoms developed >48 hours after and within 14 days of the flight; or international passengers who had not been on a cruise ship in the 14 days before illness and whose specimens yielded a WGS lineage not known to be in circulation at their place of origin but that closely matched the lineage of a primary case on the flight.
Stein-Zamir 2020	Israel	Observational - cross-sectional Schools May 2020	Local	1,190 students aged 12–18 years (grades 7–12) and 162 staff members.	PCR	Unclear	No	Not reported	
Sugano 2020	Japan	Observational retrospective Music concerts February 2020	Local	1 index case; 72 exposures	RT-PCR	Not reported	No	Not specified	
Sun 2020	China	Observational Homes	Household	Family clusters	Not reported	Not reported	No	Not reported	
Taylor 2020	USA	Observational Skilled nursing facilities April-June 2020	Nosocomial	259 tested residents, and 341 tested HCP	RT-PCR WGS Phylogenetic analysis	Weekly serial testing (every 7–10 days)	No	Not specified	
Teherani 2020	USA	Observational Homes March to June 2020	Household	32 paediatric cases; 144 household contacts	PCR	Within 2 weeks of exposure to infected case	No	Not reported	Only children who presented with symptoms concerning for COVID-19 infection were included.
Thangaraj 2020	India	Observational Tourist group February 2020	Community	1 index case; 26 close contacts	RT-PCR	Within 24 hrs of index case	No	Not reported	
Torres 2020	Chile	Cross-sectional Community March-May 2020	Community	1009 students and 235 staff	Serology	8–10 weeks after school outbreak	No	N/A	The school was closed on March 13, and the entire community was placed in quarantine
Tshokey 2020	Bhutan	Observational Tourists May 2020	Local Community	27 index cases; 75 high-risk contacts, 1095 primary contacts; 448 secondary contacts	RT-PCR	High-risk contacts: minimum of three times with RT-PCR	No	≤ 40 considered positive	
van der Hoek 2020	Netherlands	Observational Household March to April 2020	Household	231 cases; 709 close contacts. 54 families have 239 participants, 185 of whom are family members.	RT-PCR Serology	Not reported	No	Not reported	
Wang 2020	China	Observational Home January-February 2020	Nosocomial Household	25 HCWs, 43 family members	RT-PCR WGS Phylogenetic analysis	Not reported	No	Not reported	
Wang 2020a	China	Retrospective observational Home February 2020	Household	85 primary cases: 155 household contacts in 78 households	RT-PCR	Not reported	No	<37 considered positive	
Wang 2020b	China	Retrospective cohort study Homes February to March 2020	Household	124 primary cases; 335 close contacts	RT-PCR	Within 2 weeks of symptom onset of the primary case	No	Not reported	
Wee 2020	Singapore	Observational Tertiary Hospital February to May 2020	Nosocomial	28 index cases; 253 staff close-contacts and 45 patient close-contacts	RT-PCR	If patient close-contacts or staff close-contacts developed symptoms	No	Not specified	Infection control bundle was implemented comprising infrastructural enhancements, improved PPE, and social distancing between patients. Patients were advised to wear surgical masks, to remain within their room or cohorted cubicle at all times, and to avoid mingling with each other.
Wendt 2020	Germany	Observational Hospital March 2020	Nosocomial	1 index case physician; 187 contacts with HCWs and 67 contacts with patients - 23 high-risk contacts in total	RT-PCR Serology	5-days post exposure (5 & 10 days post exposre for high-risk contacts	No	<36 or <39 considered positive	All high-risk contacts and the index physician were examined serologically on days 15 or 16 and days 22 or 23 after exposure.
Wolf 2020	Germany	Observational case series Hospital quarantine January-February 2020	Household	Family cluster: 1 index case, 4 close contacts	RT-PCR	5-days after index case tested positive	No	Not reported	The parents were asked to wear masks; wearing masks was not practical for the children.
Wong 2020	Hong Kong	Observational Hospital February 2020	Nosocomial	1 index case in AIIR: 71 staff and 49 patients	RT-PCR	End of 28-day surveillance	No	Not specified	
Wood 2020	UK	Retrospective cohort HCW homes	Household	241,266 adults did not share a household with young children; 41,198, 23,783 and 3,850 shared a household with 1, 2 and 3 or more young children	PCR	Not reported	No	Not reported	Primary exposure was the number of children aged 0 to 11 years in each household.
Wu 2020	China	Retrospective cohort study Various January-February 2020	Household Local Community	144 cases, 2994 close contacts	Not reported	Not reported	No	Not reported	Shared transport, visit, medical care, household, brief contact.
Wu 2020a	China	Prospective observational Homes February to March 2020	Household	35 index cases; 148 household contacts	Not reported	Not reported	No	Not reported	All consecutive patients with probable or confirmed COVID- 19 admitted to the Fifth Affiliated Hospital of Sun Yat-sen University from 17 January to 29 February 2020 were enrolled. All included patients and their household members were interviewed
Xie 2020	China	Cross-sectional Home January-February 2020	Household	2 family clusters with 61 residents (5 cases)	RT-PCR	7 days after primary or index cases diagnosed	No	Not reported	
Xin 2020	China	Prospective cohort study Homes January to March 2020	Household	31 primary cases; 106 household contacts	RT-PCR	Not reported	No	Not reported	
Yang 2020	China	Observational cohort study Home quarantine February-May 2020	Household Local	93 recurrent-positive patients; 96 close contacts and 1,200 candidate contacts	RT-PCR Serology	Within 14 days post-exposure	Yes	≤ 40 considered positive	
Yau 2020	Canada	Retrospective cohort study Hospital dialysis unit April 2020	Nosocomial	2 index cases; 330 contacts (237 patients and 93 staff)	RT-PCR	Not reported	No	Not reported	All symptomatic contacts were referred for testing but asymptomatic household contacts were not routinely tested as per public health protocols at the time.
Ye 2020	China	Observational Religious gathering January-February 2020	Local Community	66 confirmed cases and 15 asymptomatic infections: 1,293 close contacts	RT-PCR	Not reported	No	Not reported	All close contacts were quarantined
Yoon 2020	S. Korea	Observational Childcare Centre February-March 2020	Local	1 index case: 190 persons (154 children and 36 adults) were identified as contacts; 44 were defined as close contacts (37 children and 7 adults)	PCR	8–9 days after the last exposure	No	<37 considered positive	Wearing masks, more frequent hand hygiene, and disinfection of the environment were required before the child index case tested positive.
Yousaf 2020	USA	Survey: cross-sectional Tertiary-care referral facility June 8 to July 8, 2020	Household	198 household contacts; index cases not specified	RT-PCR	Day 1 of study	No	Not reported	
Yu 2020	China	Observational study Homes January to February 2020	Household	560 index cases; 1587 close contacts	Not reported	Within 2 weeks of exposure to primary case	No	Not reported	Exposure environments included workplace, medical centre, etc. Contact methods included eating or living together, sleeping together, living in same house, etc
Yung 2020	Singapore	Observational prospective Homes March to April 2020	Household	137 households, 213 paediatric contacts	Not reported	Unclear	No	Not reported	
Zhang 2020	China	Retrospective Observational Aircraft March-April 2020	Aircraft	4462 passengers screened for COVID-19 based on close contact	RT-PCR	Not reported	No	Not reported	All passengers were quarantined after arrival
Zhang 2020a	China	Retrospective observational Various January-March 2020	Household Local Community	359 cases: 369 close contacts	Not reported	Not reported	No	Not reported	Households, social contact, workplace
Zhang 2020b	China	Observational study Hospital April 2020	Household	3 index cases; 10 close contacts	RT-PCR Serology	Not reported	No	<37 considered positive	Ct value of 40 or more was defined as a negative test.
Zhang 2020c	China	Observational Quarantine January-February 2020	Local Household	Multi-family cluster of 22 cases: 93 close contacts	RT-PCR	Not specified	No	Not reported	All close contacts were quarantined in centralized facilities.
Zhang 2020d	China	Observational Supermarket January-February 2020	Local	1 index case: 8437 contacts	RT-PCR	Not reported	No	Not reported	
Zhuang 2020	China	Observational study Various January to February 2020	Household Community	Cluster outbreaks; 8363 close contacts	Not reported	Not reported	No	Not reported	Family and non-family cases

**Table 2.** Main characteristics of systematic reviews.

Study ID (n=9)	Fulfils systematic review methods	Research question (search date up to)	No. of included studies (No. of participants)	Main results	Key conclusions
Chen 2020	Yes	To estimate seroprevalence by different types of exposures, within each WHO region, we categorized all study participants into five groups: 1) close contacts, 2) high-risk healthcare workers, 3) low-risk healthcare workers, 4) general populations, and 5) poorly-defined populations **(Search from Dec 1, 2019 to Sep 25, 2020).**	230 studies involving 1,445,028 participants were included in our meta-analysis after full-text scrutiny: Close contacts 16 studies 2901 positives out of 9,349 participants	Estimated seroprevalence of all infections, 22.9% [95% CI, 11.1–34.7] compared to relatively low prevalence of SARS- CoV-2 specific antibodies among general populations, 6,5% (5.8–7.2%) see Appendix table 15 (page 152). The overall risk of bias was low.	There were a very limited number of high-quality studies of exposed populations, especially for healthcare workers and close contacts, and studies to address this knowledge gap are needed. Pooled estimates of SARS-CoV-2 seroprevalence based on currently available data demonstrate a higher infection risk among close contacts and healthcare workers lacking PPE,
Chu 2020	Yes	To investigate the effects of physical distance, face masks, and eye protection on virus transmission in health-care and non-health-care (eg, community) settings **(We searched up to March 26, 2020)**	Identified 172 studies; 44 studies included in the meta-analysis which 7 were Covid-19	A strong association was found of proximity of the exposed individual with the risk of infection (unadjusted n=10 736, RR 0·30, 95% CI 0·20 to 0·44; adjusted n=7782, aOR 0·18, 95% CI 0·09 to 0·38; absolute risk [AR] 12·8% with shorter distance vs 2·6% with further distance, risk difference. There were six studies on COVID-19, the association was seen irrespective of causative virus (p value for interaction=0·49). The risk of bias was generally low-to-moderate.	Physical distancing of at least 1 m is strongly associated with protection, but distances of up to 2 m might be more effective.
Fung 2020	Yes	To review and analyze available studies of the household SARs for SARS-CoV-2. Searched PubMed, bioRxiv, and medRxiv on **2** **September 2020** for published and prepublished studies reporting empirical estimates of household SARs for SARS-CoV-2. Considered only English-language records posted on or after 1 January 2019. **Inclusion criteria: ** Reported estimates of the household SAR or the data required to compute the household SAR; (2) comprised data from more than 1 household; and (3) they tested—at a minimum—all symptomatic household contacts by reverse transcription polymerase chain reaction (RT-PCR).	22 papers met the eligibility criteria: 6 papers reported results of prospective studies and 16 reported retrospective studies. The number of household contacts evaluated per study ranged from 11 to 10592.	The 22 studies considered 20 291 household contacts, 3151 (15.5%) of whom tested positive for SARS-CoV-2. Household secondary attack rate estimates ranged from 3.9% in the Northern Territory, Australia to 36.4% in Shandong, China. The overall pooled random-effects estimate of SAR was 17.1% (95% confidence interval [CI], 13.7–21.2%), with significant heterogeneity (p<0.0001). The household secondary attack rates was highest for index cases aged 10–19 years (18.6%; 95% CI, 14.0–24.0%) and lowest for those younger than 9 (5.3%; 95% CI, 1.3–13.7%). 4 of the studies were judges as high quality; 14 as moderate quality; and 4 as low quality. Between-study variation could not be explained by differences in study quality.	Secondary attack rates reported using a single follow-up test may be underestimated, and testing household contacts of COVID-19 cases on multiple occasions may increase the yield for identifying secondary cases. There is a critical need for studies in Africa, South Asia and Latin America to investigate whether there are setting-specific differences that influence the household SAR.
Koh 2020	Yes	The secondary attack rate (SAR) in household and healthcare settings. **Search between Jan 1 and** **July 25, 2020.**	118 studies, 57 were included in the meta-analyses.	Pooled household SAR 18.1% (95% CI: 15.7%, 20.6%) significant heterogeneity (p <0.001). No significant difference in secondary attack rates in terms of the definition of household close contacts, whether based on living in the same household (18.2%; 95% CI: 15.3%, 21.2%) or on relationships such as family and close relatives (17.8%; 95% CI: 13.8%, 21.8%) In three studies, the household secondary attack rates of symptomatic index cases (20.0%; 95% CI: 11.4%, 28.6%) was higher than asymptomatic ones (4.7%; 95% CI: 1.1%, 8.3%) SAR from 14 studies showed close contacts adults were more likely to be infected compared to children (<18), relative risk 1.71 (95% CI: 1.35, 2.17). 43 high-quality studies were included for meta-analysis.	There was variation in the definition of household contacts; most included only those who resided with the index case, some studies expanded this to include others who spent at least a night in the same residence or a specified duration of at least 24 hours of living together, while others included family members or close relatives.
Li 2020	No (quality assesment not performed)	~Carriage and transmission potential of SARS- CoV-2 in children in school and community settings **(Search performed on 21 June 2020** **with entry date** **limits from late 2019)**	33 studies were included for this review. Four new studies on SARS-CoV-2 transmission in school settings were identified.	There is a lack of direct evidence on the dynamics of child transmission, however the evidence to date suggests that children are unlikely to be major transmitters of SARS-CoV-2.	The balance of evidence suggests that children play only a limited role in overall transmission, but it is noted that the relative contribution of children to SARS-CoV-2 transmission may change with reopening of society and schools
Ludvigsson 2020	No (quality assesment not performed)	Are children the main drivers of the COVID‐19 pandemic **(Search to 11 May 2020)**	47 full texts studied in detail.	This review showed that children constituted a small fraction of individuals with COVID‐19	Children are unlikely to be the main drivers of the pandemic. Data on viral loads were scarce, but indicated that children may have lower levels than adults,
Madewell 2020	Yes	What is the household secondary attack rate for severe acute respiratory syndrome coronavirus 2 (SARS-CoV-2)? ( **Searched through Oct 19, 2020)** single database assessed	54 studies with 77,758 participants	Household secondary attack rates was 16.6%; restricted index cases to children (<18 years), lower SAR of 0.5% Secondary attack rates for household and family contacts 3 times higher than for close contacts (4.8%; 95% CI, 3.4%- 6.5%; P < .001); Estimated mean household secondary attack rates from symptomatic index cases (18.0%; 95% CI, 14.2%-22.1%) higher than from asymptomatic or presymptomatic index cases (0.7%; 95% CI, 0%-4.9%; P < .001), there were few studies in the latter group. Infection risk was highest for spouses, followed by nonspouse family members and other relatives, all higher than other contacts. Estimated mean household secondary attack rates to spouses (37.8%; 95% CI, 25.8%-50.5%) higher than to other contacts (17.8%; 95% CI, 11.7%-24.8%). Significant heterogeneity was found among studies of spouses (I2 = 78.6%; P < .001) and other relationships (I2 = 83.5%; P < .001). Contact frequency with index case associated with higher odds of infection, At least 5 contacts during 2 days before the index case was confirmed; at least 4 contacts and 1 to 3 contacts, or frequent contact within 1 meter. Secondary attack rates for households with 1 contact (41.5%; 95% CI, 31.7%-51.7%) higher than households with at least 3 contacts (22.8%; 95% CI, 13.6%-33.5%; P < .001) but not different than households with 2 contacts (38.6%; 95% CI, 17.9%-61.6%). There was significant heterogeneity in secondary attack rates between studies with 1 contact (I2 = 52.9%; P = .049), 2 contacts (I2 = 93.6%; P < .001), or 3 or more contacts (I2 = 91.6%; P < .001). Information was not available on household crowding. A total of 16 of 54 studies (29.6%) were at high risk of bias, 27 (50.0%) were moderate, and 11 (20.4%) were low.	Secondary attack rates were higher in households from symptomatic index cases than asymptomatic index cases, to adult contacts than to child contacts, to spouses than to other family contacts, and in households with 1 contact than households with 3 or more contacts. Our study had several limitations. The most notable is the large amount of unexplained heterogeneity across studies. This is likely attributable to variability in study definitions of index cases and household contacts, frequency and type of testing, sociodemographic factors, household characteristics (eg, density, air ventilation), and local policies (eg, centralized isolation). The findings of this study suggest that households are and will continue to be important venues for transmission, even where community transmission is reduced.
Xu 2020	Yes	Evidence for transmission of COVID-19 by children in schools ( **search in MEDLINE up to ** **14 September 2020.** Further hand-searched reference lists of the retrieved eligible publications to identify additional relevant studies). Included children (defined as ≤18 years old) who were attending school, and their close contacts (family and household members, teachers, school support staff) during the COVID-19 pandemic	11 studies were included: 5 cohort studies and 6 cross- sectional studies.	Overall infection attack rate (IAR) in cohort studies: 0.08%, 95% CI 0.00%-0.86%. IARs for students and school staff were 0.15% (95% CI 0.00%-0.93%) and 0.70% (95% CI = 0.00%- 3.56%) respectively (p<0.01). Six cross-sectional studies reported 639 SARS-CoV-2 positive cases in 6682 study participants tested [overall SARS-CoV-2 positivity rate: 8.00% (95% CI = 2.17%-16.95%). SARS-CoV-2 positivity rate was estimated to be 8.74% (95% CI = 2.34%-18.53%) among students, compared to 13.68% (95% CI = 1.68%-33.89%) among school staff (p<0.01). Overall study quality was judged to be poor with risk of performance and attrition bias	There is limited high-quality evidence to quantify the extent of SARS-CoV-2 transmission in schools or to compare it to community transmission. Emerging evidence suggests lower IAR and SARS-CoV-2 positivity rate in students compared to school staff.
Yanes-Lane 2020	Yes	Proportion of asymptomatic infection among coronavirus disease 2019 (COVID-19) positive persons and their transmission potential. **(Search** ** up to up to 22 June 2020)**	28 moderate/high quality studies included; 43 low quality studies excluded	Asymptomatic COVID-19 infection at time of testing ranged from 20% - 75%; among three studies in contacts it was 8.2% to 50%. Asymptomatic infection in obstetric patients pooled proprtion was 95% (95% CI, 45% to 100%) of which 59% (49% to 68%) remained asymptomatic through follow-up; Among nursing home residents, the proportion of asymtomoatic was 54% (42% to 65%) of which 28% (13% to 50%) remained asymptomatic through follow-up.	The proportion of asymptomatic infection among COVID-19 positive persons appears high and transmission potential seems substantial.
Zhu 2020	Meta-analysis: Quality assessment not performed	Role of children in SARS-CoV-2 in household transmission clusters ( **Search between Dec, ** **2019 & Aug, 2020).**	57 articles with 213 clusters	8 (3.8%) transmission clusters were identified as having a paediatric index case. Asymptomatic index cases were associated with lower secondary attack rates in contacts than symptomatic index cases [RR] 0.17 (95% CI,0.09–0.29). SAR in paediatric household contacts was lower than in adult household contacts (RR, 0.62; 95% CI, 0.42–0.91).	The data suggest that should children become infected at school during this period, they are unlikely to spread SARS-CoV-2 to their co-habiting family members.

### Quality of included studies

**Figure 2.**
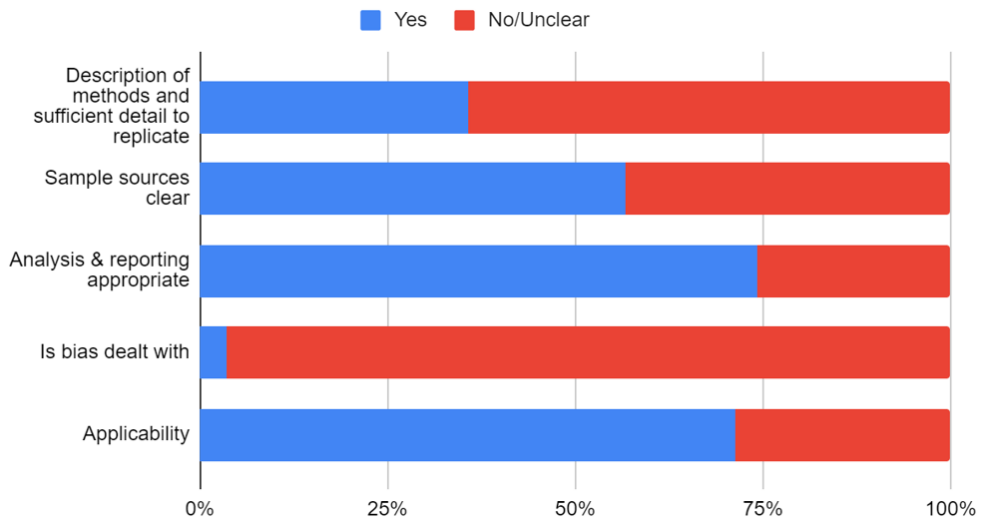
Risk of bias graph in primary studies of close contacts in SARS-CoV-2.

None of the included primary studies reported a published protocol except one (Helsingen 2020). The risk of bias of the included primary studies is shown in Table 5. Only 61 studies (35.7%) adequately reported the methods used, and 97 (56.7%) adequately described the sources of sample collection. Only six studies (3.5%) adequately reported methods used to address biases. The overall quality of the studies was judged as low to moderate (see the risk of bias graph in Figure 2).

### Reviews

We included 10 systematic reviews investigating the role of close contact in SARS-CoV-2 transmission ( Table 2). The studies included in the reviews were primarily observational. In one review (Chen 2020), there was a higher risk of infection in close contacts and healthcare workers without PPE compared to the general population. A second review (Chu 2020) found a significant association between proximity of exposure (distance <1m), absence of barriers (not using face covering or eye protection) and the risk of infection. The authors of three reviews (Li 2020, Ludvigsson 2020, Zhu 2020) concluded that children were unlikely to be the main conduit for transmission of SARS-CoV-2, and results of one review (Koh 2020) showed that adults with close contact exposure were significantly more likely to be infected compared with children (14 studies, RR: 1.71 (95% CI: 1.35, 2.17)). In one review (Xu 2020), the attack rates were significantly less in students compared with staff (p<0.01). One review (Fung 2020) reported household SARs ranging from 3.9% to 36.4%, but also highlighted the lack of SARS-CoV-2 research in Africa, South Asia, and Latin America. One review (Madewell 2020) found that SARs were higher in households from symptomatic index cases than asymptomatic index cases, and one review (Yanes-Lane 2020) concluded that the proportion of asymptomatic infection was high (20–75%). In two reviews (Koh 2020, Yanes-Lane 2020), studies judged to be of low quality were excluded from their meta-analyses. In one review (Chen 2020), the overall quality was reported as low, while 80% of included studies were reported as moderate or high quality in another two (Fung 2020, Madewell 2020). Another review (Chu 2020) reported the overall risk of bias as low-to-moderate, and one (Xu 2020) rated the overall quality as low. Three reviews did not assess study quality (see Table 2).

### Primary studies

We found 171 primary studies ( Table 1). In general, the studies did not report any hypothesis but assessed epidemiological or mechanistic evidence for transmission associated with close contact settings. Ninety-three studies (54.4%) were conducted in Asia, 43 (25.1%) in Europe, 27 (15.8%) in North America, five (2.9%) in South America and three (1.8%) in Australasia. The study settings included home/quarantine facilities (n=54), hospital (n=26), social/religious gatherings (n=13), public transport (n=7) care homes (n=4), and educational settings (n=8). Thirteen studies used two settings (home plus one other setting). In 25 studies (15.2%), the settings were multiple (3 or more different settings). Two studies were conducted in professional sports settings: one Super League Rugby (Jones 2020) and one football team (Schumacher 2020)

All the included studies were observational in design except one RCT (Helsingen 2020): 24 studies were described as cohort, nine were case series and 12 cross-sectional. One study used a before and after study design. The number of close contact participants included ranged from 4 to 8437. Three studies (Chen 2020a, Hong 2020, Yang 2020) examined transmission dynamics in close contacts of index or primary cases with recurrent SARS-CoV-2 infections.

Eighty-two studies (46.8%) reported definitions of close contacts ( Table 3). There was a variation in the definitions across the studies. Seventeen studies (9.9%) defined close contact as exposure to the index or primary case within two metres for at least 15 minutes while four defined it as being within 2m for at least 10 minutes. In 24 studies, there was no specified distance reported - close contact definitions included unprotected exposure, living in the same household or bedroom, sharing a meal, or having repeated and prolonged contact. In five studies of airline passengers, close contact was defined as all passengers on the flight (Chen 2020), seated within two rows of the index case (Draper 2020, Pavli 2020, Speake 2020), or being within 2m for at least 15 minutes (Khanh 2020). Eighty-seven studies (50.9%) did not define close contact and the definition was unclear in four studies. Twenty-nine studies (17%) defined other types of contacts including primary contact, secondary contact, high-risk contact, household contact, social contact, and work contact (see Table 3).

**Table 3.** Definitions and descriptions of close contacts.

Study ID	Definitions of close contacts	Definition of other contacts	Contact duration & proximity
Abdulrahman 2020	Not defined	Not reported	Not reported
Adamik 2020	Not defined	Other cases in each of the infected households were regarded as secondary cases	Not reported
Agergaard 2020	Not defined	Not reported	2 weeks
Angulo-Bazán 2020	Not defined	Not reported	Not reported
Armann 2020	Not defined	Not reported	Not reported
Arnedo-Pena 2020	**Close contacts** living in the same household of the index case and no other sources of transmission apart from the index case could be found.	Closed contacts from work, social events, relatives live in other household were excluded and index cases live alone.	Not reported
Baker 2020	Not defined	Not reported	Median cumulative time spent with the patient 45 mins (10–720 mins)
Baettig 2020	**Close contact:** Less than 2 m for more than 15 min in the last 48 hours before onset symptom of the COVID-19 positive index patient.	Not reported	<2m for >15 mins 48 hours before onset symptom of the COVID-19 positive index patient
Bao 2020	Not defined	Not reported	Average stay duration of 2.5 hr daily before the COVID‐19 outbreak.
Basso 2020	**Close contact:** ≥15 min at ≤2 m, or during AGPs, between HCWs and the non- isolated COVID-19 patient	Not reported	≤2 m for ≥15 min or during AGP
Bays 2020	Not defined	Not reported	Not specified
Bi 2020	**Close contacts** were identified as those who lived in the same apartment, shared a meal, travelled, or socially interacted with an index case 2 days before symptom onset.	Casual contacts (eg, other clinic patients) and some close contacts (eg, nurses) who wore a mask during exposure were not included in this group.	Not specified
Blaisdell 2020	Not defined	Not reported	1 week
Böhmer 2020	High risk if they had cumulative face-to-face contact with a patient with laboratory-confirmed SARS-CoV-2 infection for at least 15 min, had direct contact with secretions or body fluids of a patient with confirmed COVID-19, or, in the case of health-care workers, had worked within 2 m of a patient with confirmed COVID-19 without PPE	All other contacts were classified as low-risk contacts.	Face-to-face for at least 15 minutes, direct contact without PPE
Boscolo-Rizzo 2020	Not defined	Not reported	Not reported
Brown 2020	Not defined	Not reported	Mean in-class time = 50 minutes
Burke 2020	Either at least 10 minutes spent within 6 feet of the patient with confirmed COVID-19 (e.g., in a waiting room) or having spent time in the same airspace (e.g., the same examination room) for 0–2 hours after the confirmed COVID-19 patient.	Not reported	Within 6 feet for at least 10 minutes
Canova 2020	Not defined	Not reported	5 HCWs: >30 minutes 5 HCWs: >15–30 mins 6 HCWs: 5–15 mins 5 HCWs: ≤5 mins
Cariani 2020	Not defined	Not reported	Not reported
Charlotte 2020	Not defined	Not reported	2-hours
Chaw 2020	**Close contact:** Any person living in the same household as a confirmed case- patient or someone who had been within 1 m of a confirmed case-patient in an enclosed space for >15 minutes	Not reported	Within 1m for >15 mins
Chen 2020	**Close contact:** All passengers were regarded as close contacts	Not reported	Flight duration 5 hours approx
Chen 2020a	**Close contacts** are persons who have had close contact with re-positive patients without effective protection with masks, such as living and working together	Not reported	Not specified
Chen 2020b	Not defined	Not reported	Not specified
Chu 2020	**Community contact:** Any close contact (being within 6 feet of the case-patient) for a prolonged time (>10 minutes); being an office co-worker of the case-patient with close contact of any duration; contact with infectious secretions from the case-patient; or sharing a healthcare waiting room or area during the same time and up to 2 hours after the case-patient was present.	Healthcare contact: Face-to-face interaction between healthcare personnel (HCP) and the case-patient without wearing the full PPE that was recommended at the time of the investigation or potential contact with the case-patient’s secretions by HCP without wearing full PPE.	>10 mins to 2 hours
Chen 2020c	Not defined	Not reported	Not reported
Cheng 2020	Close contact was a person who did not wear appropriate PPE while having face-to-face contact with a confirmed case for more than 15 minutes during the investigation period. A contact was listed as a household contact if he or she lived in the same household with the index case. Those listed as family contacts were family members not living in the same household. For health care settings, medical staff, hospital workers, and other patients in the same setting were included; close contact was defined by contacting an index case within 2 m without appropriate PPE and without a minimal requirement of exposure time	Those listed as family contacts were family members not living in the same household.	Within 2 m without PPE, face-to-face contact for >15 minutes
Chu 2020a	Not defined	Not reported	Stayed ≥1 night in the household during case’s infectious period
Contejean 2020	**Close contact:** Distance <2 meters for >10 minutes was defined as close contact	Not reported	<2 metres for >10 minutes
COVID-19 National Emergency Response Center 2020	Close contact (or high risk exposure)” was being within 2 meters of a COVID-19 case	Daily contact (or low risk exposure) was defined as having proximity with a person who was a confirmed COVID-19 case, without having had close contact.	Not reported
Danis 2020	All children and teachers who were in the same class as the symptomatic pediatric case were considered as **high risk contacts** and were isolated at home. **Moderate/high risk:** Person who had prolonged (> 15 min) direct face-to-face contact within 1 m with a confirmed case, shared the same hospital room, lived in the same household or shared any leisure or professional activity in close proximity with a confirmed case, or travelled together with a COVID-19 case in any kind of conveyance, without appropriate individual protection equipment.	**Low risk:** Person who had a close (within 1 m) but short (< 15 min) contact with a confirmed case, or a distant (> 1 m) but prolonged contact in public settings, or any contact in private settings that does not match with the moderate/high risk of exposure criteria. **Negligible risk:** Person who had short (< 15 min) contact with a confirmed case in public settings such as in public transportation, restaurants and shops; healthcare personnel who treated a confirmed case while wearing appropriate PPE without any breach identified.	4 days in chalet
Dattner 2020	Not defined	Not reported	Not reported
de Brito 2020	**Close contact:** Close and prolonged contact in the same room	Not reported	Not specified
Deng 2020	Not defined	Not reported	Not reported
Desmet 2020	Not defined	Not reported	Not reported
Dimcheff 2020	**Close contact:** Within 2 m or 6 feet) with an individual with confirmed COVID-19 for >15 minutes with the example being exposed to a family member at home who has had a positive COVID-19 nasal swab	Not reported	Within 2m for >15 mins
Dong 2020	Not defined	Not reported	Not reported
Doung-ngern 2020	**High-risk** if they were family members or lived in the same household as a COVID-19 patient, if they were within a 1-meter distance of a COVID-19 patient longer than 15 minutes; if they were exposed to coughs, sneezes, or secretions of a COVID-19 patient and were not wearing protective gear, such as a mask; or if they were in the same closed environment within a 1-meter distance of a COVID- 19 patient longer than 15 minutes and were not wearing protective gear	Not reported	<15 min vs >15 min, <1m vs >1m
Draper 2020	Close contact was defined as anyone who had face-to-face contact with a confirmed COVID-19 case for more than 15 minutes cumulatively or continuously (e.g. household setting or healthcare setting without appropriate use of personal protective equipment) or who was in the same room with an infectious case for more than 2 hours (e.g. school room, workplace) while a case was symptomatic or during the 24 hours preceding symptom onset. Aircraft close contacts included passengers seated in the same row as, or in the two rows in front of or behind, an infectious case. If the case was a crew member, the passengers in the area in which the crew member worked were classified as close contacts. Passengers disembarking from cruise ships with high incidence of COVID-19 were also classified as close contacts for surveillance purposes.	Not reported	Not reported
Dub 2020	**Close household contact**, i.e. an individual sharing the main residence of the secondary case	A regular household contact, i.e. an individual who would regularly host or stay in the same residence of a secondary case (step-sibling, divorced parent and new partner). Extended contact, i.e. an individual who would have frequent contact with the secondary case around and after the exposure, for example, grandparents who were involved in caring of the secondary case, according to parents’ reports.	<2 meters for >10 minutes
Expert Taskforce 2020	**Close contact:** Cabinmates of confirmed case-patients	Not reported	Not specified
Fateh-Moghadam 2020	Contact of a COVID-19 case has been considered any person who has had contact with a COVID-19 case within a time frame ranging from 48 hours before the onset of symptoms of the case to 14 days after the onset of symptoms	Not reported	Not reported
Firestone 2020	**Close contact:** Being within 6 feet of a patient with laboratory-confirmed COVID- 19 infection for ≥15 minutes	Not reported	Within 2m for >15 mins
Fontanet 2020	Not defined	Not reported	Not reported
Fontanet 2020a	Not defined	Not reported	Not reported
Gan 2020	Not defined	Not reported	Not reported
Ghinai 2020	Not defined	Not reported	Not reported
Gong 2020	**Close contact:** Anyone who was closely in contact with a suspected, confirmed and asymptomatic case without effective personal protection (classified protection according to the contact situation, including gloves, medical protective masks, protective face screens, isolation clothing, etc.) since onset of symptoms in the suspected case and confirmed case or the day asymptomatic case’s specimens were collected. The close contact included: (i) living, working, or studying in one house or classroom, (ii) diagnosing, treating, or visiting cases in hospital ward, (iii) being within short distance in the same vehicle, (iv) other situations assessed by the field investigators.	Not reported	Not reported
Gu 2020	Not defined	Not reported	5 hrs, no natural ventilation or face masks; distance between each other <0.5 m
Hamner 2020	**Close contact:** Within 6 feet of infected case	Not reported	2.5 hrs within 2 m
Han 2020	**Close contact:** Travel was defined as someone who was in close contact with a confirmed case for over three hours as they traveled to another region aside from Region A. Close contact: meal was defined as someone who was in close contact with a confirmed case for over 30 minutes after having a meal together.	A casual contact was defined as someone who spent several minutes with a confirmed case within the same space without any mask on (or a person was established as a contact by an Epidemic intelligence Officer).	30 mins to 3 hours
Heavey 2020	**Close contact:** Any individual who has had greaterthan 15 minutes face-to-face (<2 meters distance*) contact with a case, in any setting.	Casual contact: Any individual who has shared a closed space with a case for less than two hours.	Up to 2 hours in duration
Helsingen 2020	Not defined	Not reported	Not reported
Hendrix 2020	Not defined	Not reported	Not reported
Hirschman 2020	**Close contact:** Within 6 feet of an infected person for at least 15 minutes starting from 2 days before illness onset.	Not reported	"Hours"
Hobbs 2020	**Close contact:** Within 6 feet for ≥15 minutes) with a person with known COVID- 19, school or child care attendance, and family or community exposures ≤14 days before the SARS-CoV-2 test	Not reported	Within 2 m for ≥15 minutes
Hoehl 2020	Not defined	Not reported	Not reported
Hong 2020	Anyone who ever came within 2 m of a diagnosed patient without the use of effective personal protective equipment	Not reported	258 person-days
Hu 2020	**Close contacts** were defined as individuals who had close-proximity interactions (within 1 meter) with clinically suspected and laboratory-confirmed SARS-CoV-2 cases, for the period from 2 days before, to 14 days after, the potential infector’s symptom onset. For those exposed to asymptomatic subjects, the contact period was from 2 days before, to 14 days after, a respiratory sample was taken for real-time RT-PCR testing. Close contacts included, but were not limited to, household contacts (i.e., household members regularly living with the case), relatives (i.e., family members who had close contacts with the case but did not live with the case), social contacts (i.e., a work colleague or classmate), and other close contacts (i.e., caregivers and patients in the same ward, persons sharing a vehicle, and those providing a service in public places, such as restaurants or movie theatres)	Not reported	Not reported
Hua 2020	Not defined	Not reported	
Huang 2020	**Close contacts** quarantined at home or hospital	Not reported	Not reported
Huang 2020a	Not defined	Not reported	Not reported
Islam 2020	Close contact was defined as individuals who were closely linked by contact tracing and were considered a close contact group provided that no PPE was worn having direct face to face contacts.	Household contacts were defined as individuals who lived and were sharing the same room and same apartment in the same household. Family contacts were those who are the members of the same family but not living in the same household.	Face-to-face
Jia 2020	A **close contact** was defined as a person who did not take effective protection against a suspected or confirmed case 2 d before the onset of symptoms or an asymptomatic infected person 2 d before sampling.	Not reported	Not reported
Jiang 2020	**Close contacts:** Lived with the patients and individuals who had contact with the patients within 1 meter without wearing proper personal protection. Ct value ≥40 was considered negative. The maximum likelihood phylogenetic tree of the complete genomes was conducted by using RAxML software with 1000 bootstrap replicates, employing the general time-reversible nucleotide substitution mode	Not reported	1 m
Jing 2020	A **close contact** was defined as an individual who had unprotected close contact (within 1 m) with a confirmed case within 2 days before their symptom onset or sample collection. Individuals who were linked by contact tracing were considered a close contact group	Not reported	Not reported
Jing 2020a	Not defined	Not reported	Not reported
Jones 2020	**Close contacts** were defined by analysis of video footage for player interactions and microtechnology (GPS) data for proximity analysis.	Not reported	Within 1 m, face-to-face for ≥3 secs
Kang 2020	Not defined	Not reported	Not reported
Kant 2020	Not defined	Not reported	Not reported
Kawasuji 2020	Not defined	Not reported	Not reported
Khanh 2020	**Close contact:** <2 m distance for >15 minutes. Successfully traced passengers and crew members were interviewed by use of a standard questionnaire, tested for SARS-CoV-2	Not reported	<2 m distance for >15 minutes.
Kim 2020	Not defined	Household contact: Occurring at least 1 day after but within 14 days from the last point of exposure.	2 days during the presymptomatic period and 1 day during the symptomatic period of the index case.
Kim 2020a	Not defined	Not reported	2 hrs to 4 days
Kim 2020b	**Contact** was defined as presence in the same room with COVID-19 confirmed patients, or in the same outpatient clinic or examination room, 30 minutes before and after COVID-19 confirmed patients. Within 2 m of confirmed patients (via CCTV)	Not reported	Within 2 m of confirmed patients for 30 mins
Kumar 2020	Not defined	Not reported	Not reported
Kuwelker 2020	Not defined	Household members were defined as individuals who resided in the same household as the index case.	Not reported
Kwok 2020	**Close contacts** referred to anyone who: (i) provided care to the case (including a family member or healthcare worker) or had other close physical contact; or (ii) stayed at the same place (including household members or visitors) while the case was ill.	Not reported	Not reported
Ladhani 2020	Not defined	Not reported	Not reported
Ladhani 2020a	Not defined	Not reported	Not reported
Laws 2020	Not defined	Not reported	Unclear
Laxminarayan 2020	High-risk contacts had close social contact or direct physical contact with index cases without protective measures High-risk travel exposures—defined as close proximity to an infected individual in a shared conveyance for ≥6 hours	Low-risk contacts were in the proximity of index cases but did not meet criteria for high-risk exposure	Not reported
Lee 2020	Not defined: Frequent close contact	Not reported	>1 m
Lee 2020a	Close contact (household contact)	Not reported	Mean contact period was calculated to be 7.7 days.
Lewis 2020	Not defined	Household contacts were defined as all persons living in the same household as the primary patient.	Not reported
Li 2020	Not defined	Not reported	Unclear
Li 2020a	Not defined	Not reported	Not reported
Li 2020b	**Close contact** was defined as an act of sharing a meal, party, vehicle or living room with a confirmed or latently infected patient within 14 days.	Not reported	Not reported
Li 2020c	**Close contacts** were mainly those who have not take effective protection from close contact with the suspected and confirmed cases 2 days before symptoms appeared, or the asymptomatic infected persons 2 days before the specimen collection.	Not reported	Not reported
Li 2020d	Not defined	Not reported	Not reported
Liu 2020	Not defined	Not reported	Unclear
Liu 2020a	Direct contact with patients with neo-coronary pneumonia (within 1 m)	Not reported	Within 1m for 2.5 hrs
Liu 2020b	**Close contacts** were defined by the China Prevention and Control Scheme of COVID-19.	Not reported	7.8 (95%CI: 7.0–8.7) close contacts per index case.
Liu 2020c	Not defined	Not reported	Not reported
López 2020	**Close contact:** Anyone who was within 6 feet of a person with COVID-19 for at least 15 minutes ≤2 days before the patient’s symptom onset.	Not reported	≤1.83m of a person with COVID-19 for at least 15 minutes ≤2 days before the patient’s symptom onset
Lopez Bernal 2020	Household contacts were defined as those living or spending significant time in the same household. Household contacts, others with direct face to face contact and healthcare workers who had not worn recommended PPE	Not reported	Not reported
Lucey 2020	**Close contact:** HCW or patient who spent more than 15 minutes face-to-face within 2 metres of a confirmed case or patients who shared a multi-bedded room with a confirmed case for more than 2 hours.	Not reported	Not reported
Luo 2020	Unclear: The tour coach was with 49 seats was fully occupied with all windows closed and the ventilation system on during the 2.5-hour trip.	Not reported	1 to 4.5m; up to 2.5 hours on a bus
Luo 2020a	**Close contacts:** Anyone who has had contact, without effective protection regardless of duration of exposure, with 1 or more persons with suspected or confirmed COVID-19 any time starting 2 days before onset of symptoms in persons with a suspected or confirmed case, or 2 days before sampling for laboratory testing of asymptomatic infected persons.	Not reported	Not reported
Lyngse 2020	Not defined	Not reported	Not reported
Ma 2020	Not defined	Not reported	Longest contact time: 8 days Shortest contact time: 0 days
Macartney 2020	**Close contacts:** Children or staff with face-to-face contact for at least 15 min, or who shared a closed indoor space for at least 40 min with a case during their infectious period.	Not reported	Face-to-face contact for at least 15 min, or who shared a closed indoor space for at least 40 min
Malheiro 2020	**Close contacts** (high risk)were defined as individuals who have spent 15 min or more in closeproximity (2 m or less) to, or in a closed space with, a case.	Not reported	Not reported
Maltezou 2020	**Close contact** was defined as a contact of >15 minutes within a distance of <2 m with a COVID-19 case.	Household members were defined as persons living in the same residence.	>15 minutes within <2 m
Maltezou 2020a	**Close contact** was defined as a contact of >15 minutes within a distance of <2 meters with a COVID-19 case	Household contacts were defined as persons either living in the same residence or having close contacts with a family member for >4 hours daily in the family residence.	Household: >4 hours daily Close contact: >15 minutes within <2 m
Mao 2020	Not defined	Not reported	Not reported
Martinez-Fierro 2020	Individual who has had closer than <6 feet for ≥15 min with people with a positive diagnosis for COVID-19, whether they were symptomatic or asymptomatic according to the CDC definition	Not reported	≥15 min at a distance of <1.83m
Mponponsuo 2020	An interaction of >15 minutes at a distance of <1 m	Not reported	>15 minutes at a distance of <1 m
Ng 2020	**Close contacts** were individuals who had contact for at least 30 min within a 2 m distance from the index case.	**Work contacts** were defined as individuals who came into close contact with the index case at work, from 2 days before the onset of symptoms to isolation of the case, to account for pre-symptomatic transmission. **Social contacts** were defined as individuals who came into close contact with the index case, from 2 days before onset of symptoms to isolation of the case, through social activities. Transport contacts were excluded **Lower risk contacts:** Other contacts who were with the index case for 10–30 min within 2 m	At least 30 min within a 2 m
Ning 2020	Not defined	Not reported	Unclear
Njuguna 2020	Not defined	Not reported	Unclear
Ogawa 2020	Not defined	Not reported	Not reported
Paireau 2020	Not defined	Not reported	Not reported
Park 2020	Not defined	Not reported	Not reported
Park 2020a	High-risk contact (household contacts of COVID-19 patients, healthcare personnel)	Household contact was a person who lived in the household of a COVID-19 patient and a nonhousehold contact was a person who did not reside in the same household as a confirmed COVID-19 patient.	Not reported
Park 2020b	Not defined	Not reported	Not reported
Passarelli 2020	Not defined	Not reported	Not reported
Patel 2020	Not defined	Not reported	Not reported
Pavli 2020	**Close contacts** were defined as persons sitting within a distance of <2 m for >15 min, including passengers seated two seats around the index case and all crew members and persons who had close contact with the index case.	Not reported	<2 m for >15 min
Phiriyasart 2020	Close contact was defined as a person who had at least one of these following criteria : (i) a person who came into close (within 1 meter) contact with, or had a conversation with any patient for >5 minutes, or was coughed or sneezed on by any patient when he/she did not wear appropriate personal protective equipment (PPE), e.g. a face mask, (ii) a person who was in an enclosed space without proper ventilation, e.g. in the same air-conditioned bus/air-conditioned room as any patient , and was within one meter of any patient for >15 minutes without wearing appropriate PPE. High-risk close contact was defined as a close contact who was likely to contract the virus from any patient through exposure to respiratory secretions of any patient while not wearing PPE according to standard precautions.	A low-risk close contact was defined as a close contact who was less likely to contract the virus from any patient. This includes close contacts who have not met the definition for high-risk close contacts.	Not reported
Poletti 2020	Not defined	Not reported	Not reported
Pung 2020	**Close contacts:** People who spend a prolonged time within 2 m of a confirmed case	**Other contacts:** People who had some interactions with the case.	Unclear
Pung 2020a	Unclear: Close household contacts	Not reported	Unclear
Qian 2020	Four categories of infected individuals were considered based on their relationship: family members, family relatives, socially connected individuals, and socially non‐connected individuals	Not reported	Not reported
Ravindran 2020	**Close contact:** Face-to-face contact for greater than 15 minutes cumulative in the period extending from 48 hours before onset of symptoms in a confirmed case; or sharing of closed space with a confirmed case for a prolonged period of time in the period extending from 48 hours before onset of symptoms in a confirmed case.	Not reported	Face-to-face contact for at least 15 min, or who shared a closed indoor space for prolonged period 48 hrs before onset of symptoms
Razvi 2020	Not defined	Not reported	Not reported
Rosenberg 2020	Not defined	Not reported	
Roxby 2020	Not defined	Not reported	Not reported
Sang 2020	Not defined	Not reported	Not reported
Schumacher 2020	Close contact: Approximately 30–90 seconds in close proximity (<1.5 m) of other players	Close social contacts (including sharing a car)	30–90 seconds in close proximity (<1.5 m)
Schwierzeck 2020	Not defined	Not reported	Not reported
Shah 2020	Household contact was defined as contact sharing same residential address.	Not reported	Not reported
Shen 2020	**Close contacts** defined as individuals who had close, prolonged, and repeated interactions with the 2 source cases (Cases 2 and 3).	All other contacts are defined as casual contacts.	Not reported
Sikkema 2020	Not defined	Not reported	Not reported
Son 2020	Not defined	A contact was defined as anyone who was in contact with a confirmed case from a day before the symptoms occurred, in a manner that offered the potential for transmission through respiratory droplets	Not reported
Song 2020	Unclear: shared the same bedroom, had dinner together	Not reported	Not reported
Speake 2020	2 rows in front and behind infectious passenger on an airplane	Not reported	Unclear
Stein-Zamir 2020	Not defined	Not reported	Not reported
Sugano 2020	Not defined	Not reported	Unclear
Sun 2020	Not defined	Not reported	Not reported
Taylor 2020	Not defined	Not reported	Unclear
Teherani 2020	Household contacts (HCs) were defined as an adult ( 18 years) or a child (<18 years) who resided in the home with the SIC at the time of diagnosis.	Not reported	Not reported
Thangaraj 2020	Not defined	Not reported	Unclear
Torres 2020	Not defined	Not reported	Unclear
Tshokey 2020	Unclear: Close friends, roommates, flight seat partner, spouse or partner, cousin, physician, tour driver	**Primary contacts:** Individuals coming in some form of contact with the confirmed cases such as conveyance in the same cars/flights, encounter in clinics, serving meals, or providing housekeeping services in hotels. **Secondary contacts:** Individuals coming in contact with the primary contacts	Unclear
van der Hoek 2020	Not defined	Not reported	
Wang 2020	Not defined	Not reported	Unclear
Wang 2020a	Not defined	Not reported	Unclear
Wang 2020b	Close contact was defined as being within 1 m or 3 feet of the primary case, such as eating around a table or sitting together watching TV.	Not reported	Unclear
Wee 2020	Not defined	Not reported	Within 2 m of the index case for a cumulative time of ≥15 minutes, or who had performed AGPs without appropriate PPE.
Wendt 2020	**High-risk contacts:** >15 min face-to-face contact, sitting in a row behind physician for 45 mins, transfer in an ambulance (45-min drive).	Not reported	>15 min face-to-face contact
Wolf 2020	Not defined	Not reported	Not specified
Wong 2020	**Contact case** was defined as a patient or staff who stayed or worked in the same ward as the index patient. Patients who shared the same cubicle with the index case were considered as ‘patient close contact’. **Staff close contact:** Staff who had contact within 2 m of the index case for a cumulative time of >15 min, or had performed AGPs, without ‘appropriate’ PPE.	Casual contacts: All staff and patients who did not fulfill the pre-defined criteria for close contacts. Casual/low-rosk contact: HCW wearing a facemask or respirator only and have prolonged close contact with a patient who was wearing a facemask, or HCW using all recommended PPE or HCW (not using all recommended PPE) who have brief interactions with a patient regardless of whether patient was wearing a facemask. Patient close contacts were quarantined into an AIIR (or quarantine camp if the patient was deemed clinically stable to be discharged from hospital) for 14 days.	Within 2 m of the index case for a cumulative time of >15 min
Wood 2020	Not defined	Not reported	Not reported
Wu 2020	**Close contact:** Been within 1 metre of a confirmed case, without effective PPE, within the period since 5 days before the symptom onset in the index case or since 5 days before sampling if the index case was asymptomatic.	Not reported	Within 1 metre of a confirmed case, without effective PPE
Wu 2020a	Household contacts were defined as person who spent at least 1 night in the house after the symptom onset of the index patient. A household was defined as ≥2 people living together in the same indoor living space. A household index was the first person to introduce SARS-CoV-2 into the household.	Not reported	At least 1 night
Xie 2020	**Close contact:** An individual who has not taken effective protection when in proximity of suspected or confirmed cases 2 days before the onset of symptoms or 2 days before the collection of asymptomatic specimens.	Not reported	Unclear
Xin 2020	Close contacts were defined as persons who had a short‐range contact history for 2 days before the onset of symptoms in COVID‐19‐suspected and ‐confirmed cases, or 2 days before the collection of samples from asymptomatic cases without taking effective protective measures, such as family members in the same house, direct caregivers, and medical staff who provided direct medical care, colleagues in the same office or workshop, etc.	The effective contact duration for the close contacts was defined as the contact days with index patients with confirmed COVID‐19, which was calculated as the last contact date minus the start contact date, and all dates were corresponding to the definition of close contacts	The median effective contact duration with patients with COVID‐19 was 4 (IQR: 1–6) days, with 57 (53.8%) experiencing effective contact between 3 and 11 days, and 9 (8.5%) with effective contact duration > 11 days
Yang 2020	**Close contacts:** Uunprotected exposure.	Candidate contacts: Teachers and classmates	Not reported
Yau 2020	Close unprotected contact with someone who has tested positive for COVID-19 in the last 14 days	Not reported	Unclear
Ye 2020	Not defined	Not reported	Not reported
Yoon 2020	**Close contact** was defined as a person who had face-to-face contact for >15 minutes or who had direct physical contact with the index case-patient. Persons who used the same shuttle bus were also considered to be close contacts.	Not reported	Face-to-face contact for >15 minutes or direct physical contact
Yousaf 2020	Not defined	Not reported	Not reported
Yu 2020	**Close contacts** were defined as those who lived in the same household, shared meals, traveled or had social interactions with a confirmed case two days before the onset of COVID-19 symptoms	Not reported	Not reported
Yung 2020	Not defined	Not reported	Not reported
Zhang 2020	Not defined	Not reported	Not reported
Zhang 2020a	**Close contact:** Refers to a person who had contact with index case without using proper protection during 2 days before the index case was tested.	Not reported	Not reported
Zhang 2020b	Not defined	Not reported	Not reported
Zhang 2020c	**Close contacts** were individuals who lived with a PCR-confirmed case or interacted with a case within 1 metre from the case without any personal protections.	Not reported	Within 1m of case
Zhang 2020d	Not defined	Not reported	Not reported
Zhuang 2020	Not defined	Not reported	Not reported

Eighteen studies (10.5%) reported data on the contact duration between close contacts and the index or primary cases ( Table 3). The average contact duration ranged from 30 minutes to 8 days across 16 studies that investigated transmission rates using RT-PCR. In two studies that examined transmission using serology (Agergaard 2020, Hong 2020), the durations of contact were two weeks and 258 person-days, respectively. The mean contact duration was either unclear or not reported in 148 studies (90.2%).

A total of 110 studies (64.3%) used RT-PCR as a test method for confirming SARS-CoV-2 positivity, while eight studies (4.8%) exclusively investigated transmission using serology. In 24 studies (14%), both PCR and serology were used to investigate close contact in SARS-CoV-2 transmission. Thirty-one studies (18.1%) did not report the test method used. For PCR, the timing of sample collection varied from within 24 hours to 14 days after exposure to the index or primary case; for serology, this ranged from 2–10 weeks post-exposure. In total, 71 studies (41.5%) reported the timing of sample collection. The timing of sample collection was either not reported or unclear in 100 studies (58.5%).

Twenty-two studies (12.9%) reported Ct values for determining PCR test positivity: ≤40 (eight studies), <37 (five studies), ≤35 (three studies), <38 (two studies), one each for <25, ≤30, <32 and <36 (or 39). Only eight studies reported the Ct values for close contacts in their results – these ranged from 16.03 to 38.

Thirty-two studies reported conducting serological tests to assess transmission of SARS-CoV-2 ( Table 4). There was variation in the description of the tests. Fifteen studies determined the antibody responses to SARS-CoV-2 spike proteins using Immunoglobulin G (IgG) and IgM while 11 used only IgG. In 17 studies, the threshold for serological positivity was not reported. Three studies (Kuwelker 2020, Ng 2020, Yang 2020) performed neutralisation assays to confirm positive serologic samples. In one study (Torres 2020), study participants self-administered the serological tests.

**Table 4.** Description of serological tests in close contact studies of SARS-CoV-2.

Study ID	Serological test	Description of test	Thresholds for serological positivity
Agergaard 2020	IgG and IgM	iFlash and DiaSorin	iFlash SARS-CoV-2 N/S IgM/IgG cut-off: ≥12 AU/ml = positive. DiaSorin SARS-CoV-2 S1/S2 IgG cut-off: ≥15 AU/ml = positive, 12 < x < 15 AU/ml = equivocal, and ≤12 AU/ml = negative.
Angulo-Bazán 2020	IgG and IgM	Coretests ® COVID-19 IgM / IgG Ab Test (Core Technology Co. Ltd), a lateral flow immunochromatographic test that qualitatively detects the presence of antibodies against SARS-CoV-2, with a sensitivity and specificity reported by the manufacturer for IgM / IgG of 97.6% and 100%, respectively	Not reported
Armann 2020	IgG	Diasorin LIAISON® SARS-CoV-2 S1/S2 IgG Assay). All samples with a positive or equivocal LIAISON® test result, as well as all samples from participants with a reported personal or household history of a SARS-CoV-2 infection, were re-tested with two additional serological tests: These were a chemiluminescent microparticle immunoassay (CMIA) intended for the qualitative detection of IgG antibodies to the nucleocapsid protein of SARS-CoV-2 (Abbott Diagnostics® ARCHITECT SARS-CoV-2 IgG ) (an index (S/C) of < 1.4 was considered negative whereas one >/= 1.4 was considered positive) and an ELISA detecting IgG against the S1 domain of the SARS- CoV-2 spike protein (Euroimmun® Anti-SARS-CoV-2 ELISA) (a ratio < 0.8 was considered negative, 0.8–1.1 equivocal, > 1.1 positive) Participants whose positive or equivocal LIAISON® test result could be confirmed by a positive test result in at least one additional serological test were considered having antibodies against SARS-CoV_x0002_2.	Antibody levels > 15.0 AU/ml were considered positive and levels between 12.0 and 15.0 AU/ml were considered equivocal.
Baettig 2020	IgG and IgM	Used commercially available immunochromatography rapid test with SARS-CoV-2 protein-specific IgM and IgG. This test was performed according to the manufacturers’ instructions with a reported sensitivity and specificity of 93% and 95%, respectively.	Not reported
Basso 2020	IgG and IgM	Sera were collected approximately 3 weeks following exposure for the detection of antibodies against SARS-CoV-2. EDI Novel Coronavirus COVID-19 lgG and IgM ELISA (Epitope Diagnostics, Inc., San Diego, CA, USA) were used for initial testing, and supplemented with tests from DiaSorin (LIAISON SARS-CoV-2 S1/S2 IgG test), Abbott (Alinity i SARS-CoV-2 IgG), Roche (Elecsys Anti- SARS-CoV-2) and Wantai (WANTAI SARS-CoV-2 Ab ELISA).	Not reported
Brown 2020	IgG and IgM	ELISA (authors referenced another study)	Reciprocal titers of >400 to be positive and reciprocal titers of >100 but <400 to be indeterminate.
Chen 2020b	IgG and IgM	In-house enzyme immunoassay (EIA). 96-well plates were coated with 500 ng/mL of recombinant RBD or NP protein overnight, incubating with diluted serum samples at 1:20. Plates were incubated with either anti-human IgM or IgG conjugated with HRP. Optical density (OD) value (450nm-620nm) was measured.	Preliminary cut-off values were calculated as the mean of the negative serum OD values plus 3 standard deviation (SD) from 90 archived healthy individuals in 2019. A close contact was considered seropositive if OD of 1:20 diluted serum was above the cut-off values for either IgM or IgG against both RBD and NP protein
Chu 2020	IgG and IgM	Serum samples were tested at CDC using a SARS-CoV-2 ELISA with a recombinant SARS-CoV-2 spike protein (courtesy of Dr. Barney Graham, National Institutes of Health, Bethesda, MD, USA) as an antigen. Protein ELISA 96-well plates were coated with 0.15 μg/mL of recombinant SARS-CoV-2 spike protein and ELISA was carried out as previously described. An optimal cutoff optical density value of 0.4 was determined for >99% specificity and 96% sensitivity. Serum samples from the case-patient were used as a positive control and commercially available serum collected before January 2020 from an uninfected person as a negative control.	Total SARS-CoV-2 antibody titers >400 were considered seropositive.
Dattner 2020	IgG	Abbott SARS-CoV-2 IgG, whose specificity was estimated as ∼100% and whose sensitivity at ≥ 21 days was estimated as ∼85%	Not reported
de Brito 2020	IgG and IgM	Chemiluminescence 4 weeks after contact with the index case	Not reported
Dimcheff 2020	IgG	Serum IgG to thD4:D12e nucleoprotein of SARS-CoV-2 was measured using a Federal Food and Drug Administration (FDA) emergency-use–authorized chemiluminescent microparticle immunoassay performed on an automated high throughput chemistry immunoanalyzer (Architect i2000SR, Abbott Laboratories, Abbott Park, IL). The sensitivity of this assay is reported to be 100% with a specificity of 99% at >14 days after symptom onset in those infected with SARS-CoV-2.1 At 5% prevalence, the positive predictive value is 93.4% and the negative predictive value is 100%	Results are reported in a relative light units (RLU) index; a value ≥1.4 RLU is considered a positive antibody response.
Dub 2020	IgG	IgG antibodies to SARS-CoV-2 nucleoprotein (The Native Antigen Company, United Kingdom) were measured with a fluorescent bead-based immunoassay (manuscript in preparation). Antigen was conjugated on MagPlex Microspheres and bound IgG antibodies were identified by a fluorescently labeled conjugated antibody (R_x0002_Phycoerythrin- conjugated Goat Anti-Human IgG, Jackson Immuno Research, USA). The plate was read on Luminex® MAGPIX® system. xPONENT software version 4.2 (Luminex®Corporation, Austin, TX) was used to acquire and analyze data. Median fluorescent intensity was converted to U/ml by interpolation from a 5- parameter logistic standard curve. The specificity and sensitivity of the assay was assessed using receiver operator curve (ROC) with 100% specificity and 97.9% sensitivity	MNT titre of ≥ 6 considered positive FMIA titre 3·4 U/ml considered positive
Fontanet 2020	IgG	Antibody responses to SARS-CoV-2 using several assays developed by Institut Pasteur : an ELISA N assay, detecting antibodies binding to the N protein; a S-Flow assay, which is a flow-cytometry based assay detecting anti-S IgG; and a LIPS assay, which is an immunoprecipitation-based assay detecting anti-N and anti-S1 IgG.	Participants were considered seropositive for SARS-CoV-2 if any test was positive, since all tests had a specificity higher than 99% with the cut-offs chosen for positivity
Fontanet 2020a	Not specified	Serological testing was conducted using the S-Flow assay, a flow_x0002_cytometry-based serological test developed by the Institut Pasteur. The assay is based on the recognition of the SARS-CoV-2 Spike protein expressed at the surface of 293T cells. In previous studies, the sensitivity of the assay was estimated at 99.4% (95% CI = 96.6% - 100%) on a panel of 160 RT-PCR confirmed mild forms of COVID-1928, while its specificity was found to be 100% (one-sided 97.5% CI = 97.4% - 100%) on a panel of 140 pre-epidemic sera	Not reported
Gu 2020	IgG	Not described	Not reported
Helsingen 2020	IgG	Measurement of IgG antibodies was performed with a multiplex flow cytometric assay known as microsphere affinity proteomics (MAP)	Not specified. Referenced
Hong 2020	IgG and IgM	Qualitative colloidal gold assay (Innovita (Tangshan) Biological Technology, Co., Ltd, Tangshan, China), following manufacturers’ instructions. The sensitivity of the assay was 87.3% (95%CI 80.4–92.0%), and the specificity was 100% (95%CI 94.20–100%) according to the instructions of the assay.	Not reported
Kuwelker 2020	IgG	A two-step ELISA was used for detecting SARS-CoV-2-specific antibodies, initially by screening with receptor-binding domain (RBD) and then confirming seropositivity by spike IgG. Endpoint titres were calculated as the reciprocal of the serum dilution giving an optical density (OD) value=3 standard deviations above the mean of historical pre-pandemic serum samples. Individuals with no antibodies were assigned a titre of 50 for calculation purposes. **Neutralisation assays were used to quantify SARS-CoV-2-specific** ** functional antibodies.** VN titres were determined as the reciprocal of the highest serum dilution giving no CPE. Negative titres (<20) were assigned a value of 10 for calculation purpose.	Not specified.
Lewis 2020	Not specified	ELISA (authors referenced another study)	Not specified
Luo 2020a	IgG and IgM	Not described	Asymptomatic: Specific IgM detected in serum. Symptomatic: Detectable SARS-CoV-2–specific IgM and IgG in serum, or at least a 4-fold increase in IgG between paired acute and convalescent sera.
Macartney 2020	IgA, IgG, IgM	SARS-CoV-2-specific IgG, IgA, and IgM detection was done using an indirect immunofluorescence assay (IFA) that has a sensitivity compared with nucleic acid testing of detecting any of SARS-CoV-2-specific IgG, IgA, or IgM when samples were collected at least 14 days after illness onset of 91·3% (95% CI 84·9–95·6) and specificity of 98·9% (95% CI 98·4–99·3%; MVNO, personal communication).	Not specified
Martinez-Fierro 2020	IgG and IgM	IgM and IgG against SARS-CoV-2 were determined using a total blood sample through a 2019 nCov IgG/IgM rapid test (Genrui Biotech, Shenzen, China)	Not specified
Ng 2020	Not specified	human ACE-2 (hACE2) protein (Genscript Biotech, New Jersey, United States) was coated at 100 ng/well in 100 mM carbonate- bicarbonate coating buffer (pH 9.6). 3ng of horseradish peroxidase (HRP)-conjugated recombinant receptor binding domain (RBD) from the spike protein of SARS-CoV-2 (GenScript Biotech) was pre-incubated with test serum at the final dilution of 1:20 for 1 hour at 37°C, followed by hACE2 incubation for 1 h at room temperature.Serum samples were tested with a surrogate **viral neutralising** **assay** for detection of neutralising antibodies to SARS-CoV-2.	A positive serological test result was concluded if the surrogate viral neutralising assay for a particular sample resulted in inhibition of 30% or greater (98·9% sensitivity and 100·0% specificity)
Ogawa 2020	IgG	Abbott® (Abbott ARCHITECT SARS-CoV-2 IgG test, Illinois, USA)	Not specified
Poletti 2020	IgG	Not described	Not specified
Razvi 2020	IgG and IgM	Blood samples were analysed on the day of collection using the Roche Elecsys Anti-Sars-CoV-2 serology assay. This electrochemiluminescent immunoassay is designed to detect both IgM and IgG antibodies to SARS-CoV-2 in human serum and plasma and has been shown to have a high sensitivity and specificity	Not specified
Schumacher 2020	IgG and IgM	SARS-CoV-2-specific antibodies were measured in serum samples using an electrochemiluminescence immunoassay (Elecsys® Anti-SARS-CoV-2, Roche Diagnostics, Rotkreuz, Switzerland).	Cut-off indices ≤1 reported as negative and indices >1 as positive.
Torres 2020	IgG and IgM	Novel Coronavirus (2019-nCoV) IgG/IgM Test Kit (Colloidal gold) from Genrui Biotech Inc. The study nurse and/or technician viewed the photo provided by the participant along with the participant’s self-report as to the visibility of the three bands, and determined whether the tests were IgG+, IgM+, IgG & IgM+, Negative, Invalid, or Indeterminate. Participants were asked to attach a photo of the test after 15 minutes had elapsed and self-report the appearance of the three lines, G (IgG), M (IgM), and C (test control)	Colour-coded - self-administered test: self-reporting the appearance of the three lines, G (IgG), M (IgM), and C (test control)
van der Hoek 2020	IgG	Fluorescent bead-based multiplex-immunoassay. Referenced	A cut-off concentration for seropositivity (2.37 AU/mL; with specificity of 99% and sensitivity of 84.4%) was determined by ROC-analysis of 400 pre-pandemic control samples
Wendt 2020	IgA and IgG	ELISA (Euroimmun, Lübeck, Germany), following the manufacturer’s instructions.	Inconclusive (≥0.8 and <1.1) or Positive (≥1.1
Yang 2020	IgA, IgG, IgM	Serum immunoglobulin (Ig) antibody against the SARS-CoV-2 surface spike protein receptor-binding domain (RBD) was measured using a chemiluminescence kit (IgM, IgG, and total antibody, Beijing Wantai Biotech, measured by cut-off index [COI]) or ELISA kit (IgA, Beijing Hotgen Biotech, measured by optical density at 450/630 nm [OD450/630]). The cut-off for seropositivity was set according to the manufacturer’s instruction, verified using positive (169 serum specimens from confirmed COVID-19 patients) and negative (128 serum specimens from healthy persons) controls, and both of sensitivity and specificity were 100%. **Virus neutralization assays** were performed using SARS-CoV-2 virus strain 20SF014/vero-E6/3 (GISAID accession number EPI_ISL_403934) in biosafety level 3 (BSL-3) laboratories. Neutralizing antibody (NAb) titer was the highest dilution with 50% inhibition of cytopathic effect, and a NAb titer of ≥1:4 was considered positive.	Specimens with COI>1 (IgM, IgG, or total antibody), OD450/630 > 0.3 (IgA) were considered positive.
Zhang 2020b	IgG and IgM	SARS-CoV-2-specific IgM and IgG were tested by paramagnetic particle chemiluminescent immunoassay using iFlash-SARS-CoV-2 IgM/IgG assay kit (Shenzhen YHLO Biotech Co., Ltd) and iFlash Immunoassay Analyzer (Shenzhen YHLO Biotech Co., Ltd). The specificity and sensitivity of SARS-CoV-2 IgM and IgG detection were also evaluated	Not specified

**Table 5.** Quality of included studies.

Study	Description of methods and sufficient detail to replicate	Sample sources clear	Analysis & reporting appropriate	Is bias dealt with	Applicability	Notes
Abdulrahman 2020	Unclear	Yes	Yes	No	Yes	
Adamik 2020	Unclear	Unclear	Yes	No	Unclear	
Agergaard 2020	No	Yes	Yes	No	Yes	
Angulo-Bazán 2020	Yes	No	Yes	Unclear	Yes	
Armann 2020	Unclear	Yes	Yes	No	Yes	
Arnedo-Pena 2020	Yes	Yes	Yes	Unclear	Yes	
Baker 2020	Unclear	Yes	Yes	Unclear	Yes	
Baettig 2020	Unclear	Yes	Yes	Unclear	Yes	
Bao 2020	Unclear	Yes	Yes	No	Yes	
Basso 2020	Unclear	Yes	Yes	Unclear	Yes	
Bays 2020	Unclear	Yes	Yes	No	Yes	
Bi 2020	Yes	Yes	Yes	Unclear	Yes	
Blaisdell 2020	Yes	No	Yes	Unclear	Yes	
Böhmer 2020	Yes	Yes	Yes	Unclear	Yes	
Boscolo-Rizzo 2020	Unclear	Yes	Yes	No	Yes	
Brown 2020	Yes	Yes	Yes	Unclear	Unclear	
Burke 2020	Unclear	No	Yes	No	Yes	
Canova 2020	Unclear	Yes	Yes	Unclear	Yes	
Cariani 2020	Unclear	Yes	Unclear	Unclear	Yes	
Charlotte 2020	Unclear	Yes	Yes	Unclear	Yes	
Chaw 2020	Unclear	Yes	Yes	Unclear	Yes	
Chen 2020	Unclear	Unclear	Yes	No	Unclear	
Chen 2020a	Unclear	Yes	Yes	Unclear	Yes	
Chen 2020b	Yes	Yes	Yes	Unclear	Yes	
Chen 2020c	Unclear	No	Yes	No	Yes	
Cheng 2020	Yes	No	Yes	Unclear	Yes	
Chu 2020	Yes	Yes	Yes	Unclear	Yes	
Chu 2020a	Unclear	Unclear	Unclear	No	Yes	
Contejean 2020	Unclear	Yes	Yes	Unclear	Yes	
COVID-19 National Emergency Response Center 2020	Unclear	No	Yes	No	Yes	
Danis 2020	Yes	Yes	Yes	No	Yes	
Dattner 2020	Yes	Yes	Yes	Unclear	Yes	
de Brito 2020	Yes	Yes	Unclear	Unclear	Yes	
Deng 2020	Unclear	No	Unclear	Unclear	Unclear	
Desmet 2020	Yes	Yes	Yes	No	Unclear	
Dimcheff 2020	Yes	Unclear	Yes	Unclear	Unclear	
Dong 2020	Unclear	No	Unclear	No	Yes	
Doung-ngern 2020	Yes	Yes	Yes	Unclear	Yes	
Draper 2020	Yes	Yes	Yes	No	Yes	
Dub 2020	Yes	Yes	Yes	Unclear	Yes	
Expert Taskforce 2020	Unclear	Unclear	Yes	Unclear	Unclear	
Fateh-Moghadam 2020	Unclear	No	Yes	No	Yes	
Firestone 2020	Unclear	Unclear	Yes	Unclear	Yes	
Fontanet 2020	Yes	Yes	Yes	No	Yes	
Fontanet 2020a	Yes	Yes	Yes	No	Yes	
Gan 2020	Unclear	Unclear	Unclear	Unclear	Unclear	
Ghinai 2020	Unclear	Unclear	Unclear	Unclear	Unclear	
Gong 2020	Yes	Yes	Unclear	Unclear	Unclear	
Gu 2020	Unclear	Unclear	Unclear	No	Unclear	
Hamner 2020	Unclear	Unclear	Yes	No	Yes	
Han 2020	Yes	Yes	Yes	Unclear	Yes	
Heavey 2020	Unclear	No	Yes	No	Yes	
Helsingen 2020	Yes	Yes	Yes	Yes	Yes	
Hendrix 2020	Yes	Yes	Yes	No	Yes	
Hirschman 2020	Unclear	Unclear	Unclear	No	Yes	
Hobbs 2020	Yes	Yes	Yes	Unclear	Yes	
Hoehl 2020	Yes	Yes	Yes	Unclear	Yes	
Hong 2020	Yes	Yes	Yes	Unclear	Yes	
Hu 2020	Unclear	No	Yes	No	Yes	
Hua 2020	Yes	Unclear	Yes	Unclear	Yes	
Huang 2020	Unclear	Unclear	Yes	No	Unclear	
Huang 2020a	Unclear	Unclear	Yes	Unclear	Unclear	
Islam 2020	Yes	No	Yes	No	Yes	
Jia 2020	Unclear	Unclear	Yes	No	Unclear	
Jiang 2020	Yes	Yes	Unclear	No	Yes	
Jing 2020	Yes	Yes	Yes	Unclear	Yes	
Jing 2020a	Unclear	Yes	Unclear	Unclear	Unclear	
Jones 2020	Unclear	Yes	Yes	Unclear	Unclear	
Kang 2020	Unclear	Unclear	Unclear	Unclear	Unclear	
Kant 2020	Unclear	Yes	Unclear	No	Unclear	
Kawasuji 2020	Unclear	Yes	Unclear	Unclear	Unclear	
Khanh 2020	Yes	Yes	Yes	No	Yes	
Kim 2020	Unclear	Yes	Yes	Unclear	Yes	
Kim 2020a	Unclear	Yes	Yes	No	Unclear	
Kim 2020b	Yes	Yes	Yes	No	Yes	
Kumar 2020	Unclear	Yes	Unclear	No	Unclear	
Kuwelker 2020	Unclear	Yes	Yes	Unclear	Yes	
Kwok 2020	Unclear	Unclear	Yes	Unclear	Unclear	
Ladhani 2020	No	Unclear	Unclear	No	Yes	
Ladhani 2020a	Unclear	Unclear	Yes	Unclear	Yes	
Laws 2020	Unclear	Unclear	Yes	Unclear	Yes	
Laxminarayan 2020	Yes	No	Yes	No	Yes	
Lee 2020	Unclear	Unclear	Yes	Unclear	Unclear	
Lee 2020a	Unclear	No	Yes	No	Yes	
Lewis 2020	Yes	Yes	Yes	No	Yes	
Li 2020	Unclear	Yes	Unclear	No	Unclear	
Li 2020a	Unclear	Unclear	Unclear	Unclear	Unclear	
Li 2020b	Unclear	Yes	Unclear	Unclear	Unclear	
Li 2020c	Unclear	No	Unclear	Unclear	Unclear	
Li 2020d	Yes	Yes	Yes	No	Yes	
Liu 2020	Unclear	Unclear	Unclear	No	Yes	
Liu 2020a	Yes	Yes	Yes	Unclear	Unclear	
Liu 2020b	Unclear	Yes	Yes	Unclear	Yes	
Liu 2020c	Unclear	Unclear	Unclear	No	Unclear	
López 2020	Unclear	Unclear	Yes	Unclear	Yes	
Lopez Bernal 2020	Yes	Unclear	Yes	No	Yes	
Lucey 2020	Unclear	Yes	Yes	No	Yes	
Luo 2020	Unclear	Yes	Yes	Unclear	Yes	
Luo 2020a	Unclear	Yes	Yes	Yes	Yes	They use multiple imputation to minimise inferential bias, and they discuss recall bias, selection bias and regression to the mean.
Lyngse 2020	Yes	Unclear	Yes	Yes	Yes	They investigate bias within their data and discuss this fairly fully
Ma 2020	Unclear	Unclear	Unclear	Unclear	Unclear	
Macartney 2020	Yes	Unclear	Yes	Unclear	Yes	
Malheiro 2020	Yes	Unclear	Yes	Unclear	Yes	
Maltezou 2020	Unclear	Unclear	Unclear	Unclear	Yes	
Maltezou 2020a	Unclear	Unclear	Unclear	No	Yes	
Mao 2020	Unclear	Unclear	Yes	No	Unclear	
Martinez-Fierro 2020	Unclear	Yes	Yes	No	Yes	
Mponponsuo 2020	Unclear	Yes	Yes	Yes	Yes	Recall bias was minimized by examining multiple data sources for both index cases and exposed persons
Ng 2020	Unclear	Yes	Yes	Yes	Yes	Authors looked at differences that could have led to bias
Ning 2020	Unclear	Unclear	Unclear	Unclear	Unclear	
Njuguna 2020	Unclear	Unclear	Yes	Unclear	Yes	
Ogawa 2020	Unclear	Unclear	Yes	No	Yes	
Paireau 2020	Unclear	Yes	Yes	Unclear	Yes	
Park 2020	Unclear	Yes	Yes	Unclear	Yes	
Park 2020a	Unclear	No	Yes	No	Yes	
Park 2020b	Unclear	Yes	Yes	No	Unclear	
Passarelli 2020	Unclear	No	Unclear	Unclear	Yes	
Patel 2020	Yes	Yes	Yes	Unclear	Unclear	
Pavli 2020	Unclear	Yes	Yes	No	Yes	
Phiriyasart 2020	Yes	Yes	Yes	No	Yes	
Poletti 2020	Unclear	Yes	Yes	Yes	Unclear	
Pung 2020	Yes	Unclear	Yes	Unclear	Yes	
Pung 2020a	Unclear	No	Unclear	Unclear	Unclear	
Qian 2020	Unclear	Unclear	Unclear	No	Unclear	
Ravindran 2020	Unclear	Unclear	Unclear	Unclear	Unclear	
Razvi 2020	Unclear	Yes	Yes	No	Yes	
Rosenberg 2020	Yes	Yes	Yes	No	Yes	
Roxby 2020	Yes	Yes	Yes	Unclear	Yes	
Sang 2020	Unclear	Yes	Unclear	No	Unclear	
Schumacher 2020	Unclear	Yes	Unclear	Unclear	Yes	
Schwierzeck 2020	Unclear	Yes	Yes	Unclear	Yes	
Shah 2020	Unclear	No	Unclear	No	Yes	
Shen 2020	Yes	Yes	Yes	Unclear	Yes	
Sikkema 2020	Unclear	Yes	Yes	Unclear	Yes	
Son 2020	Unclear	Unclear	Yes	No	Yes	
Song 2020	Unclear	Yes	Yes	Unclear	Yes	
Speake 2020	Unclear	Yes	Yes	Unclear	Yes	
Sugano 2020	Unclear	Unclear	Yes	Unclear	Yes	
Stein-Zamir 2020	Yes	Unclear	Yes	No	Yes	
Sun 2020	Unclear	Unclear	Unclear	Unclear	Unclear	
Taylor 2020	Yes	Yes	Yes	Unclear	Yes	
Teherani 2020	Unclear	Yes	Yes	Unclear	Yes	
Thangaraj 2020	Unclear	Yes	Yes	Unclear	Unclear	
Torres 2020	Yes	Unclear	Yes	Unclear	Yes	
Tshokey 2020	Unclear	Yes	Yes	Unclear	Yes	
van der Hoek 2020	Unclear	Yes	Yes	No	Yes	
Wang 2020	Unclear	Yes	Unclear	Unclear	Yes	
Wang 2020a	Yes	Unclear	Yes	Unclear	Yes	
Wang 2020b	Yes	Yes	Yes	No	Yes	
Wee 2020	Yes	Yes	Yes	Unclear	Yes	
Wendt 2020	Yes	Yes	Yes	Unclear	Yes	
Wolf 2020	Yes	Yes	Yes	Unclear	Yes	
Wong 2020	Yes	Yes	Yes	Unclear	Yes	
Wood 2020	Unclear	No	Yes	Unclear	Yes	
Wu 2020	Yes	Unclear	Yes	Unclear	Yes	
Wu 2020a	Yes	Unclear	Yes	Unclear	Yes	
Xie 2020	Unclear	Yes	Yes	Unclear	Yes	
Xin 2020	Yes	No	Yes	No	Yes	
Yang 2020	Unclear	Yes	Unclear	Unclear	Yes	
Yau 2020	Unclear	Yes	Unclear	Unclear	Unclear	
Ye 2020	Unclear	Unclear	Unclear	Unclear	Unclear	
Yoon 2020	Yes	Yes	Yes	Unclear	Yes	
Yousaf 2020	Unclear	Yes	Unclear	Unclear	Unclear	
Yu 2020	Yes	No	Yes	No	Yes	
Yung 2020	Unclear	Yes	Yes	No	Yes	
Zhang 2020	Unclear	Unclear	Unclear	No	Unclear	
Zhang 2020a	Yes	Unclear	Yes	Unclear	Unclear	
Zhang 2020b	Unclear	Yes	Unclear	Unclear	Yes	
Zhang 2020c	Unclear	Unclear	Unclear	Unclear	Unclear	
Zhang 2020d	Unclear	Yes	Unclear	Unclear	Unclear	
Zhuang 2020	Unclear	No	Yes	No	Unclear	

Three studies (Ladhani 2020a, Speake 2020, Yang 2020) performed viral culture, while 10 studies (Böhmer 2020, Firestone 2020, Jiang 2020, Ladhani 2020a, Lucey 2020, Pung 2020, Sikkema 2020, Speake 2020, Taylor 2020, Wang 2020) performed genome sequencing (GS) plus phylogenetic analysis.

### Frequency of SARS-CoV-2 attack rates (ARs)

Twenty-three studies reported data on attack rates using RT-PCR ( Table 6). The settings included healthcare (n=3), household (n=8), public transport (n=2), educational settings (n=3). In one study of 84 children in daycare centres during the first few weeks of the pandemic (Desmet 2020), the AR was 0%; similar results were reported in another study of hospital healthcare workers (Basso 2020). The frequency of ARs in the remaining 21 studies ranged from 3.5 to 75% ( Figure 3a). The ARs were highest in weddings (69%), prison (69.5%) and households (75%). Attack rates appeared lower in healthcare settings; two healthcare settings with higher ARs (Ladhani 2020, Ladhani 2020a) included nursing home residents – the definition of SARS-CoV-2 infection in both studies did not include the full constellation of respiratory and non-respiratory symptoms. In sports settings, the AR during matches was between 4.2% and 4.7%.

**Table 6.** Main results of included studies.

Study ID	Type of transmission	Total number of contacts	Cycle threshold	Attack rates and/or secondary attack rates (SAR)	Notes
Abdulrahman 2020	Community	***Eid Alfitr*** Pre-: 71,553; Post-: 76,384 ***Ashura*** Pre-: 97,560; Post-: 118,548	Not reported	**Eid Alfitr** Pre-: 2990 (4.2%); Post-: 4987 (6.7%); p <0.001 **Ashura ** Pre-: 3571 (3.7%); Post-: 7803 (6.6%); p <0.001	The rates of positive tests was significantly greater after religious events
Adamik 2020	Household	Unclear	Not reported	Unclear: 3553 (AR 26.7%)	
Agergaard 2020	Household	PCR: 5 Serology: 5	Not reported	Index case plus 1 family member tested positive- PCR All 5 displayed a serological SARS-CoV-2 N/S IgG response	
Angulo-Bazán 2020	Household	52 households (n=236 people) 4.5±2.5 members per household	Not reported	Serology: Amongst cohabitants, SAR was 53.0% (125 cases): 77.6% of cases were symptomatic	Convenience sampling, no component of temporality, selection bias
Armann 2020	Local Household	2045 in Phase 1 1779 in Phase 2	N/A	Serology: 12/2045 (0.6%) Serology: 12/1779 (0.7%)	
Arnedo-Pena 2020	Household	745	Not reported	11.1% (95% CI 9.0–13.6)	
Baker 2020	Nosocomial	44	Not reported	3/44 (6.8%): 1 of these was also exposed to a household member with COVID-19.	Recall error and bias, report is limited to a single exposure, change in mask policy partway through the exposure period
Baettig 2020	Local	55	Not reported	Serologic attack rates: 2/55 (3.6%)	Serological testing was positive for the 2 contacts 14 days after index case
Bao 2020	Community	57 index cases 1895 exposed	Not reported	SAR was 3.3% at the bathing pool, 20.5% in the colleagues’ cluster and 11.8% in the family cluster.	Delayed detection of the activity trajectory of the primary case, reporting bias, overlap of close contacts
Basso 2020	Nosocomial	60 HCWs - ≥106 unique high-risk contacts	Not reported	Attack rate: 0/60 (0%) Serology: 0/60 (0%)	Delay in diagnosing index case, recall bias
Bays 2020	Nosocomial	421 HCWs	Not reported	8/421 (1.9%)	In all 8 cases, the staff had close contact with the index patients without sufficient PPE. Hospital staff developing ILI symptoms were tested for SARS-CoV-2, regardless of whether they had contact with an index patient
Bi 2020	Local Household Community	1,296	Not reported	98/1286 (7.6%)	
Blaisdell 2020	Community	1,022	Not reported	1.8% of camp attendees (10 staff members and 8 campers)	Travel was assumed to be from home state but intermediate travel might have occurred
Böhmer 2020	Local Household	241	Not reported	75·0% (95% CI 19·0–99·0; three of four people) among members of a household cluster in common isolation, 10·0% (1·2–32·0; two of 20) among household contacts only together until isolation of the patient, and 5·1% (2·6–8·9; 11 of 217) among non-household, high-risk contacts.	
Boscolo-Rizzo 2020	Household	296	Not reported	74/296 (25.0%, 95% CI 20.2–30.3%)	The prevalence of altered sense of smell or taste was by far lower in subjects negative to SARS-CoV-2 compared to both positives (p < 0.001) and non-tested cases (p < 0.001).
Brown 2020	Local	21	Not reported	Serologic attack rate: 2/21 (1%)	Social desirability bias likely
Burke 2020	Household	445	Not reported	0.45% (95% CI = 0.12%–1.6%) among all close contacts, and a symptomatic secondary attack rate of 10.5% (95% CI = 2.9%–31.4%) among household members.	2 persons who were household members of patients with confirmed COVID-19 tested positive for SARS-CoV-2.
Canova 2020	Nosocomial	21	Not reported	0/21 (0%)	
Cariani 2020	Nosocomial	Unclear	33.6 to 38.03	182 out of 1683 (10.8%) tested positive; 27 of whom had close contact with COVID-positive patients	Unclear how many HCWs had close contact; likelihood of recall bias
Charlotte 2020	Community	27	Not reported	19 of 27 (70%) tested positive	High risk of selection bias: The index case- patients were not identified. A majority of patients were not tested for SARS-CoV-2
Chaw 2020	Local Community	1755	Not reported	Close contact: 52/1755 (29.6%) Nonprimary attack rate: 2.9% (95% CI 2.2%– 3.8%)	Potential environmental factors were not accounted for: relative household size, time spent at home with others, air ventilation, and transmission from fomites.
Chen 2020	Aircraft	335	Not reported	16/335 (4.8%)	Recall bias. Did not perform virus isolation and genome sequencing of the virus, which could have provided evidence of whether viral transmission occurred during the flight.
Chen 2020a	Local Household	209	Not reported	0/209 (0%)	
Chen 2020b	Nosocomial	105	Not reported	Serology: 18/105 (17.1%)	
Chen 2020c	Local Community Household Nosocomial	2147	Not reported	110/2147 (5.12%)	
Cheng 2020	Household Nosocomial	2761	Not reported	0.70%	
Chu 2020	Community	50 exposed	Not reported	None for antigen or antibody: 0/50 (0%)	Testing was biased toward contacts who knew the case-patient personally (office co-workers) or provided direct care for the case-patient (HCP).
Chu 2020a	Household	526 exposed	Not reported	48 (9%) (CI 7-12%)	Very high risk of selection bias
Contejean 2020	Nosocomial	1344 exposed	Not reported	373 (28%)	
COVID-19 National Emergency Response Center 2020	Local Household Nosocomial	2370	Not reported	13/2370 (0.6%)	There were 13 individuals who contracted COVID-19 resulting in a secondary attack rate of 0.55% (95% CI 0.31–0.96). There were 119 household contacts, of which 9 individuals developed COVID-19 resulting in a secondary attack rate of 7.56% (95% CI 3.7–14.26).
Danis 2020	Local Household	Chalet: 16 School: 172	Not reported	Attack rate: 75% in chalet Attack rate: 0% in school	Only 73 of 172 school contacts were tested - all tested negative
Dattner 2020	Household	3353	Not reported	Attack rates: 25% in children and 44% adults (45% overall) Serology: 9/714 (1.3%)	
de Brito 2020	Household	24 exposed	Not reported	RT-PCR: 6/7 (86%); Seropositivity: 18/24 (75%)	
Deng 2020		347	Not reported	25/347 (7.2%)	
Desmet 2020	Local	84	38.8	Attack rate: 0/84 (0%)	Ct reported for only one test result
Dimcheff 2020	Community Nosocomial Household	1476	Not reported	Seroprevalence 72/1476: 4.9% (95% CI, 3.8%–6.1%)	
Dong 2020	Household	259	Not reported	53/259 (20.5%)	
Doung-ngern 2020	Local	211 cases plus 839 non-matched controls	Not reported		
Draper 2020	Local Household Nosocomial	445	Not reported	4/445 (0.9%)	None of the 326 aircraft passengers or 4 healthcare workers who were being monitored close contacts became cases.
Dub 2020	Local Household	121	Not reported	Child index case: No positive cases Adult index case: 8/51 (16%) Serology: 6/101 (5.9%)	
Expert Taskforce 2020	Local	Unclear	Not reported	Attack rate 20.4%	Attack rates were highest in 4-person cabins (30.0%; n = 18), followed by 3-person cabins (22.0%; n = 27), 2-person cabins (20.6%; n = 491), and 1-person cabins (8%; n = 6).
Fateh-Moghadam 2020	Community	6690	Not reported	890/6690 (13.3%)	
Firestone 2020	Local	Unclear	Not reported	41 (80%) interviewed patients with primary event-associated COVID-19 reported having close contact with others during their infectious period, with an average of 2.5 close contacts per patient. 36 (75%) of 48 interviewed patients with primary event-associated cases reported having close contact with persons in their household while infectious, and 17 (35%) reported having other (social/workplace) close contacts while infectious.	
Fontanet 2020	Local	661	N/A	Serology: 171/661 (25.9%, 95%CI 22.6-29.4)	
Fontanet 2020a	Local	510	N/A	Serology: 45/510 (8.8%)	
Gan 2020	Local Household Community	Unclear	Not reported	Not reported	Family clusters accounted for 86.9% (914/1 050) of cases, followed by party dinners (1.1%)
Ghinai 2020	Community	Unclear	Not reported	Unclear	
Gong 2020	Household Community	Unclear	Not reported	Unclear	
Gu 2020	Local	14	Not reported	RT-PCR - 3/14 (21.4%) Serology - 2/14 (14.3%)	
Hamner 2020	Local	60	Not reported	Confirmed: 32/60 (53.3%) Probable: 20/60 (33.3%)	
Han 2020	Community	192	Not reported	7/192 (3.7%)	
Heavey 2020	Local	1155	Not reported	0/1155 (0%)	
Helsingen 2020	Local	Training arm: 1,896 Nontraining arm: 1,868	Not reported	11/1896 (0.8%) vs 27/1868 (2.4%); P=0.001	
Hendrix 2020	Local	139 exposed	Not reported	0%	Six close contacts of stylists A and B outside of salon A were identified: four of stylist A and two of stylist B. All four of stylist A’s contacts later developed symptoms and had positive PCR test results for SARS-CoV-2. These contacts were stylist A’s cohabitating husband and her daughter, son-in-law, and their roommate, all of whom lived together in another household. None of stylist B’s contacts became symptomatic.
Hirschman 2020	Household Community	58	Not reported	27/58 (47%)	
Hobbs 2020	Local Household Community	397	Not reported	Not reported	
Hoehl 2020	Local Community	825 children and 372 staff: 7,366 buccal mucosa swabs and 5,907 anal swabs	Not reported	0% viral shedding in children; 2/372 (0.5%) shedding for staff. No inapparent transmissions were observed	Study was conducted in the summer of 2020, when activity of other respiratory pathogens was also low
Hong 2020	Household	431 tests	Not reported	0/13 (0%)	Index cases had lived with their family members without personal protections for a total of 258 person-days.
Hu 2020	Household Community	15648	Not reported	471/15648 (3%)	
Hua 2020	Household	835	Not reported	151/835 (18.1%)	
Huang 2020	Household Community	22	Not reported	7/22 (31.8%)	
Huang 2020a	Local Household Community Nosocomial	3795	Not reported	32/3795 (0.84%)	
Islam 2020	Household Local Community Nosocomial	391	Not reported	The overall secondary clinical attack rate was 4.08 (95% CI 1.95–6.20)	
Jia 2020	Household	Unclear	Not reported	Attack rate 44/583 (7.6%)	
Jiang 2020	Household Community	300	Not reported	6/300 (2%)	
Jing 2020	Household	Unclear	Not reported	Household contacts 13·2% Non-household contacts 2·4%	The risk of household infection was significantly higher in the older age group (≥60 years)
Jing 2020a	Household Community	Unclear	Not reported	Close contacts 17.1% to 19% Family members 46.1% to 49.6%	
Jones 2020	Local	128	Not reported	6/128 (4.7%)	
Kang 2020	Local	5517	Not reported	96/5517 (1.7%)	
Kant 2020	Local Community Nosocomial	Not reported	Not reported	Not reported	No details on number of contacts for index case
Kawasuji 2020	Nosocomial	105	Not reported	14/105 (1.33%)	
Khanh 2020	Community	217	Not reported	16/217 (7.4%)	
Kim 2020	Household	207	17.7 to 30	1/207 (0.5%)	
Kim 2020a	Household Community	4	18.7 to 32.1	N/A	
Kim 2020b	Nosocomial	3,091 respiratory samples from 2,924 individuals	Not reported	3/290 (1%)	
Kumar 2020	Community	822	Not reported	144/822 17.5%)	Spread of infection within the state was significantly higher from symptomatic cases, p=0.02
Kuwelker 2020	Household	179	N/A	45%	The elderly (>60 years old) had a significantly higher attack rate (72%) than adults< 60years old (46%, p=0·045)
Kwok 2020	Local Household	206	Not reported	24/206 (11.7%)	
Ladhani 2020	Nosocomial	254	Not reported	Unclear: 53/254 (21%) tested positive.	Staff working across different care homes (14/27, 52%) had a 3.0-fold (95% CI, 1.9–4.8; P<0.001) higher risk of SARS-CoV-2 positivity than staff working in single care homes (39/227, 17%).
Ladhani 2020a	Nosocomial	Residents: 264 Staff members: 254	Not specified	Unclear: 105/264 (53%) residents tested positive	Infectious virus recovery in asymptomatic staff and residents emphasises their likely importance as silent reservoirs and transmitters of infection and explains the failure of infection control measures which have been largely based on identification of symptomatic individuals.
Laws 2020	Household	188	Not reported	55/188 (29.3%)	
Laxminarayan 2020	Local Household Community	575,071	Not reported	10.7% (10.5 to 10.9%) for high-risk contacts 4.7% (4.6 to 4.8%) for low-risk contacts 79.3% (52.9 to 97.0%) for high-risk travel exposure	
Lee 2020	Household	12	Not reported	0/12 (0%)	
Lee 2020a	Household	23	Not reported	1/23 (4.4%)	
Lewis 2020	Household	188	Not reported	RT-PCR: 55/188 (29%) Serology: 8/52 (15%)	
Li 2020	Household	5	19.66 to 26.16	4/5 (80%)	
Li 2020a	Household Nosocomial	7	Not reported	7/7 (100%)	During January 14–22, the authors report that index patient had close contact with 7 persons
Li 2020b	Household	14	Not reported	14/14 (100%)	
Li 2020c	Household	Unclear	Not reported	Unclear	In COFs, the transmission rates of respiratory droplets in secondary and non-infected patients were 11.9 % and 66.7 %, respectively, while the transmission rates of respiratory droplets with close contacts were 88.1 % and 33.3 %, respectively. In SOFs, the proportion of respiratory droplet and respiratory droplet transmission with close contacts was 40 % and 60 %, respectively
Li 2020d	Household	392	Not reported	64/392 (16.3%)	
Liu 2020	Household	7	Not reported	4/7 (57.1%)	
Liu 2020a	Nosocomial	30	Not reported	N/A	
Liu 2020b	Household Community Nosocomial	11580	Not reported	515/11580 (4.4%)	
Liu 2020c	Unclear	1150	Not reported	47/1150 (4.1%)	The 16 confirmed cases who had previously been asymptomatic accounted for 236 close contacts, with a second attack rate of 9.7%, while the remaining 131 asymptomatic carriers accounted for 914 close contacts, with a second attack rate of 2.6% (p<0.001)
López 2020	Local Household	285	Not reported	Facility SAR: 22/101 (21.8%) Overall SAR: 38/184 (20.7%)	Variation in hygiene procedures across 3 facilities. **Facility A** required daily temperature and symptom screening for the 12 staff members and children and more frequent cleaning and disinfection; staff members were required to wear masks. **Facility B:** temperatures of the five staff members and children were checked daily, and more frequent cleaning was conducted; only staff members were required to wear masks. **Facility C:** 84 staff members and children check their temperature and monitor their symptoms daily; masks were not required for staff members or children.
Lopez Bernal 2020	Household Community	472	Not reported	37% (95% CI 31–43%)	
Lucey 2020	Nosocomial	Not specified	N/A	Not reported	
Luo 2020	Community	243	Not reported	12/243 (4.9%)	No viral genetic sequence data were available from these cases to prove linkage; and some of the secondary and tertiary cases could have been exposed to unknown infections, especially asymptomatic ones, before or after the bus trips.
Luo 2020a	Household Community Nosocomial	3410	Not reported	127/3410 (3.7%)	
Lyngse 2020	Household	2226	Not reported	371/2226 (16.7%)	
Ma 2020	Unclear	1665	Not reported	10/1/1665 (0.6%)	Only close contacts who fell ill were tested (n=10)
Macartney 2020	Local	633	Not reported	18/633 (1.2%) Serologic attack rates: 8/171 (4.8%)	
Malheiro 2020	Household	1627	Not reported	Overall AR 154/1627 (9.5%)	
Maltezou 2020	Household	Unclear	<25 (28.1%) 25–30 (26.8%) >30 (45.1%)	Median attack rate 40% (range: 11.1%–100%) per family.	
Maltezou 2020a	Household	Unclear	Not reported	Median attack rate: 60% (range: 33.4%-100%)	Adults were more likely to develop a severe clinical course compared to children (8.8% versus 0%, p-value=0.021)
Mao 2020	Household Local	Unclear	Not reported	6.10%	Average attack rate was 8.54% (1.02–100%)
Martinez-Fierro 2020	Unclear	81	Not reported	34/81 (42%) Serologic attack rates: 13/87 (14.9%)	16% of contact showed positive serology after >2 weeks
Mponponsuo 2020	Nosocomial	38	N/A	0/38 (0%)	
Ng 2020	Household Local Community	13026	Not reported	188/7770 (2.4%) Household: 5·9% Work contacts: 1.3% Social contacts: 1.3% Serology: 44/1150 (3.8%)	Serology results were positive for 29 (5·5%) of 524 household contacts, six (2·9%) of 207 work contacts, and nine (2·1%) of 419 social contacts.
Ning 2020	Household Local Community	Unclear	Not reported	Imported cases: 69/3435 (0.8%) Local cases: 31/3666 (2.0%)	
Njuguna 2020	Local	98	Not reported	Attack rate 57% to 82%	
Ogawa 2020	Nosocomial	30 PCR/serology	33.53 to 36.83	0/15 (0%) for both PCR and serology	
Paireau 2020	Household Local Nosocomial	6028	Not reported	248/6028 (4.1%)	Family contacts, index case was 60–74, or older than 75 years old were significantly associated with increased odds of transmission. The proportion of nosocomial transmission was significantly higher than in contact tracing (14% vs 3%, p<0.001)
Park 2020	Local Household Community	328	17.7 to 35	22/328 (6.7%)	
Park 2020a	Household Non-household	59,073	Not reported	Household contacts: 11.8% (95% CI 11.2%– 12.4%) Non-household contacts: 1.9% (95% CI 1.8%–2.0%)	
Park 2020b	Local Household	441	Not reported	Attack rate 43.5% (95% CI 36.9%–50.4%) Secondary attack rate 16.2% (95% CI 11.6%– 22.0%)	
Passarelli 2020	Nosocomial	6	Not reported	2/6 (33.3%)	
Patel 2020	Household	185	Not reported	79/185 (43%)	Contacts not reported as tested
Pavli 2020	Aircraft	891	Not reported	5/891 (0.6%)	
Phiriyasart 2020	Household	471	Not reported	27/471 (5.7%)	
Poletti 2020	Unclear	2484	Not reported	2824/5484 (51.5%)	
Pung 2020	Local Community	425	Not reported	36/425 (8.5%)	
Pung 2020a	Household	Unclear	Not reported	43/875 (4.9%)	
Qian 2020	Local Household Community	Not reported	Not reported	Not reported	Home‐based outbreaks were the dominant category (254 of 318 outbreaks; 79.9%), followed by transport‐based outbreaks (108; 34.0%)
Ravindran 2020	Local	Not reported	Not reported	Attack rate 61% to 77%	All attendees participated in activities resulting in potential exposure, such as shaking hands, kissing, dancing, sharing drinks and sharing shisha (smoking water pipes).
Razvi 2020	Nosocomial	2521	Not reported	Serologic attack rate 19.4%	
Rosenberg 2020	Household	498	Not reported	286/498 (57%)	
Roxby 2020	Nosocomial	142	Not reported	Attack rate in 1st round: 5/142 (3.5%)	One additional positive test result was reported for an asymptomatic resident who had negative test results on the first round.
Sang 2020	Household	6	Not reported	4/6 (66.7%)	
Schumacher 2020	Local	Quarantine phase: 757 tests Match phase: 1167 tests	Unclear	Quarantine phase AR: 3.6% Match phase AR: 4.2% Serology: 1.1%	
Schwierzeck 2020	Nosocomial	48	16.03 to 32.98	9/48 (18.8%)	Ct values of symptomatic cases were significantly lower compared to asymptomatic cases 22.55 vs 29.94, p<0.007 (approximately 200-fold higher viral load)
Shah 2020	Household	386	Not reported	34/386 (8.8%)	
Shen 2020	Household Community	480	Not reported	Close contact: 2/7 (29%) Casual contact: 3/473 (0.6%)	
Sikkema 2020	Nosocomial	1796	Not specified. WGS for Ct <32	Attack rate 96/1796 (5%)	46 (92%) of 50 sequences from health-care workers in the study were grouped in three clusters. Ten (100%) of 10 sequences from patients in the study grouped into the same three clusters:
Son 2020	Household	3223	Not reported	8.2% (95% CI, 4.7 to 12.9)	
Song 2020	Household	20	Not reported	16/20 (80%)	
Speake 2020	Aircraft	111	Not reported	11/111 (9.9%)	
Stein-Zamir 2020	Local	1312	Not reported	Attack rate 178/1312 (13.6%)	
Sugano 2020	Local	72	Not reported	23/72 (31.9%)	
Sun 2020	Household	Unclear	Not reported	34.43%	
Taylor 2020	Nosocomial	600	Not reported	Resident attack rate: 137/259 (52.9%) 1st round HCW Attack rate: 114/341 (33.4%)	
Teherani 2020	Household	144	Not reported	67/144 (46.5%)	Of the total number of household contacts, at least 29 (20%) had known SARS-CoV2 testing. Child-to-adult transmission was suspected in 7/67 cases (10.5%).
Thangaraj 2020	Community	26	Not reported	17/26 (65.4%)	
Torres 2020	Community	1244	N/A	Overall serologic attack rate: 139/1244 (11.2%)	
Tshokey 2020	Local Community	1618	Not reported	14/1618 (0.9%)	SAR: High-risk contacts was 9.0% (7/75), and that among the primary contacts was 0.6% (7/1,095), and none (0/448) among the secondary contacts.
van der Hoek 2020	Household	174	25.1 to 35.1	47/174 (27%) Serology on day 3 - family members: 43/148 (29.1%)	
Wang 2020	Nosocomial Household	43	Not reported	10/43 (23.3%)	
Wang 2020a	Household	155	Not reported	47/155 (30%)	
Wang 2020b	Household	335	Not reported	77/335 (23%)	
Wee 2020	Nosocomial	298	Not reported	1/298 (0.3%)	
Wendt 2020	Nosocomial	254	Not reported	0/254 (0%) Serologic attack rates 0/23 (0%)	
Wolf 2020	Household	4	Not reported	3/4 (75%)	7-month old female who was breastfed, was asymptomatic throughout the observation period and never developed fevers or any other symptoms, despite continuous exposure to her parents and siblings. She remained SARS-CoV-2 PCR-negative in repeat testing of pharyngeal swab and stool specimens over the entire observation period.
Wong 2020	Nosocomial	76 tests were performed on 52 contacts	Not reported	0/52 (0%)	Findings suggest that SARS-CoV-2 is not spread by an airborne route. Ct value for throat and tracheal aspirate of index case were 22.8 and 26.1 respectively
Wood 2020	Household	Not reporred	Not reported	Not reported	
Wu 2020	Household Local Community	2994	Not reported	71/2994 (2.4%)	
Wu 2020a	Household	148	Not reported	48/148 (32.4%)	
Xie 2020	Household	56	Not reported	0/56 (0%)	
Xin 2020	Household	187	Not reported	19/187 (17.9%)	
Yang 2020	Household Local	1296	Not reported	0/1296 (0%) Serologic attack rates: 0/20 (0%)	Viral culture of 4 specimens with Ct <30 were negative
Yau 2020	Nosocomial	330	Not reported	22/330 (6.7%)	
Ye 2020	Local Community	1293	Not reported	39/1,293 (3.02%)	
Yoon 2020	Local	190	N/A	0/190 (0%)	
Yousaf 2020	Household	198	Not reported	47/198 (23.7%)	
Yu 2020	Household	1587	Not reported	150/1587 (9.5%)	
Yung 2020	Household	213	Not reported	Attack rate 6.1%	
Zhang 2020	Aircraft	4492	Not reported	Attack rate 161/4492 (3.6%)	The authors report attack rate of 0.14% based on 94 flights (n=14 505); however, only 4492 people were screened
Zhang 2020a	Household Local Community	369	Not reported	12/369 (3.3%, 95% CI 1.9%–5.6%)	
Zhang 2020b	Household	10	Not reported	0/10 (0%) Serologic attack rates: 0/10 (0%)	
Zhang 2020c	Local Household	93	Not reported	5/93 (5.4%)	
Zhang 2020d	Local	8437	Not reported	25/8437 (0.3%)	
Zhuang 2020	Household Community	8363	Not reported	239/8363 (2.9%)	

**Figure 3a.**
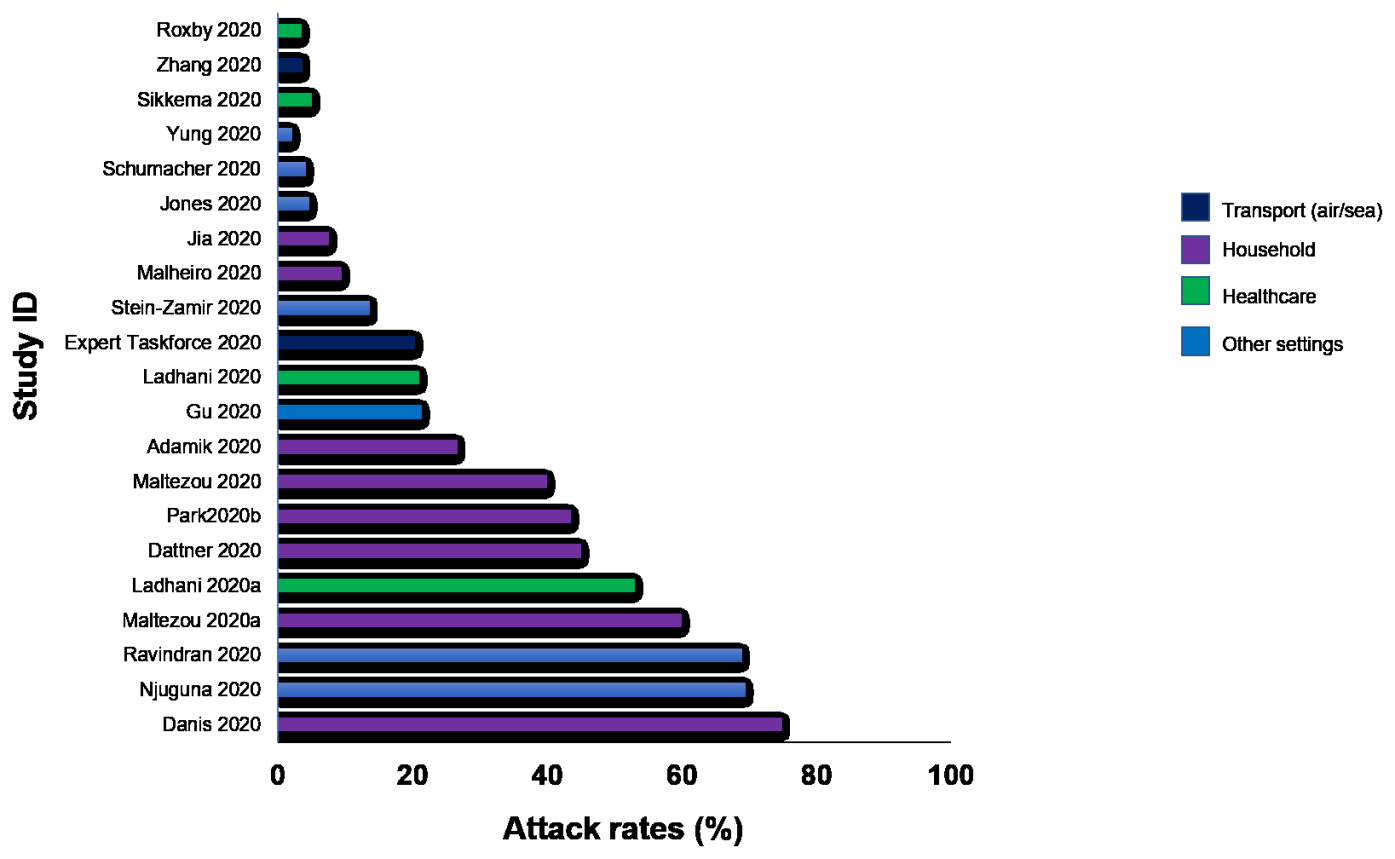
Primary attack rates of SARS-CoV-2 in close contacts (PCR).

Twenty-nine studies reported data on ARs using serology ( Table 6). The settings included educational (n=4), households (n=4) and healthcare (n=3). In eight studies, the frequency of attack was 0%. The frequency of attacks in the remaining 21 studies ranged from 0.7% to 75% ( Figure 3b). The frequency of attacks was highest in households but lower in educational settings - especially daycare centres.

**Figure 3b.**
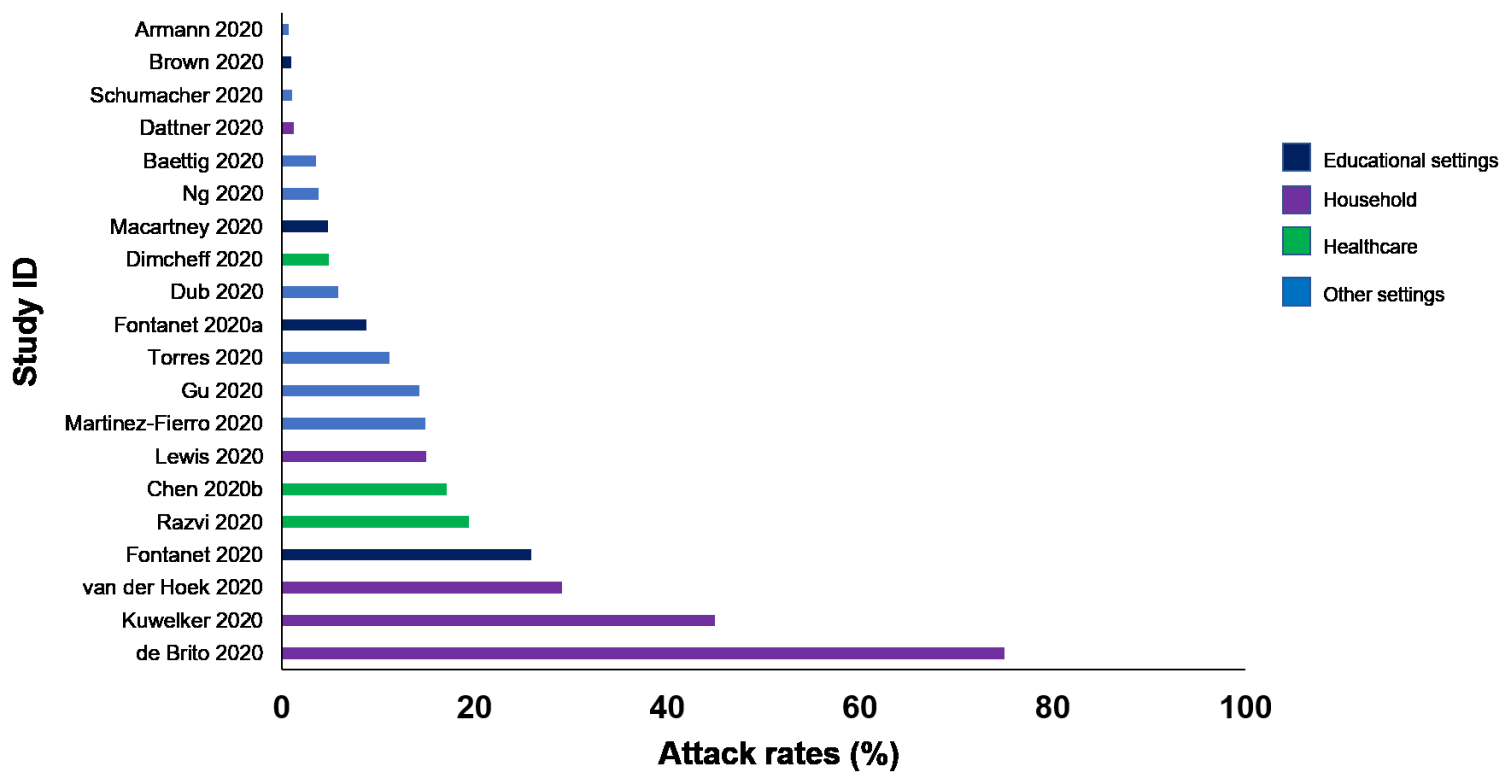
Primary attack rates of SARS-CoV-2 in close contacts (serology).

### Frequency of SARS-CoV-2 secondary ARs

**Figure 4.**
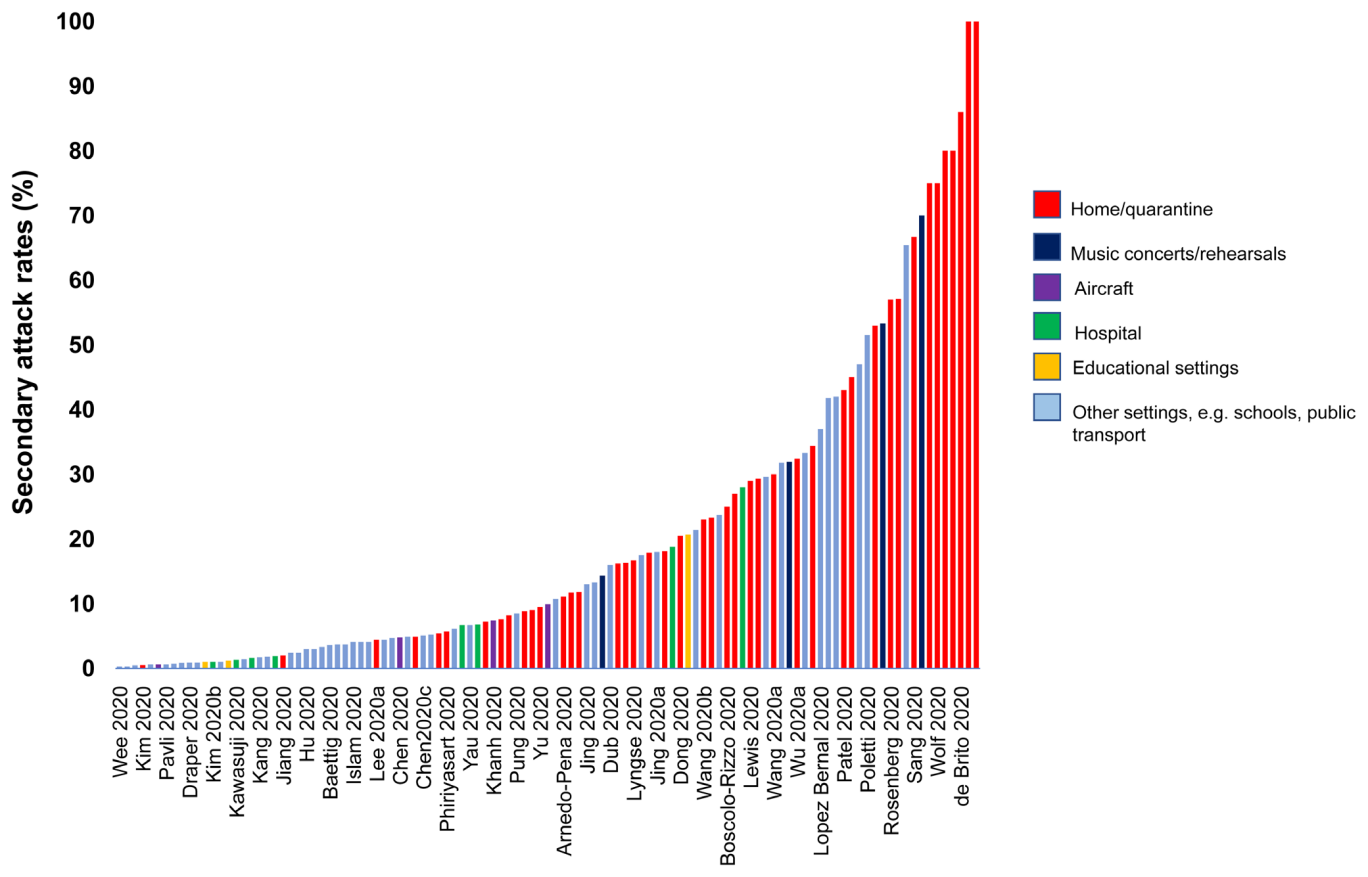
Frequency of secondary attack rates of SARS-CoV-2 with Close Contacts.

Overall, 126 studies (73.7%) reported data on secondary ARs ( Table 6). The studies reported the rates based on RT-PCR tests, except for one study (Angulo-Bazán 2020) that used serology. In 16 of these studies, the SAR was 0%. The secondary ARs in the remaining 110 studies ranged from 0.3 to 100% (see Figure 4). The highest frequencies of secondary ARs (75–100%) occurred in household or quarantine settings; similar findings were observed when studies with higher reporting quality were examined (57–75%). In the three studies of index or primary cases with recurrent infections, there was no positive case amongst the 1518 close contacts across the studies.

### Risk of infection

Forty-six studies (26.9%) reported results on the risk of infection ( Table 7). One study of airline passengers (Khanh 2020) showed that seating proximity was significantly associated with the risk of contracting SARS-CoV-2 (RR 7.3, 95% CI 1.2–46.2); a second study (Speake 2020) reported that not sitting by the window was associated with a significantly increased risk of infection (RR 5.2; 95% CI 1.6–16.4; p<0.007)). The results of five studies (Chen 2020b, Doung-ngern 2020, Hobbs 2020, Wang 2020b, Wu 2020) showed that use of face covering during close contact with infected cases was associated with significantly lower risks of infection compared with no face covering; findings from one of these studies (Doung-ngern 2020) showed that wearing masks all the time during contact was not significantly different from wearing masks sometimes. The result of one study (Rosenberg 2020) showed that the incidence of infection significantly increased with age (p<0.0001), while those from another study (Poletti 2020) showed that being 70 years or older was associated with a significantly increased risk of SARS-CoV-2-related death (p<0.001), while another study (Zhang 2020a) reported that elderly close contacts (≥60 years) had a higher SAR compared with younger age groups. Findings from five studies (Bi 2020, Hu 2020a, Islam 2020, Luo 2020a, Wu 2020, Zhang 2020a) showed that household contact settings had significantly higher risks of infection compared with other types of contact settings, e.g., social, healthcare, workplace and public transport. One study (Lewis 2020) showed that the risk of infection was significantly increased amongst household contacts who were immunocompromised (OR 15.9, 95% CI 2.4–106.9). Finally, three studies (Bi 2020a, Wu 2020, Zhang 2020a) showed that the more frequent contacts with an index case was significantly associated with an increased risk of infection.

**Table 7.** Risk of infection in close contacts.

Study ID	Type of transmission	Risk of infection
Abdulrahman 2020	Community	**Eid Alfitr:** Pre-: 2990 (4.2%); Post-: 4987 (6.7%); p <0.001; **Ashura:** Pre-: 3571 (3.7%); Post-: 7803 (6.6%); p <0.001
Arnedo-Pena 2020	Household	The health profession of index case was a significant protective factor (p<0.007). Older age of secondary cases, two household members, and higher age of index case were significantly associated with elevated risk of infection: p<0.001 in each case
Bi 2020	Local Household Community	Household contact (OR 6·3; 95% CI 1·5–26·3) and travelling together (OR 7·1; 1·4–34·9) were significantly associated with infection. Reporting contact that occurred often was also associated with increased risk of infection compared with moderate-frequency contact (OR 8·8; 95% CI 2·6–30·1)
Chen 2020b	Nosocomial	In multivariate analysis, there existed higher risk of seroconversion for close contacts with patient 2 (OR, 6.605, 95% CI, 1.123, 38.830) and doctors exposed to their patient (OR, 346.837, 95% CI 8.924, 13479.434), while the lower risk of seroconversion was closely related to direct contact with COVID-19 patients wearing face mask (OR, 0.127, 95% CI 0.017, 0.968).
Chen 2020c	Local Community Household Nosocomial	Infection rate is highest when living with the case (13.26%), followed by taking the same means of transportation (11.91%). After removing the influence factors of the "super spreader" incident, the infection rate of vehicle contact dropped to 1.80%. The infection rate (7.18%) of entertainment activities such as gatherings, meeting guests, and playing cards was also relatively high, as was short-term face-to-face unprotected conversations or doing errands (6.02%). There was a statistically significant difference in the infection rate among the four categories of life contact, transportation contact, medical contact, and other contact (p<0.005). participation in Buddhist gatherings caused transmission. A total of 28 people were diagnosed as confirmed cases of new coronavirus pneumonia, 4 were asymptomatic infections, and the infection rate of close contacts reached 32.99% (32/97), which was much higher than the average infection rate (6.15). %), the difference is statistically significant (p<0.005).
Cheng 2020	Household Nosocomial	The overall secondary clinical attack rate was 0.7% (95% CI, 0.4%-1.0%). The attack rate was higher among the 1818 contacts whose exposure to index cases started within 5 days of symptom onset (1.0% [95% CI, 0.6%-1.6%]) compared with those who were exposed later (0 cases from 852 contacts; 95% CI, 0%-0.4%). The 299 contacts with exclusive presymptomatic exposures were also at risk (attack rate, 0.7% [95% CI, 0.2%-2.4%]). The attack rate was higher among household (4.6% [95% CI, 2.3%-9.3%]) and nonhousehold (5.3% [95% CI, 2.1%-12.8%]) family contacts than that in health care or other settings. The attack rates were higher among those aged 40 to 59 years (1.1% [95% CI, 0.6%-2.1%]) and those aged 60 years and older (0.9% [95% CI, 0.3%-2.6%]).
Chu 2020a	Household	Five (10%) of 48 secondary cases compared with 130 (33%) of 398 non-case household contacts reported potential community exposures: unadjusted OR 0.24 (95%CI 0.09 to 0.62), p=0.003
Dattner 2020	Household	PCR: 44% of adults were infected compared to 25% of the children (n=3353: 1809 children and 1544 adults) Serology: 34% of these children and 48% of the adults tested serologically positive (n=705: 417 children and 288 adults
Dimcheff 2020	Community Nosocomial Household	HCWs exposed to a known COVID-19 case outside work had a significantly higher seroprevalence at 14.8% (23 of 155) compared to those who did not 3.7% (48 of 1,296; OR, 4.53; 95% CI, 2.67–7.68; P < 0.0001)
Doung-ngern 2020	Local	Wearing masks all the time during contact was independently associated with lower risk of COVID-19 infection compared to not wearing masks (aOR 0.23, 95% CI 0.09–45 0.60), while wearing masks sometimes during contact was not (aOR 0.87, 95% CI 0.41–1.84). Maintaining at least 1m distance from a COVID patient (aOR 0.15, 95% CI 0.04–0.63) and duration of close contact ≤15 minutes versus longer (aOR 0.24, 95% CI 0.07–0.90) were significantly associated with lower risk of infection transmission
Fateh-Moghadam 2020	Community	Workplace exposure was associated with higher risk of becoming a case than cohabi_x0019_ng with a case or having a non-cohabiting family member or friend who was a case. The greatest risk of transmission to contacts was found for the 14 cases <15 years of age (22.4%); 8 of the 14, who ranged in age from <1 to 11 years) infected 11 of 49 contacts.
Fontanet 2020a	Local	No significant difference in attack rates across primary school pupils, teachers, non-teaching staff, parents, and relatives, respectively (p=0.29).
Helsingen 2020	Local	11 individuals in the training arm (0.8% of those tested) and 27 in the non-training arm (2.4% of those those tested) tested positive for SARS- CoV-2 antibodies (p=0.001)
Hobbs 2020	Local Household Community	Case-patients were significantly more likely to have had close contact with a person with known COVID-19 than control participants (aOR = 3.2, 95% CI = 2.0–5.0) Case-patients were significantly more likely to have attended gatherings with persons outside their household, including social functions (aOR = 2.4, 95% CI = 1.1–5.5), activities with children (aOR = 3.3, 95% CI = 1.3–8.4), or to have had visitors at home (aOR = 1.9, 95% CI = 1.2–2.9) during the 14 days before the SARS-CoV-2 test. Parents of 64% of case-patients and 76% of control participants reported that their child and all staff members wore masks inside the facility (aOR = 0.4, 95% CI = 0.2–0.8).
Hu 2020	Household Community	Household contacts were associated with a significantly larger risk of SARS-CoV-2 infection than other types of contact (P<0.001). The transmission risk in the first generation was significantly higher than the later generations (p<0.001), possibly due to improved case isolation and contacts quarantine that deplete the number of susceptible individuals in the cluster.
Hua 2020	Household	Incidence of infection in child close contacts was significantly lower than that in adult contacts: 13.2% vs 21.2%, p=0.004
Islam 2020	Household Local Community Nosocomial	The secondary attack rate among household contacts was at the highest risk of attack (13.04%, 95% CI 9.67-16.41) followed by funeral ceremonies (8.33%, 95% CI 3.99-12.66) and family contacts (6.52%, 95% CI 4.02-9.02). The attack rate was higher in age groups 50–59 (10.89%, 95% CI 7.05-14.66) and 60–69 (9.09%, 95% CI 5.08-13.09)
Kawasuji 2020	Nosocomial	Among symptomatic patients (n =18), the estimated viral load at onset was higher in the index than in the non-index patients (median [95% confidence interval]: 6.6 [5.2–8.2] vs. 3.1 [1.5–4.8]. In adult (symptomatic and asymptomatic) patients (n = 21), median viral load at the initial sample collection was significantly higher in the index than in the non-index patients (p = 0.02)
Khanh 2020	Community	Seating proximity was strongly associated with increased infection risk (RR 7.3, 95% CI 1.2–46.2).
Laws 2020	Household	There were no significant differences in secondary infection rates between adult and pediatric contacts among all households (OR: 1.11; 95% CI: 0.56 to 2.21) or among households with children (OR: 0.99; 95% CI: 0.51 to 1.90).
Laxminarayan 2020	Local Household Community	Secondary attack rate estimates ranged from 1.2% (0.0 to 5.1%) in health care settings to 2.6% (1.6 to 3.9%) in the community and 9.0% (7.5 to 10.5%) in the household.
Lewis 2020	Household	Household contacts to COVID-19 patients with immunocompromised conditions and household contacts who themselves had diabetes mellitus had increased odds of infection with ORs 15.9 (95% CI, 2.4–106.9) and 7.1 (95% CI: 1.2–42.5). Household contacts of a male primary patient were more likely to have secondary infection than those of a female primary patient (SIR, 36% vs 18%; OR, 2.4; 95% CI, 1.1–5.3).
Li 2020d	Household	The secondary attack rate to children (aged <18 years) was 4% compared with 20.5% for adult members (odds ratio [OR], .18; 95% confidence interval [CI], .06–.54; P = .002). The secondary attack rate to the contacts in the household with index patients quarantined at home immediately since onset of symptoms was 0% compared with 18.3% for the contacts in the households without index patients quarantined during the period between initiation of symptoms and hospitalization (OR, 0; 95% CI, .00–.00; p=0.000). The secondary transmission rate for individuals who were spouses of index cases was 27.8% compared with 17.3% for other members in the households (OR, 2.27; 95% CI, 1.22–4.22; p=0.010).
Liu 2020b	Household Community Nosocomial	Compared to young adults aged 20–29 years, the infected risk was higher in children (RR: 2.59, 95%CI: 1.79–3.76), and old people aged 60–69 years (RR: 5.29, 95%CI: 3.76–7.46). People having close relationship with index cases encountered higher infected risk (RR for spouse: 20.68, 95%CI: 14.28–29.95; RR for non-spouse family members: 9.55, 95%CI: 6.73–13.55; RR for close relatives: 5.90, 95%CI: 4.06–8.59). Moreover, contacts exposed to index case in symptomatic period (RR: 2.15, 95%CI: 1.67–2.79), with critically severe symptoms (RR: 1.61, 95%CI: 1.00–2.57)
Lopez Bernal 2020	Household Community	Secondary attack rates were highest where the primary case was aged <18 years with a significantly higher odds of secondary infection (OR 61, 95% CI 3.3-1133). Where the primary case was admitted to hospital there was a significantly lower odds of secondary infection in the household (OR 0.5, 95% CI 0.2-0.8). Secondary attack rates were lower in larger households.
Luo 2020a	Household Community Nosocomial	Household contacts had a significantly higher risk for secondary infection than did persons who were exposed in health care settings (OR, 0.09, 95%CI 0.04 to 0.20) or those who were exposed on public transportation (OR, 0.01, 95%CI, 0.00 to 0.08).
Macartney 2020	Local	The rate of staff member to child transmission was lower (1·5%) than staff to staff transmission (4·4%).
Malheiro 2020	Household	Among the intervention cohort,16 of 132 closecontacts tested positive during the follow-up period (attack rate:12.1%, 95% confidence interval [CI]: 7.1-18.9). In the control cohort,138 of 1495 participants tested positive (attack rate: 9.2%, 95% CI:7.8-10.8)
Park 2020a	Household Non-household	With index patients 30–39 years of age as reference, detection of COVID-19 contacts was significantly higher for index patients >40 years of age in nonhousehold settings.
Phiriyasart 2020	Household	Locally religious and household contacts of confirmed cases had significantly higher risks of SARS-CoV-2 infection than other community members.
Poletti 2020	Unclear	Individuals younger than 70 years were at a significantly lower risk of death after infection than older patients (p<0.001). The risk of death was 62% lower (95% CI: 31–80%; p<0.001) during the second phase of the epidemic.
Razvi 2020	Nosocomial	HCWs in patient facing roles had a significantly higher frequency of positive COVID-19 antibody tests (295/1302 [22.7%]) than those in non- patient facing roles (88/669 [13.2%]), p<0.0001)
Rosenberg 2020	Household	Prevalence significantly increased with age, ranging from 23% among those aged <5 years to 68% among those 65 years or older (p<0.0001)
Speake 2020	Aircraft	The risk for secondary infections among passengers seated in the mid cabin was significantly greater than for those seated in the aft cabin (p<0.005). The SAR among mid-cabin passengers in window seats was significantly greater than among those not in window seats (RR 5.2; 95% CI 1.6–16.4; p<0.007).
Sun 2020	Household	The family recurrence rate of spouses who introduced cases from the family was 63.87%, which was higher than the recurrence rate of children (30.53%), parents (28.37%) and other family members (20.93%), and the difference was statistically significant ( P <0.001) .
Torres 2020	Community	Antibody positivity rates were 9.9% (95%CI: 8.2-11.8) for 1,009 students and 16.6% (95%CI: 12.1-21.9) for 235 staff. Among students, positivity was significantly associated with history of contact with a confirmed case (p<0.0001). The greater the number of contacts, the greater the probability that a child was antibody positive (p=0.05).
van der Hoek 2020	Household	In families of a confirmed COVID-19 patient, children between 1 and 11 years were less often positive in PCR and serology than older children and adults.
Wang 2020b	Household	Face mask use by the primary case and family contacts before the primary case developed symptoms was 79% effective in reducing transmission (OR=0.21, 95% CI 0.06 to 0.79). Daily use of chlorine or ethanol based disinfectant in households was 77% effective (OR=0.23, 95% CI 0.07 to 0.84). Wearing a mask after illness onset of the primary case was not significantly protective. The risk of household transmission was 18 times higher with frequent daily close contact with the primary case (OR=18.26, 95% CI 3.93 to 84.79), and four times higher if the primary case had diarrhoea (OR=4.10, 95% CI 1.08 to 15.60). Household crowding was not significant.
Wood 2020	Household	Households without children had a significantly lower rate of COVID-19: HR per child 0.89; 95% CI 0.84–0.95. Households with childen had higher rates of COVID-19 tests (9.2% vs 6.1%) Compared to those in households without children, the risk of COVID-19 requiring hospitalisation was lower in those with one child and lower still in those with two or more children: HR 0.72 per child (95% CI 0.60-0.85, p<0.001); adjusted for age - HR 0.83 per child (95% CI 0.70-0.99)
Wu 2020	Household Local Community	Contacts living in the same household as the index case had significantly higher risk of infection vs those who had only had brief contact with the index case: RR 41.7 (17.7–98.5), p<0.001). Contacts who had visited, or had contact with the index case in a medical institution had significantly higher risk of acquiring infection vs brief contact with the index case: RR 3.6 (1.42–8.98), p=0.004. Family members who had contact with an index case had significantly higher risk of infection vs healthcare providers or other patients who had been exposed to an index case: RR 31.6 (7.69–130.01), p<0.001. Those who had contact with the index case through work, through study, or in a place of entertainment had a significantly higher risk of infection vs those who had contact with the index case in a medical institution: RR 6.7 (1.34–33.25), p=0.01. Those who had contact with the index case in or near his/her home had a significantly higher risk of infection vs those who had contact with the index case in a medical institution: RR 17.3 (4.20–70.77), p<0.001. The incidence rate among those who wore face masks was significantly lower than that among those who did not use protective measures (0.3% vs. 4.7%, respectively, p<0.001). The incidence rate of contacts with data collected by field investigation was significantly higher than that of contacts with data collected by big data (5.35% versus 0.07%, p<0.001).
Wu 2020a	Household	Contacts with >72 hours of exposure (SIR, 41.7%; [95% CI: 26.8%–58.3%]) had a higher SIR compared with those without (SIR, 23.2%; [95% CI: 11.4%–41.5%]). One household-level factor was significantly associated with SIR: household members without protective measures after illness onset of the index patient (odds ratio [OR], 4.43; [95% CI: 1.37–14.34]).
Xin 2020	Household	Increasing risk of infection among household contacts with female index patients (adjusted hazard ratio [aHR] = 3.84, 95% CI = 1.07–13.78), critical disease index patients (aHR = 7.58, 95% CI = 1.66–34.66), effective contact duration with index patients > 2 days (aHR = 4.21, 95% CI = 1.29–13.73), and effective contact duration > 11 days (aHR = 17.88, 95% CI = 3.26–98.01)
Yu 2020	Household	Family members, colleagues/classmates/travel companions, and doctors-patients accounted for 88.1% (1398), 10.7% (170), and 0.3% (5), respectively. Following this order, the infection rate was 10.2%, 1.8% and 40.0%, respectively.
Yung 2020	Household	Young children <5 years old were at lowest risk of infection (1.3%). Children were most likely to be infected if the household index case was the mother.
Zhang 2020a	Household Local Community	SAR among household contacts was 16.1% vs 1.1% for social contacts, and 0% for workplace contacts. Older close contacts had the highest SAR compared with other age groups; 8.0% in persons >60 years of age compared with 1.4%–5.6% in persons <60 years of age. Close contacts that lived with an index case-patient had 12 times the risk for infection and those who had frequent contact with an index case- patient, >5 contacts during 2 days before the index case was confirmed, had 29 times the risk for infection.
Zhuang 2020	Household Community	The main sources of secondary infection were family exposure (74.5%, 178 cases), transportation exposure accounted for 8.4% (20 cases), friend/colleague meal exposure accounted for 5.9% (14 cases). Shopping malls, markets, pharmacies and other public place exposure accounted for 5.0% (12 cases), workplace exposure accounted for 3.8% (9 cases), and community exposure accounted for 2.5% (6 cases).

### Viral culture

Three studies (Ladhani 2020a, Speake 2020, Yang 2020) performed viral culture ( Table 8). All studies utilised Vero E6 cells for viral culture. In Ladhani 2020a (a study of elderly nursing home residents), positive samples with a Ct of <35 were incubated on Vero E6 cells and confirmed by cytopathic effect (CPE) up to 14 days post-inoculation. Positive culture results were obtained for symptomatic, post-symptomatic, pre-symptomatic and asymptomatic cases (21 residents and 12 staff); higher Ct values was significantly associated with decreasing ability to recover the virus (p<0.001). Among residents the virus was isolated 12 days before symptom onset and up to 13 days after and in staff up to 6 days before and 7 days after symptom onset. In Speake 2020, specimens were inoculated in Vero-E6 cells and inspected for CPE daily for up to 10 days with identity confirmed using “in-house” PCRs. The primary cases had boarded the flight from a cruise ship and had SARS-CoV-2 with the strain A2-Ruby Princess (A2-RP). Nine of 17 (53%) of PCR-positive samples grew SARS-CoV-2 in culture. Eight secondary cases who were in the same flight cabin with the infected travellers from the cruise ship all had viruses of the A2-RP strain (3 by full and 1 by partial sequence) ( Table 8). In the third study of index patients with recurrent infection swab specimens were also inoculated on Vero cells, and monitored for CPE daily for 10 days (Yang 2020). All four viral cultures were negative (0%).

**Table 8.** Results of viral cultures.

Study ID	Types of participants	Method used for viral culture	Results of viral culture
Ladhani 2020a	Staff and residents of 6 London care homes	All SARS-CoV-2 positive samples with a Ct value of >35 were incubated on Vero E6 mammalian cells and virus detection was confirmed by cytopathic effect (CPE) up to 14 days post-inoculation	87 samples with Ct values <35 were cultured and infectious virus was recovered from all (21 residents and 12 staff). Live virus was isolated up to 13 days after and 12 days before symptom onset among residents and up to 6 days before and 7 days after symptom onset among staff. Higher Ct values was significantly associated with decreasing ability to recover infectious virus (p<0.001). There were no significant differences in virus recovery rates between symptomatic and asymptomatic residents (5/17 [29.4%] vs. 14/33 [42.4%]; P = 0.37) and staff (2/6 [33.3%] vs. 10/31 [32.3%]; P = 0.96) at the time of testing.
Speake 2020	241 airline passengers some of whom had disembarked from 1 of 3 cruise ships that had recently docked in Sydney Harbour. 6 primary cases initially	Virus culture was attempted for primary samples . Clinical specimens were inoculated in triplicate wells with Vero-E6 cells at 80% confluency, incubated at 37°C in 5% CO2, and inspected for cytopathic effect daily for up to 10 days. Identity was confirmed by in-house PCRs as described for previous sequences.	9/17 of PCR positive samples grew SARS-CoV-2 on viral culture. Sufficient viral RNA was available to generate an adequate sequence for 25 of the 29 samples positive by PCR. 11 passengers had PCR-confirmed SARS-CoV-2 infection and symptom onset within 48 hours of the flight. All 11 passengers had been in the same cabin with symptomatic persons who had culture- positive A2-RP virus strain.
Yang 2020	Home quarantine: 93 recurrent- positive patients; 96 close contacts and 1,200 candidate contacts	Vero-E6 cells were used for virus isolation in a BSL- 3 laboratory.	Viral culture of 4 specimens with Ct <30 were negative

### Genome sequencing (GS) and phylogenetic analysis

Ten studies (Böhmer 2020, Firestone 2020, Jiang 2020, Ladhani 2020a, Lucey 2020, Pung 2020, Sikkema 2020, Speake 2020, Taylor 2020, Wang 2020) performed GS and phylogenetic analysis ( Table 9). The studies were primarily conducted in outbreak clusters and methods used for performing these investigations were essentially similar across the studies. The completeness of genomic similarity ranged from 81–100% across six studies (Firestone 2020, Jiang 2020, Lucey 2020, Sikkema 2020, Speake 2020, Wang 2020). Transmission from one case to a contact was demonstrated by nonsynonymous nucleotide polymorphism in SARS-CoV-2 from these two cases onwards, but not in any cases detected prior to this instance (Böhmer 2020). In one study of skilled nursing home facilities (Taylor 2020), samples from 75 residents and five healthcare staff shared genetically related strains. In another study of care homes (Ladhani 2020a), reported nine separate introductions of SARS-CoV-2 into care homes by healthcare staff. In one study which used multiple settings (Pung 2020), the viral genomic sequences for four cases in one cluster shared identical sequences over the full genome length and shared a common base difference relative to the earlier sequences (see Table 8).

**Table 9.** GS and phylogenetic analysis.

Study ID	Study Setting	Method used for WGS	Phylogenetic analysis	Results
Böhmer 2020	Home, workplace	Whole genome sequencing involved Roche KAPA HyperPlus library preparation and sequencing on Illumina NextSeq and MiSeq instruments as well as RT-PCR product sequencing on Oxford Nanopore MinION using the primers described in Corman and colleagues. Patient 1 was sequenced on all three platforms; patients 2–7 were sequenced on Illumina NextSeq, both with and without RT-PCR product sequencing with primers as in Corman and colleagues; and patients 8–11, 14, and 16 were sequenced on Oxford Nanopore MinION. Sequencing of patient 15 was not successful. Sequence gaps were filled by Sanger sequencing.	Not reported	Presymptomatic transmission from patient 4 to patient 5 was strongly supported by virus sequence analysis: a nonsynonymous nucleotide polymorphism (a G6446A substitution) was found in the virus from patients 4 and 5 onwards but not in any cases detected before this point (patients 1–3). Later cases with available specimens, all containing this same substitution, were all traced back to patient 5. The possibility that patient 4 could have been infected by patient 5 was excluded by detailed sequence analysis: patient 4 had the novel G6446A virus detected in a throat swab and the original 6446G virus detected in her sputum, whereas patient 5 had a homogeneous virus population containing the novel G6446A substitution in the throat swab.
Firestone 2020	Motorcycle rally	WGS was conducted at the MDH Public Health Laboratory on 38 specimens using previously described methods.	Phylogenetic relationships, including distinct clustering of viral whole genome sequences, were inferred based on nucleotide differences via IQ-TREE using general time reversible substitution models as a part of the Nextstrain workflow.	38 (73%) specimens (23 [61%] from primary and 15 [39%] from secondary and tertiary cases) were successfully sequenced, covering at least 98% of the SARS-CoV-2 genome. Six genetically similar clusters with known epidemiologic links were identified (i.e., cases in patients who were close contacts or who had common exposures at the rally), five of which demonstrated secondary or secondary and tertiary transmission.
Jiang 2020	Home	Positive samples were sequenced directly from the original specimens as previously described. *Reference virus genomes were obtained from GenBank using Blastn with 2019- nCoV as a query. The open reading frames of the verified genome sequences were predicted using Geneious (version 11.1.5) and annotated using the Conserved Domain Database. Pairwise sequence identities were also calculated using Geneious. Potential genetic recombination was investigated using SimPlot software and phylogenetic analysis.	The maximum likelihood phylogenetic tree of the complete genomes was conducted by using RAxML software with 1000 bootstrap replicates, employing the general time-reversible nucleotide substitution model.	The full genome of 8 patients were >99.9% identical across the whole genome. Phylogenetic analysis showed that viruses from patients were clustered in the same clade and genetically similar to other SARS-CoV-2 sequences reported in other countries.
Ladhani 2020a	Care homes	Whole genome sequencing (WGS) was performed on all RT-PCR positive samples. Viral amplicons were sequenced using Illumina library preparation kits (Nextera) and sequenced on Illumina short-read sequencing machines. Raw sequence data was trimmed and aligned against a SARS- CoV-2 reference genome (NC_045512.2). A consensus sequence representing each genome base was derived from the reference alignment.	Consensus sequences were assessed for quality, aligned using MAFFT (Multiple Alignment using Fast Fourier Transform, version 7.310), manually curated and maximum likelihood phylogenetic trees derived using IQtree (version 2.04).	All 158 PCR positive samples underwent WGS analysis and 99 (68 residents, 31 staff) distributed across all the care homes yielded sequence sufficient for WGS analysis. Phylogenetic analysis identified informal clusters, with evidence for multiple introductions of the virus into care home settings. All care home clusters of SARS-CoV-2 genomes included at least one staff member, apart from care home B with no PCR positive staff and high rates of staff self-isolation. Care home A exhibited three distinct sequence clusters and six singletons, potentially representing up to nine separate introductions. Genomic analysis did not identify any differences between asymptomatic/symptomatic residents/staff. The 10 sequences from residents who died were distributed across the lineages identified and were closely matched to sequences derived from non-fatal cases in the same care homes.
Lucey 2020	Hospital	Complementary DNA was obtained from isolated RNA through reverse transcription and multiplex PCR according to the protocol provided by the Artic Network initiative. Libraries were prepared using the NEBNext Ultra II kit (New England Biolabs) and sequenced on an Illumina MiSeq using 300-cycle v2 reagent kits (Illumina). Bowtie 2 was used for aligning the sequencing reads to the reference genome for SARS-CoV-2 (GenBank number, MN908947.3) and SAMtools for manipulating the alignments.	SNPs were used to define clusters and a median-joining network was generated including these data from this study and an additional 1,000 strains collected from GISAID available on May 22nd. Clade annotation was included for the Pangolin, GISAID and NextStrain systems.	WvGS identified six clusters of nosocomial SARS-CoV-2 transmission. The average sequence quality per samples was > 99% for 46 samples, and between 92 and 94% for 4 samples. Phylogenetic analysis identified six independent groups of which clusters 1–3 were related to 39 patients.
Pung 2020	Multiple: Company conference, church, tour group.	Strain names, GISAID EpiCoV accession numbers used for genomic sequencing	Phylogenetic tree utilised the Neighbor-Joining method and confirmed using Maximum Likelihood approaches. Replicate trees with bootstrap used. All ambiguous positions were removed for each sequence pair (pairwise deletion option). Evolutionary analyses were conducted in MEGA X. Strain names, GISAID EpiCoV accession numbers and collection dates are shown, followed by the case number if available.	Cluster A: Viral genomic sequences were available for four cases (AH1, AH2, AH3, and AT1) and phylogenetic analysis confirmed their linkage, as suggested by the epidemiological data.
Sikkema 2020	Hospital	Samples were selected based on a Ct <32. A SARS-CoV-2-specific multiplex PCR for nanopore sequencing was done. The resulting raw sequence data were demultiplexed using qcat. Primers were trimmed using cutadapt,17 after which a reference-based alignment to the GISAID (Global Initiative on Sharing All Influenza Data) sequence EPI_ISL_412973 was done using minimap2. The consensus genome was extracted and positions with a coverage less than 30 reads were replaced with N using a custom script using biopython software (version 1.74) and the python module pysam (version 0.15.3). Mutations in the genome were confirmed by manually checking the alignment, and homopolymeric regions were manually checked and resolved, consulting the reference genome. Genomes were included when having greater than 90% genome coverage. All available full-length SARS-CoV-2 genomes were retrieved from GISAID20 on March 20, 2020 (appendix 1 pp 8–65), and aligned with the newly obtained SARS-CoV-2 sequences in this study using the multiple sequence alignment software MUSCLE (version 3.8.1551). Sequences with more than 10% of N position replacements were excluded. The alignment was manually checked for discrepancies, after which the phylogenomic software IQ-TREE (version 1.6.8) was used to do a maximum- likelihood phylogenetic analysis, with the generalised time reversible substitution model GTR+F+I+G4 as best predicted model. The ultrafast bootstrap option was used with 1000 replicates. Clusters were ascertained based on visual clustering and lineage designations.	The code to generate the minimum spanning phylogenetic tree was written in the R programming language. Ape24 and igraph software packages were used to write the code to generate the minimum spanning tree, and the visNetwork software package was used to generate the visualisation. Pairwise sequence distance (used to generate the network) was calculated by adding up the absolute nucleotide distance and indel- block distance. Unambiguous positions were dealt with in a pairwise manner. Sequences that were mistakenly identified as identical, because of transient connections with sequences containing missing data, were resolved.	46 (92%) of 50 sequences from health-care workers in the study were grouped in three clusters. Ten (100%) of 10 sequences from patients in the study grouped into the same three clusters:
Speake 2020	Aircraft	Processed reads were mapped to the SARS-CoV-2 reference genome (GenBank accession no. MN908947). Primer- clipped alignment files were imported into Geneious Prime version 2020.1.1 for coverage analysis before consensus calling, and consensus sequences were generated by using iVar version 1.2.2.	Genome sequences of SARS- CoV-2 from Western Australia were assigned to lineages by using the Phylogenetic Assignment of Named Global Outbreak LINeages (PANGOLIN) tool ( https://github.com/cov-lineages/pangolinExternal Link). On July 17, 2020, we retrieved SARS-CoV-2 complete genomes with corresponding metadata from the GISAID database. The final dataset contained 540 GISAID whole-genome sequences that were aligned with the sequences from Western Australia generated in this study by using MAFFT version 7.467. Phylogenetic trees were visualized in iTOL (Interactive Tree Of Life, https:// itol.embl.deExternal Link) and MEGA version 7.014.	100% coverage was obtained for 21 and partial coverage (81%–99%) for 4 samples. The phylogenetic tree for the 21 complete genomes belonged to either the A.2 (n = 17) or B.1 (n = 4) sublineages of SARS-CoV-2
Taylor 2020	Skilled nursing facilities	WGS was conducted by MDH-PHL on available specimens using previously described methods.	Phylogenetic relationships, including distinct clustering of viral whole genome sequences, were inferred based on nucleotide differences via IQ-TREE, using general time reversible substitution models	Specimens from 18 (35%) residents and seven (18%) HCP at facility A were sequenced - Strains from 17 residents and five HCP were genetically similar. At facility B, 75 (66%) resident specimens and five (7%) HCP specimens were sequenced, all of which were genetically similar.
Wang 2020	Home	Full genomes were sequenced using the BioelectronSeq 4000. WGS integrated information from 60 published genomic sequences of SARS-CoV-2. Full-length genomes were combined with published SARS-CoV-2 genomes and other coronaviruses and aligned using the FFT- NS-2 model by MAFFT.	Maximum-likelihood phylogenies were inferred under a generalised-time-reversal (GTR)+ gamma substitution model and bootstrapped 1000 times to assess confidence using RAxML.	The phylogenetic tree of full-length genomes showed that SARS-CoV-2 strains form a monophyletic clade with a bootstrap support of 100%. Sequences from six HCWs in the Department of Neurosurgery and one family member were closely related in the phylogenetic tree. 33 family members of the HCWs were not secondarily infected, due to the strict self-quarantine strategies taken by the HCWs immediately after their onset of illness, including wearing a facial mask when they came home, living alone in a separated room, never eating together with their families.

## Discussion

### Summary of main findings

We identified 171 primary studies assessing the role of close contact in transmission of SARS-CoV-2. The evidence from these observational studies suggest that the risk of transmission is significantly increased through close contact with an infected case - the greater the frequency of contact, the greater the risk. Household contact setting is significantly more likely to result in transmission of SARS-CoV-2 compared to other types of contact settings. This risk of transmission appears to decrease with use of face masks and in cases where the index or primary cases are in the paediatric age group. The risk of close contact transmission is significantly increased in the elderly. Enclosed environments and social gatherings appear to increase the likelihood of close contact transmission. Close contact with persons having recurrent infection with SARS-CoV-2 is unlikely to result in transmission of the virus. There is wide heterogeneity in study designs and methods and the overall quality of evidence from published primary studies is sub-optimal. The results of systematic reviews also suggest that household contact setting increases the risk of transmission and being elderly is also associated with increased risks of transmission and mortality.

The positive results of viral cultures observed in two studies support the results of PCR and serologic tests showing that close contact setting was associated with transmission of SARS-CoV-2. The failure to successfully isolate the virus in the third study supports the view that individuals who are re-infected are unlikely to transmit the virus in close contact settings. The positive findings from all 10 studies that performed GS and phylogenetic analysis with identical strains supports the hypothesis that close contact setting is associated with SARS-CoV-2 transmission through respiratory droplets or direct contact. The failure of the majority of studies to report Ct values casts doubts on the strengths of any reported associations because of the likelihood of false positives, as is the lack of (and variation in) reporting of the timelines for sample collections. The variations observed in the definitions of close contacts also cast further doubts on the validity of overall results.

### Comparison with the existing literature

The results of our review are consistent with several guidelines suggesting that close contact with index cases can result in transmission of SARS-CoV-2 ^8– 10^. Our findings are also consistent with those of a systematic review which concluded that face masks are effective for preventing transmission of respiratory viruses ^11^. The results of our review also support those of a previous review which showed that the elderly are at increased risk of infection and mortality with coronavirus ^12^. However, our review contains a greater number of studies compared to each of the included individual reviews and shows evidence demonstrating positive culture of virus as well as genomic evidence of close contact transmission. This differs from the findings from our reviews of fomite, orofecal and airborne transmission that failed to show evidence of either positive culture or genomic sequences demonstrating SARS-CoV-2 transmission ^13– 15^. 

### Strengths and limitations

To our knowledge, this is the most comprehensive review to date investigating the role of close contact in the transmission of SARS-CoV-2. We extensively searched the literature for eligible studies, accounted for the quality of included studies and have reported outcomes (viral culture and GS) that were previously unreported in previous reviews. However, we recognize some limitations. We may not have identified all relevant studies examining the role of close contact in transmission - this is especially true for unpublished studies. We included results from non-peer reviewed studies which may affect the reliability of the review results. However, such studies could potentially be of research benefit because of the ongoing pandemic; in addition, we performed forward citation search of relevant studies. 

### Implications for research

Future studies should endeavour to include Ct values (or preferably convert the Ct values to number of genome copies using standard curves) when reporting research results and should describe the timing and methods of sample collection. Details surrounding the proximity, timing, and activities within the context of close contact need to be described. In studies of elderly subjects, more detailed description of baseline demographics should be reported. Further studies showing virus isolation in close contact settings should be conducted to strengthen the current evidence base; this could include performing serial cultures. Similarly, more research examining genomic sequences and phylogenetic trees in suspected close contact transmissions should be conducted - this should also extend to research examining other modes of transmission. The variation in methods and thresholds of the serological tests add to the confusion about diagnostic accuracy of testing; indeed, some authors have questioned the value of serological tests for diagnosing SARS-CoV-2 ^16^. To overcome the challenge of interpreting antibody responses, guidelines for better reporting of serological tests and results should be developed; this has previously been emphasized by other authors. Internationally recognized research dictionary of terms defining and describing close contact settings should be developed. Standardized guidelines for reporting research results should be a priority. Local, national, and international health organisations should promote good hygiene measures including advising against close contact with SARS-CoV-2 infected individuals; use of medical masks should be encouraged in circumstances where close contact with infected cases is likely. Activities in enclosed settings should be discouraged and social distancing in close contact settings should be encouraged. 

## Conclusion

The evidence from published observational studies and systematic reviews indicate that SARS-CoV-2 can be transmitted via close contact settings. Household contact and increased frequency of contact with infected cases significantly increase the risk of transmission. The quality of evidence from published studies is low-to moderate. Variations in study designs and methodology restrict the comparability of findings across studies. Standardized guidelines for the reporting of future research should be developed.

## Data availability

### Underlying data

All data underlying the results are available as part of the article and no additional source data are required.

### Extended data

Figshare: Extended data: SARS-CoV-2 and the Role of Close Contact in Transmission: A Systematic Review, https://doi.org/10.6084/m9.figshare.14312630.v1 ^6^.

This project contains the following extended data:

- Updated Protocol
- Search Strategy
- List of Excluded Studies
- References to Included Studies

Data are available under the terms of the Creative Commons Attribution 4.0 International license (CC-BY 4.0).

## References

[1] World Health Organization: WHO Coronavirus Disease (COVID-19) Dashboard. [Last Accessed 20/03/2021]. Reference Source

[2] World Health Organization: Modes of transmission of virus causing COVID-19: implications for IPC precaution recommendations. [Accessed 16/01/2021]. Reference Source

[3] World Health Organization: Coronavirus disease 2019 (COVID-19) Situation Report – 73. [Accessed 28 February 2021]. Reference Source

[4] Tanne JH : Covid-19: CDC publishes then withdraws information on aerosol transmission. *BMJ.* 2020;370:m3739. 10.1136/bmj.m3739 32973037

[5] Tang JW : SARS-CoV-2 and aerosols-Arguing over the evidence. *J Virol Methods.* 2021;289:114033. 10.1016/j.jviromet.2020.114033 33285192 PMC7716743

[6] Onakpoya I Heneghan C Spencer E : Extended data: SARS-CoV-2 and the Role of Close Contact in Transmission: A Systematic Review. *figshare.* Figure.2021. 10.6084/m9.figshare.14312630.v1 PMC9636487 36398277

[7] Whiting PF Rutjes AWS Westwood ME : QUADAS-2: a revised tool for the quality assessment of diagnostic accuracy studies. *Ann Intern Med.* 2011;155(8):529–36. 10.7326/0003-4819-155-8-201110180-00009 22007046

[8] World Health Organization: Q&A: How is COVID-19 transmitted?[Accessed 06/04/2021]. Reference Source

[9] Center for Disease Control and Prevention: COVID-19 Overview and Infection Prevention and Control Priorities in non-US Healthcare Settings.Updated Feb. 26, 2021. Reference Source

[10] Public Health England: Guidance 12. COVID-19 infection prevention and control guidance: glossary of terms.Updated 21 January 2021. Reference Source

[11] Liang M Gao L Cheng C : Efficacy of face mask in preventing respiratory virus transmission: A systematic review and meta-analysis. *Travel Med Infect Dis.* 2020;36:101751. 10.1016/j.tmaid.2020.101751 32473312 PMC7253999

[12] Park JE Jung S Kim A : MERS transmission and risk factors: a systematic review. *BMC Public Health.* 2018;18(1):574. 10.1186/s12889-018-5484-8 29716568 PMC5930778

[13] Onakpoya IJ Heneghan CJ Spencer EA : SARS-CoV-2 and the role of fomite transmission: a systematic review [version 1; peer review: awaiting peer review]. *F1000Res.* 2021;10:233. 10.12688/f1000research.51590.1 34136133 PMC8176266

[14] Heneghan CJ Spencer EA Brassey J : SARS-CoV-2 and the role of orofecal transmission: a systematic review [version 1; peer review: awaiting peer review]. *F1000Res.* 2021;10:231. 10.12688/f1000research.51592.1 35035883 PMC8749895

[15] Heneghan C Spencer EA Brassey J : SARS-CoV-2 and the role of airborne transmission: a systematic review [version 1; peer review: awaiting peer review]. *F1000Res.* 2021;10:232. 10.12688/f1000research.52091.1

[16] Bastos ML Tavaziva G Abidi SK : Diagnostic accuracy of serological tests for covid-19: systematic review and meta-analysis. *BMJ.* 2020;370:m2516. 10.1136/bmj.m2516 32611558 PMC7327913

