# Supplementary material for: SARS-CoV-2 and the role of close contact in transmission: a systematic review
Source: F1000Res. 2022 Nov 17;10:280. Originally published 2021 Apr 9. [Version 3] doi: 10.12688/f1000research.52439.3 (PMC9636487; doi:10.12688/f1000research.52439.3)
Supplement: Peer review history

# Peer review: Reviewer response for version 3

**DOI:** 10.5256/f1000research.140982.r156032

**Published:** 2022-11-18

**Authors:** Tetsuya Akaishi

**Affiliations:** 1 Tohoku University, Sendai, Japan

I confirm that the authors have precisely answered my comments and concerns.

Are the rationale for, and objectives of, the Systematic Review clearly stated?

Partly

Is the statistical analysis and its interpretation appropriate?

Not applicable

Are sufficient details of the methods and analysis provided to allow replication by others?

Partly

Are the conclusions drawn adequately supported by the results presented in the review?

Partly

Reviewer Expertise:

Infectious diseases, neurological disorders

I confirm that I have read this submission and believe that I have an appropriate level of expertise to confirm that it is of an acceptable scientific standard.

# Peer review: Reviewer response for version 2

**DOI:** 10.5256/f1000research.135682.r153259

**Published:** 2022-11-04

**Authors:** Gary Lin

**Affiliations:** 1 One Health Trust, Silver Spring, MD, USA; 2 Johns Hopkins University, Baltimore, MD, USA

**Summary**

 The study attempts to understand the transmission of SARS-CoV-2 in close settings. The study was conducted using a systematic review that examined the role of close contact and assessed the evidence in the current literature. Studies were assessed based on various factors, including the risk of bias (QUADAS-2 criteria), definition of close contact, population characteristics, sampling procedures, and PCR cycle thresholds. Studies up to 4/30/2022 have been updated in the latest version. The studies' quality was examined, including factors such as reporting and biases. Due to the heterogeneity of the study, a metanalysis was not conducted. Study settings include educational settings, hospitals, social and religious gatherings, public transport, care homes, professional sports events, households, and quarantine.

 Notable study outcomes include higher attack rates in household and quarantine settings. Settings with high primary attack rates include weddings, karaoke, prison, and households. Home/community, hospital, and social gatherings (concerts and rehearsals) had high secondary attack rates—lower frequency of attacks include educational settings, such as daycare centers. Face masks decreased transmission risk. Symptomatic had a higher risk of transmission. Children had fewer transmission risks compared to the elderly, who had the most transmission risks. Among all studies reported, the definition of close contacts varied. The risk of bias was prevalent in many studies.

**General Comments**

 I applaud the authors for starting and continuing this systematic review up to April 2022 around the growing body of literature on close contact transmission of SARS-CoV-2. The study, from my perspective, is comprehensive and does an excellent job of assessing study bias using the QUADAS-2 methodology. It is also interesting to see a thorough examination of clinical sampling methods, such as PCR Ct and serological threshold values – relevant to the study's strength. This systematic review was a robust study that addressed previous reviewers’ comments thoroughly and rigorously. However, I have a few clarifications that I would like to see:

- Examining each study’s definition of close contact can be useful in determining practical policy recommendations on transmission prevention. However, in studies that examined the frequency, duration, and/or proximity of contacts, how was the data reported, i.e., observation versus self-reported? The reporting methods could be useful in determining the study's strength. Also, in households, did studies consider the household attempts at quarantining the infected individuals?
- Did the studies consider the timing in which they collected data? Were most of these studies done during large-scale NPIs and other social distancing measures? I believe results could be potentially confounded when community prevalence is low. Additionally, the seasonality of community transmission of SARS-CoV-2 and other meteorological factors (e.g. humidity and temperature) can potentially play a factor in mediating transmission (see Lin *et al*., 2022 ^1^). Hence, data collection timing is an issue – especially during the winter versus summer.
- Since environmental and population factors can be a factor in transmission, are there any trends in terms of countries where the studies were conducted?
- Besides age, what other types of demographic factors were considered in studies? You mentioned population characteristics as an examined factor, but you only mention age as a factor that contributed to transmission.

**Minor Issues**

- Figure 3A: Why is “prison” mentioned twice in the legend?

Are the rationale for, and objectives of, the Systematic Review clearly stated?

Yes

Is the statistical analysis and its interpretation appropriate?

Not applicable

Are sufficient details of the methods and analysis provided to allow replication by others?

Yes

Are the conclusions drawn adequately supported by the results presented in the review?

Yes

Reviewer Expertise:

Computational Epidemiology; Systems Science

I confirm that I have read this submission and believe that I have an appropriate level of expertise to confirm that it is of an acceptable scientific standard.

## References

[1] : Investigating the effects of absolute humidity and movement on COVID-19 seasonality in the United States. *Sci Rep* .2022;12(1) : 10.1038/s41598-022-19898-8 16729 10.1038/s41598-022-19898-8 36202875PMC9537426

# Response

**Published:** 2022-11-09

**Authors:** IGHO ONAKPOYA

**Affiliations:** University of Oxford, UK

**Reviewer's Comment: **The study attempts to understand the transmission of SARS-CoV-2 in close settings. The study was conducted using a systematic review that examined the role of close contact and assessed the evidence in the current literature. Studies were assessed based on various factors, including the risk of bias (QUADAS-2 criteria), definition of close contact, population characteristics, sampling procedures, and PCR cycle thresholds. Studies up to 4/30/2022 have been updated in the latest version. The studies' quality was examined, including factors such as reporting and biases. Due to the heterogeneity of the study, a metanalysis was not conducted. Study settings include educational settings, hospitals, social and religious gatherings, public transport, care homes, professional sports events, households, and quarantine.

 Notable study outcomes include higher attack rates in household and quarantine settings. Settings with high primary attack rates include weddings, karaoke, prison, and households. Home/community, hospital, and social gatherings (concerts and rehearsals) had high secondary attack rates—lower frequency of attacks include educational settings, such as daycare centers. Face masks decreased transmission risk. Symptomatic had a higher risk of transmission. Children had fewer transmission risks compared to the elderly, who had the most transmission risks. Among all studies reported, the definition of close contacts varied. The risk of bias was prevalent in many studies.

**Authors' Response: **Thank you. The heterogeneity across the included studies was a main drawback to our findings. This latter point has been mentioned in the Discussion.

**Reviewer's Comment: General Comments**

 I applaud the authors for starting and continuing this systematic review up to April 2022 around the growing body of literature on close contact transmission of SARS-CoV-2. The study, from my perspective, is comprehensive and does an excellent job of assessing study bias using the QUADAS-2 methodology. It is also interesting to see a thorough examination of clinical sampling methods, such as PCR Ct and serological threshold values – relevant to the study's strength. This systematic review was a robust study that addressed previous reviewers’ comments thoroughly and rigorously. However, I have a few clarifications that I would like to see:

**Authors' Response: **We thank the reviewer for these comments. We have made efforts to address the concerns raised.

**Reviewer's Comment: **

- Examining each study’s definition of close contact can be useful in determining practical policy recommendations on transmission prevention. However, in studies that examined the frequency, duration, and/or proximity of contacts, how was the data reported, i.e., observation versus self-reported? The reporting methods could be useful in determining the study's strength. Also, in households, did studies consider the household attempts at quarantining the infected individuals?
- Did the studies consider the timing in which they collected data? Were most of these studies done during large-scale NPIs and other social distancing measures? I believe results could be potentially confounded when community prevalence is low. Additionally, the seasonality of community transmission of SARS-CoV-2 and other meteorological factors (e.g. humidity and temperature) can potentially play a factor in mediating transmission (see Lin *et al*., 2022 ^1^ ). Hence, data collection timing is an issue – especially during the winter versus summer.
- Since environmental and population factors can be a factor in transmission, are there any trends in terms of countries where the studies were conducted?
- Besides age, what other types of demographic factors were considered in studies? You mentioned population characteristics as an examined factor, but you only mention age as a factor that contributed to transmission.

**Authors' Response:**

- We agree with the reviewer that assessing whether the data was observational or self-reported is useful in determining study quality. However, this domain is not included in the QUADAS-2 criteria that we used to assess quality. In households, there was paucity of data on attempts to quarantine infected individuals.
- Most of the studies were done during large scale implementation of NPI interventions. However, not all the studies employed social distancing measures. Although we reported the dates of the included studies when conducted, we did not examine the effect of seasonality in the transmission dynamics. We note the relevance of seasonality on transmission and have added a caveat on this to the limitations section with references (1,2).
- We did not consider trends in transmission per country. However, we reported overall trends in transmission based on settings. However, we have now reported data on the overall SARs across continents. 
- We reported data from 112 studies that addressed the risk of transmission (Table 7). The factors reported in the studies included age, compromised immunity, number of individuals in households, frequency of contacts with infected cases, etc.

**Reviewer's Comment:**

**Minor Issues**

 Figure 3A: Why is “prison” mentioned twice in the legend?

**Authors' Response: **Thanks. We have corrected the error.

**Added References**

 1. Lin G, Hamilton A, Gatalo O, et al. Investigating the effects of absolute humidity and movement on COVID-19 seasonality in the United States. Sci Rep. 2022 Oct 6;12(1):16729. doi: 10.1038/s41598-022-19898-8. PMID: 36202875; PMCID: PMC9537426

 2. Landier J, Paireau J, Rebaudet S, et al. Cold and dry winter conditions are associated with greater SARS-CoV-2 transmission at regional level in western countries during the first epidemic wave. Sci Rep. 2021 Jun 17;11(1):12756. doi: 10.1038/s41598-021-91798-9. PMID: 34140557; PMCID: PMC8211690

# Peer review: Reviewer response for version 2

**DOI:** 10.5256/f1000research.135682.r152964

**Published:** 2022-10-11

**Authors:** Tetsuya Akaishi

**Affiliations:** 1 Tohoku University, Sendai, Japan

I think that the authors vigorously and extensively screened and reviewed the scientific articles regarding secondary transmission rate after a close contact in miscellaneous places. I agree with the authors and other reviewers about the varied criteria of close contact, different seasons of studies, and different implemented infection prevention measures in the localities, between the evaluated articles.

 I suggest the authors to consider stratifications by some essential baseline factors, such as the following variables:

1. Difference in criteria of close contact.
2. Seasons with different prevalent variant strains.
3. Different geographic regions (i.e., Europe, North America, Asia, Africa, etc).

I also recommend the authors to include the information about the types and combinations of the used terms in the primary step for screening the relevant literatures through the databases.

 I agree with the authors about their conclusion that standardized guidelines for (i) judging the closeness of contact (especially the wearing of face masks) and (ii) reporting the estimated sample viral load with cycle threshold will be needed in future studies. This study delineated this issue by comprehensively evaluating a large amount of reported scientific articles, and may benefit future researches in this field.

Are the rationale for, and objectives of, the Systematic Review clearly stated?

Partly

Is the statistical analysis and its interpretation appropriate?

Not applicable

Are sufficient details of the methods and analysis provided to allow replication by others?

Partly

Are the conclusions drawn adequately supported by the results presented in the review?

Partly

Reviewer Expertise:

Infectious diseases, neurological disorders

I confirm that I have read this submission and believe that I have an appropriate level of expertise to confirm that it is of an acceptable scientific standard, however I have significant reservations, as outlined above.

# Response

**Published:** 2022-11-09

**Authors:** IGHO ONAKPOYA

**Affiliations:** University of Oxford, UK

**Reviewer's Comment:** I think that the authors vigorously and extensively screened and reviewed the scientific articles regarding secondary transmission rate after a close contact in miscellaneous places. I agree with the authors and other reviewers about the varied criteria of close contact, different seasons of studies, and different implemented infection prevention measures in the localities, between the evaluated articles.

**Authors' Response:** We thank the reviewer for the positive comments.

**Reviewer's Comment: **I suggest the authors to consider stratifications by some essential baseline factors, such as the following variables: 

1. Difference in criteria of close contact.
2. Seasons with different prevalent variant strains.
3. Different geographic regions (i.e., Europe, North America, Asia, Africa, etc).

**Authors' Response: **

1. We already reported the data on definitions of close contact. Only 28/258 (11%) studies defined close contact based on distance and 163 studies (63%) did not report definitions of close contact. The paucity of suitable information makes it challenging to present the data in this respect. We have highlighted the need for international guidelines on definitions to be developed.
2. We see the reviewer’s point. However, this was not one of our a priori outcome measures. Answering this question would require a separate research endeavour.
3. We have already reported the number of studies for some continents. We have now reported data on the frequencies of SARs by continent based on available data.

**Reviewer's Comment: **I also recommend the authors to include the information about the types and combinations of the used terms in the primary step for screening the relevant literatures through the databases.

**Authors' Response: **Thank you. We have included statements about the screening of abstracts for inclusion

**Reviewer's Comment: **I agree with the authors about their conclusion that standardized guidelines for (i) judging the closeness of contact (especially the wearing of face masks) and (ii) reporting the estimated sample viral load with cycle threshold will be needed in future studies. This study delineated this issue by comprehensively evaluating a large amount of reported scientific articles, and may benefit future researches in this field.

**Authors' Response: **We thank the reviewer for this comment.

# Peer review: Reviewer response for version 1

**DOI:** 10.5256/f1000research.55716.r123867

**Published:** 2022-04-05

**Authors:** Richard Wamai

**Affiliations:** 1 Integrated Initiative for Global Health, Northeastern University, Boston, MA, USA

I have read this manuscript with keen interest and over several weeks during which COVID-19 has continued to evolve with new studies coming out and policy changes across countries I have been traveling in (Kenya and US). This manuscript deals with the question of availability of evidence for role of close contact in COVID-19 transmission. This question seems obvious now given 2+ years of generalized policy around the world for physical or social distancing. A lay observation would be to say that there is unequivocal evidence that close proximity has a role in the transmission of COVID (after all we have been told to distance). The authors of the present manuscript deal with the question of availability of scientific evidence for this generalized policy.

 Clearly, a lot (if not all) of the NPIs (non-pharmaceutical interventions) have been implemented without evidence whether they work (Halperin DT, Hearst N, Hodgins S, *et al. *(2021) ^1^ - I am a co-author in this study. The current manuscript shows just how murky the state of evidence of these NPIs is. Consider the state of evidence of mask-wearing; just the Bangladeshi study is the only RCT we have, and the evidence that masks work from this trial is not that great (Abaluck J, Kwong LH, Styczynski A, *et al., *2022 ^2^)

 In my view, assessment of the merits of this manuscript should center on two things. The first is whether the authors have identified all the evidence available as published in scientific literature. The second is whether the authors make good-faith representation of the studies they have reviewed. I say “good-faith” because there are questions raised by the existing reviews and comments of this manuscript. Additionally, “good-faith” means to me that the authors have not omitted results from the studies. On these two accounts I write my recommendation. A systematic/scoping review is a unique type of study because authors should be presenting in good faith the information from the studies reviewed.

**1. Have the authors examined all of the available evidence (=published studies) on the question?**

 The authors both say they have and have documented and included the method of their search for the literature reviewed (Appendix 2). The only problem I see with this is that studies reviewed were only those published as of December 20, 2020. That was the first full year of the pandemic. Publications from 2021 are not included. One would presume that it is more likely that more studies on the effect of distancing could have been conducted in 2021. Those studies are not included in this manuscript, and that is a shortcoming. One – and at the minimum necessary – revision that could be made is to add the timeframe of the review in the Title of the manuscript. In my view, it is not necessary for the authors to begin including the studies from 2021 as that would require a full new search and a re-write of large potions of the manuscript.

**2. Have the authors presented truthfully and good-faith the evidence from the studies?**

 This is the central question for this manuscript, and it appears more so the case given the extensive comments by the readers. In my view, all of the criticism is not warranted. Criticism is due where the authors have not presented results from the studies reviewed truthfully and in good-faith. Criticism is also due where gaps in evidence is not acknowledged or are pertaining. I do not think the readers present counter evidence, i.e., show that the authors have summarized results from a specific study or set of studies erroneously. In the same vein I am not convinced that readers demonstrate that the authors have misinterpreted results from the studies they have reviewed; such misinterpretation would be strong grounds for Not Approving this manuscript or calling for its revisions, of course. If the readers are presenting evidence to counter the evidence from the studies presented by the authors from other studies that is acceptable. But that should also be weighed against my point 1 above. For instance, could the initial search have missed such studies raised by the readers?

 Also, I would not engage in some of the debates presented. For instance, I do not think the manuscript should be criticized because *“these authors may lack key expertise in their team”*. I have read through the contributors’ listed expertise. I do not think any special expertise above and beyond what the author team has is needed to conduct a systematic review or truthfully and in good-faith summarize the evidence from the reviewed studies. In addition, I do not think that simply because persons in the author team are members of a decision-making body like the WHO that disqualifies them from objectivity or removes their training in science and scholarship. On the contrary, I think there is great value when decision-makers also participate in science. The obvious problem, of course, is where there are conflicts of interests, or where such persons are compromised. However, we have to take the consideration that in a multi-authored manuscript not one of the authors influences the entire narrative of the manuscript or results presented. Persons whose employment or affiliation positions may – or may be perceived to – compromise any perspectives presented in a manuscript they have co-authored should of course declare the conflicts of interests. Declaration of conflicts of interests is a standard protocol required in journal standards.

 Having said the above, I have a few other observations or questions of my own.

Q1: On table 5, many studies are of no assessed quality, i.e., miss the quality measures specified in this table. What if studies missing all (or even just 2) of these measures are excluded in the analysis? So, if only those applicable as judged in Fig 2 are included.

Q2: On page 46, “Risk of Infection”. Is not ‘risk of infection’ similar to AR (attack rate) in the sense that AR reports risk of infection? In this section too (with Fig 3a) we would expect AR and SAR results to indicate risk of infection. Where, correspondingly, ARs and SARs are higher than risk of infection is high. Thus, risk of infection analysis do not present different results. Is this not how this should be understood?

Q3: “Implications for Research”, page 58. The text, *"Local, national, and international health organisations should promote good hygiene measures including advising against close contact with SARS-CoV-2 infected individuals; use of medical masks should be encouraged in circumstances where close contact with infected cases is likely. Activities in enclosed settings should be discouraged and social distancing in close contact settings should be encouraged."* -  To me, and objectively assessing the timing of the pandemic where we are today (April 1 ^st^ 2022), this text is mostly ‘water under the bridge’ and should thus be updated – if this manuscript is to be indexed. See discussions, e.g., in Halperin DT, Hearst N, Hodgins S, *et al. *(2021) ^1^.

 Additionally, I am not convinced the authors have presented compelling evidence here to warrant prompting such measures this text I highlight here include. The authors should strictly adhere to the evidence of their manuscript; it is wrong for the authors to echo the chorus of songs we have heard about these NPIs when there is no or limited evidence they work. Thirdly, as a further reason to exclude this concluding text, in this manuscript there is no discussion about hygiene. Evidence presented does not show close contact be avoided whatever the case. For instance, relevant studies reviewed show no transmission from (index) persons with previous repeated infection. Given this and the fact that by now many (if not most) people will have been infected (i.e., generalized spread) even multiple times (e.g., CDC. Nov. 16, 2021. Estimated COVID-19 Burden, https://www.cdc.gov/coronavirus/2019-ncov/cases-updates/burden.html), should we still be advocating for quarantine and masking? We argue for adherence to evidence (Halperin DT, Hearst N, Hodgins S, *et al*., 2021 ^1^.

 Finally, there will be a few grammatical errors that should be checked.

 I am grateful for the opportunity to read this manuscript and share my observations which I hope will help guide the authors in determining merit worthiness for indexing as well as contribute to continued discussions.

Are the rationale for, and objectives of, the Systematic Review clearly stated?

Yes

Is the statistical analysis and its interpretation appropriate?

Not applicable

Are sufficient details of the methods and analysis provided to allow replication by others?

Yes

Are the conclusions drawn adequately supported by the results presented in the review?

Yes

Reviewer Expertise:

Global public health

I confirm that I have read this submission and believe that I have an appropriate level of expertise to confirm that it is of an acceptable scientific standard, however I have significant reservations, as outlined above.

## References

[1] : Revisiting COVID-19 policies: 10 evidence-based recommendations for where to go from here. *BMC Public Health* .2021;21(1) : 10.1186/s12889-021-12082-z 10.1186/s12889-021-12082-z 34774012PMC8590121

[2] : Impact of community masking on COVID-19: A cluster-randomized trial in Bangladesh. *Science* .2022;375(6577) : 10.1126/science.abi9069 eabi9069 10.1126/science.abi9069 34855513PMC9036942

# Response

**Published:** 2022-06-29

**Authors:** IGHO ONAKPOYA

**Affiliations:** University of Oxford, UK

**Peer reviewer's comment:** I have read this manuscript with keen interest and over several weeks during which COVID-19 has continued to evolve with new studies coming out and policy changes across countries I have been traveling in (Kenya and US). This manuscript deals with the question of availability of evidence for role of close contact in COVID-19 transmission. This question seems obvious now given 2+ years of generalized policy around the world for physical or social distancing. A lay observation would be to say that there is unequivocal evidence that close proximity has a role in the transmission of COVID (after all we have been told to distance). The authors of the present manuscript deal with the question of availability of scientific evidence for this generalized policy.

**Authors' response: **Thank you.

**Peer reviewer's comment:** Clearly, a lot (if not all) of the NPIs (non-pharmaceutical interventions) have been implemented without evidence whether they work (Halperin DT, Hearst N, Hodgins S, *et al. *(2021) 1 - I am a co-author in this study. The current manuscript shows just how murky the state of evidence of these NPIs is. Consider the state of evidence of mask-wearing; just the Bangladeshi study is the only RCT we have, and the evidence that masks work from this trial is not that great (Abaluck J, Kwong LH, Styczynski A, *et al., *2022 2)

**Authors' response: **Thank you for this observation. As at the time we conducted the initial review, the evidence base was poor.

**Peer reviewer's comment:** In my view, assessment of the merits of this manuscript should center on two things. The first is whether the authors have identified all the evidence available as published in scientific literature. The second is whether the authors make good-faith representation of the studies they have reviewed. I say “good-faith” because there are questions raised by the existing reviews and comments of this manuscript. Additionally, “good-faith” means to me that the authors have not omitted results from the studies. On these two accounts I write my recommendation. A systematic/scoping review is a unique type of study because authors should be presenting in good faith the information from the studies reviewed.

**Authors' response: **Thank you for focusing your peer review on the evidence as presented in the manuscript.

**Peer reviewer's comment: **1. Have the authors examined all of the available evidence (=published studies) on the question?

 The authors both say they have and have documented and included the method of their search for the literature reviewed (Appendix 2). The only problem I see with this is that studies reviewed were only those published as of December 20, 2020. That was the first full year of the pandemic. Publications from 2021 are not included. One would presume that it is more likely that more studies on the effect of distancing could have been conducted in 2021. Those studies are not included in this manuscript, and that is a shortcoming. One – and at the minimum necessary – revision that could be made is to add the timeframe of the review in the Title of the manuscript. In my view, it is not necessary for the authors to begin including the studies from 2021 as that would require a full new search and a re-write of large potions of the manuscript.

**Authors' response: **We have updated the electronic searches up till 30/04/2022 because of the comments from reviewer #1 and re-written several aspects of the manuscript to reflect this update.

**Peer reviewer's comment: **2. Have the authors presented truthfully and good-faith the evidence from the studies?

 This is the central question for this manuscript, and it appears more so the case given the extensive comments by the readers. In my view, all of the criticism is not warranted. Criticism is due where the authors have not presented results from the studies reviewed truthfully and in good-faith. Criticism is also due where gaps in evidence is not acknowledged or are pertaining. I do not think the readers present counter evidence, i.e., show that the authors have summarized results from a specific study or set of studies erroneously. In the same vein I am not convinced that readers demonstrate that the authors have misinterpreted results from the studies they have reviewed; such misinterpretation would be strong grounds for Not Approving this manuscript or calling for its revisions, of course. If the readers are presenting evidence to counter the evidence from the studies presented by the authors from other studies that is acceptable. But that should also be weighed against my point 1 above. For instance, could the initial search have missed such studies raised by the readers?

**Authors' response: **We thank the reviewer for making this point. We agree with you that the criticisms were not justified. We searched, identified, and analyzed the available evidence at that time point.

**Peer reviewer's comment: **Also, I would not engage in some of the debates presented. For instance, I do not think the manuscript should be criticized because *“these authors may lack key expertise in their team”*. I have read through the contributors’ listed expertise. I do not think any special expertise above and beyond what the author team has is needed to conduct a systematic review or truthfully and in good-faith summarize the evidence from the reviewed studies. In addition, I do not think that simply because persons in the author team are members of a decision-making body like the WHO that disqualifies them from objectivity or removes their training in science and scholarship. On the contrary, I think there is great value when decision-makers also participate in science. The obvious problem, of course, is where there are conflicts of interests, or where such persons are compromised. However, we have to take the consideration that in a multi-authored manuscript not one of the authors influences the entire narrative of the manuscript or results presented. Persons whose employment or affiliation positions may – or may be perceived to – compromise any perspectives presented in a manuscript they have co-authored should of course declare the conflicts of interests. Declaration of conflicts of interests is a standard protocol required in journal standards.

**Authors' response: **Again, we agree with the reviewer. We have enough expertise in our team and have co-authored hundreds of systematic reviews.

**Peer reviewer's comment: **Having said the above, I have a few other observations or questions of my own.

**Authors' response: **Thank you. We have responded to each observation.

**Peer reviewer's comment:** Q1: On table 5, many studies are of no assessed quality, i.e., miss the quality measures specified in this table. What if studies missing all (or even just 2) of these measures are excluded in the analysis? So, if only those applicable as judged in Fig 2 are included.

**Authors' response: **If we removed studies missing all or even 2 domains, we think the overall quality would still be low to moderate. Only 9 studies adequately dealt with bias.

**Peer reviewer's comment:** Q2: On page 46, “Risk of Infection”. Is not ‘risk of infection’ similar to AR (attack rate) in the sense that AR reports risk of infection? In this section too (with Fig 3a) we would expect AR and SAR results to indicate risk of infection. Where, correspondingly, ARs and SARs are higher than risk of infection is high. Thus, risk of infection analysis do not present different results. Is this not how this should be understood?

**Authors' response: **The AR is a crude measure of the rate of infection in a group. The risk of infection compares the rate of attacks between or across groups, based on other variables, e.g., seating proximity. (See Box 1).

**Peer reviewer's comment:** Q3: “Implications for Research”, page 58. The text, *"Local, national, and international health organisations should promote good hygiene measures including advising against close contact with SARS-CoV-2 infected individuals; use of medical masks should be encouraged in circumstances where close contact with infected cases is likely. Activities in enclosed settings should be discouraged and social distancing in close contact settings should be encouraged."* -  To me, and objectively assessing the timing of the pandemic where we are today (April 1st 2022), this text is mostly ‘water under the bridge’ and should thus be updated – if this manuscript is to be indexed. See discussions, e.g., in Halperin DT, Hearst N, Hodgins S, *et al. *(2021) 1.

**Authors' response: **We understand the point the reviewer makes. These statements were based on our understanding at that time. We have revised this section with the updated evidence. We have included the suggested reference.

**Peer reviewer's comment: **Additionally, I am not convinced the authors have presented compelling evidence here to warrant prompting such measures this text I highlight here include. The authors should strictly adhere to the evidence of their manuscript; it is wrong for the authors to echo the chorus of songs we have heard about these NPIs when there is no or limited evidence they work. Thirdly, as a further reason to exclude this concluding text, in this manuscript there is no discussion about hygiene. Evidence presented does not show close contact be avoided whatever the case. For instance, relevant studies reviewed show no transmission from (index) persons with previous repeated infection. Given this and the fact that by now many (if not most) people will have been infected (i.e., generalized spread) even multiple times (e.g., CDC. Nov. 16, 2021. Estimated COVID-19 Burden, https://www.cdc.gov/coronavirus/2019-ncov/cases-updates/burden.html), should we still be advocating for quarantine and masking? We argue for adherence to evidence (Halperin DT, Hearst N, Hodgins S, *et al*., 2021 1.

**Authors' response: **Thank you for raising this issue and we agree that we should stay focused on the evidence found in our review. We have accordingly revised the text as above.

**Peer reviewer's comment: **Finally, there will be a few grammatical errors that should be checked.

**Authors' response: **Thanks. We have re-checked the manuscript for grammatical errors.

**Peer reviewer's comment: **I am grateful for the opportunity to read this manuscript and share my observations which I hope will help guide the authors in determining merit worthiness for indexing as well as contribute to continued discussions.

**Authors' response: **Thank you for your helpful feedback which we believe has helped to improve the quality of the manuscript.

# Peer review: Reviewer response for version 1

**DOI:** 10.5256/f1000research.55716.r121151

**Published:** 2022-03-07

**Authors:** Kevin Escandón, Angela K. Ulrich

**Affiliations:** 1 Division of Infectious Diseases and International Medicine, University of Minnesota Medical School, Minneapolis, MN, USA; 2 Center for Infectious Disease Research and Policy, University of Minnesota, Minneapolis, MN, USA; 3 Division of Environmental Health Sciences, School of Public Health, University of Minnesota, Minneapolis, MN, USA

**General comments**

 We commend the authors for attempting to conduct a systematic review on one of the most controversial topics related to the COVID-19 pandemic: SARS-CoV-2 transmission. This is an important effort that required much dedication and careful analysis. Unfortunately, we think this manuscript falls short of scientific quality and utility due to major methodologic and conceptual flaws.

 First, this systematic review in its current version fails to provide an accurate and updated picture of the existing evidence. We reviewed this manuscript in February 2022, two years into the pandemic, and while SARS-CoV-2 transmission remains a topic of great relevance, the picture regarding the modes of transmission is much clearer now than one year ago due to numerous epidemiologic and lab-based studies. Given this evidence, the WHO and the general scientific community agree that SARS-CoV-2 can be transmitted via droplet, short-range aerosol, long-range aerosol, and less frequently via fomites. This systematic review should be updated to reflect the most recent evidence. 

 Second, and most importantly, there are methodological flaws, conceptual concerns, and unsupported conclusions, as detailed below. Systematic reviews are designed to summarize evidence on specific questions or focused problems with pre-defined criteria to bring understanding and clarity through insightful analyses (even if no meta-analyses are conducted) of existing evidence. Close contact is not only poorly defined in most articles, but is actually addressed in less than a half of the articles included. Importantly, close contact is not a transmission mechanism itself, rather a feature of transmission mechanism(s). The authors erroneously conclude that studies that identified identical strains in close contacts using genome sequencing and phylogenetic analysis support transmission via respiratory droplets. The authors do not acknowledge that short-range aerosol transmission is also a possible (and a likely one) explanation.

 Note that given the absence of line numbers, we are not providing numbered comments.

**Introduction**

- The introduction should be updated as statistics are over one year old (March 2021).
- The authors mention that the “spread of the virus appears to be slowing”. This statement is not necessarily true considering the recent, recurring, and constantly evolving waves of infection attributed to increasingly transmissible variants of SARS-CoV-2. Furthermore, no citation is provided for this statement.
- "Current evidence from epidemiologic and virologic studies suggest SARS-CoV-2 is primarily transmitted via respiratory droplets and direct and indirect contact". This sentence is not properly supported by current data; the authors rather cited two WHO 2021 resources. The authors must acknowledge airborne transmission – a route of transmission accepted by both WHO and CDC. Note that respiratory transmission of inhalable particles is the dominant mode of transmission, especially short-range. Indirect droplet / contact / fomite transmission is estimated to be minor.
  - Recommended references: Zhang & Duchaine 2020 ^1^ and Leung 2021 ^2^.
- The aim of this study is to assess the evidence from primary studies and existing systematic reviews investigating the role of close contact on SARS-CoV-2 transmission. This is aligned with the manuscript title ("SARS-CoV-2 and the role of close contact in transmission..."), the introduction, the methods, and figure and table titles. However, one major challenge is that the "close contact" framework is neither clearly defined in the literature nor is it often standardized in methods of primary studies. Authors address several aspects of transmission, from settings to distance, populations, testing/lab methods (PCR, serology, viral culture, GS and phylogenetic analysis), attack rates, risk of infection, etc. We agree that all of these are variables that influence or describe transmission. But close contact is only defined in 46.8% of included studies which causes concern over the use of consistently applied inclusion criteria. This systematic review seems to evaluate different aims and ends providing general descriptions of different subtopics related to transmission, not only to close contact. It is not appropriate for this study on the role of close contact to make such inference on the interaction between an infector and an infectee if not explicitly stated in the primary study.

**Terminology**

- The authors seem to be using the CDC criteria but this is not clarified in the box definition and the citation link seems to be inadequate.
- It should be "2 days prior to positive test" instead of "2 days prior to test specimen collection".
- Some of the references are textbooks or web resources not easy to navigate or links are not active. Authors are encouraged to use the most relevant scientific articles on COVID-19 wherever possible.
- Synonyms often used to close contact are close/short range and close proximity. The latter could be more beneficial since the word “contact” is often understood as physical and conspicuous encounters.
- While it's understandable that definitions are needed to assess evidence, it is equally important to mention their limitations from the outset. For example, close contact is arbitrary for purposes of contact tracing—we know that the definition results in missing cases of exposure and infection at longer ranges.

**Methods**

- The inclusion of articles identified via preprint servers is justifiable if the search criteria include very recent dates in an attempt to capture the most recent research. However, this systematic review only includes articles up to December 20th 2020 – if the authors choose to use this date or a recent one, articles on preprint servers should not be included.
- The search strategy is not reproducible and more detail is required. For example, a detailed search strategy for the WHO COVID-19 database, LitCovid, medRxiv, and Google Scholar are not included in the Appendix 2. Nor is it clear exactly which keywords were used in the PubMed search, for example, were other terms used to capture the concept of duration and proximity of exposure?
- It is not clear if authors defined “close contact” for inclusion in their systematic review.

- Additional detail is required to explain how the QUADAS-2 tool was adapted for this study and if or how it was validated for this purpose. https://figshare.com/articles/figure/Extended_data_SARS-CoV-2_and_the_Role_of_Close_Contact_in_Transmission_A_Systematic_Review/14312630/1?file=27243050 
- The QUADAS-2 tool was designed for studies primarily designed as diagnostic accuracy studies and its use is likely to fall short to assess the quality of studies for this systematic review.

**Analyses of primary studies**

- Although authors have included extended data containing the protocol and references to included and excluded studies, this remains outdated (March 2021). Several features are discussed and while some of them could suggest close contact transmission, there is quite a bit of heterogeneity in how these studies of transmission inform the role of close contact.
- While we agree that close-contact transmission is a dominant feature of SARS-CoV-2 transmission through the inhalation of respiratory particles, this systematic review does not help advance the aim mentioned - understanding the role of close contact. The authors could revise this work so it separately addresses features of transmission using standardized or limited definitions for each one. Attribution of such analyses to close contact (i.e., its potential role) should result from the interpretation of such findings altogether rather than from "direct assessment of the impact of close contact in transmission" since most studies do not define or measure close contact systematically.

**Analyses of reviews**

- The authors included 10 systematic reviews allegedly investigating close-contact SARS-CoV-2 transmission. Some concerns make questionable the inclusion of such heterogeneous publications for an analysis that at best describes these almost individually without advancing the understanding of the role of close contact in SARS-CoV-2 transmission, which is the ultimate purpose of the authors' systematic review. It is expected that studies included in such publications are primarily observational, since randomized designs are complex and it remains unclear how research should ideally address gaps in our understanding of transmission. Findings of some of the included reviews (Li, Ludvigsson, Zhu) are not described with regard to close contact, but only to population groups; others are only mentioned with regard to asymptomatic infection or attack rates. We find this analysis in general unhelpful.
- The authors analyze features different from close contact in the sections following "Primary studies". All these analyses are related to primary studies, so this should be reorganized for clarity. Again, there is a detailed description of studies that do not address close contact, e.g., "The result of one study (Rosenberg 2020) showed that the incidence of infection significantly increased with age (p<0.0001), while those from another study (Poletti 2020) showed that being 70 years or older was associated with a significantly increased risk of SARS-CoV-2-related death (p<0.001),"

**Discussion**

- Authors should be careful in interpreting what their systematic review found, for instance that many studies report household transmission suggests that transmission occurs in indoor environments with higher exposure. Exposure results from concentration and time of contact with infectious respiratory particles. It, therefore, depends on a mix of frequency of contacts, range of contact, and infectiousness of index cases. But household transmission may perfectly encompass risk of infection beyond 3 m, 6 m, or any other definition of close contact. We do expect that the risk of infection is greatest with the longest contact, though, but the definition of a close contact remains nebulous and the associated risk could vary depending on the environmental conditions that favor transmission. Because the purpose of this systematic review is to address transmission, the authors must discuss at least generally the interplay of these factors. SARS-CoV-2 transmission is a complex phenomenon that depends on the interaction between viral properties (infectious dose and infectivity correlates), the host and their features (breathing rate, respiratory tract morphology, target tissues, receptor distribution, host barriers, immune responses), and the environment (temperature, humidity, salinity, pH, the medium or materials of the contaminated objects or surfaces, ventilation/airflow, ultraviolet radiation). Authors also should acknowledge that existing evidence suggests that transmission in close-contact settings is likely to be dominated by short-range respiratory inhalation of infectious virions.
- The authors superficially mention the role of face masks in decreasing the risk of transmission from the pediatric population. This is a dangerous misinterpretation of evidence of two things that should be analyzed separately, i.e., 1) the efficacy of respiratory protection and 2) the risk of transmissibility from different populations. Certainly, this review was not designed to assess either of these aspects as a research question. As for respiratory protection, not all masks (or respirators) are expected to provide the same degree of protection. And if not correctly/consistently worn, they may not reduce risk. As for the usually overclaimed risk of children to transmit SARS-CoV-2, many potential confounders easily make this group appear highly contagious but it is unlikely this is due to intrinsic features of that population. Therefore, claims about it should be carefully framed to avoid stigmatization or unfair focalization and perceived efficacy of preventive strategies.
- Discussion unrelated to close contact is seen, e.g., "being elderly is also associated with increased risks of transmission and mortality".
- "The positive results of viral cultures observed in two studies support the results of PCR and serologic tests showing that close contact setting was associated with transmission of SARS-CoV-2".
  - We are unsure how this manuscript supports this conclusion. Similarly, this conclusion is not supported by the data "The positive findings from all 10 studies that performed GS and phylogenetic analysis with identical strains supports the hypothesis that close contact setting is associated with SARS-CoV-2 transmission through respiratory droplets or direct contact."
- We agree with the authors on the high heterogeneity of existing studies and that "The variations observed in the definitions of close contacts also cast further doubts on the validity of overall results."
- "The results of our review are consistent with several guidelines suggesting that close contact with index cases can result in transmission of SARS-CoV-2"
  - The authors acknowledge that the evidence for close contact transmission is only low-to-moderate quality, thus, it is a stretch to say that close contact is consistently demonstrated as a risk factor. Guidelines have been evolving throughout the pandemic and while close contact may be associated with increased risk of transmission, this study could provide much stronger evidence if transmission properties were assessed in a clearly defined, systematic, and reproducible way.
- "Our findings are also consistent with those of a systematic review which concluded that face masks are effective for preventing transmission of respiratory viruses."
  - It is unclear how this systematic review regarding the role of close contract on transmission events contributes to the discussion regarding the role of face masks – more description is needed to make this link explicit. Furthermore, the efficacy of face masks is a complex topic and confounded by a number of factors.

**Necessary updates**

- Several of the preprints included in the systematic review and/or cited in the manuscript have been published; given that this manuscript is outdated and an update is needed, authors should take that opportunity to update preprints that have been published and make sure their findings remain unchanged and adjust accordingly. Some of the preprints now published are:
  Helsingen 2020 ^3^ 
  Paireau 2020 ^4^
  Kuwelker 2020 ^5^ 
  Lyngse 2020 ^6^ 
  Jones 2020 ^7^ 
  Chen 2020 ^8^ 
  Fontanet 2020 ^9^
  Armann 2020 ^10^ 
  Charlotte 2020 ^11^ 
  Angulo-Bazan 2020 ^12^ 

Are the rationale for, and objectives of, the Systematic Review clearly stated?

Partly

Is the statistical analysis and its interpretation appropriate?

Partly

Are sufficient details of the methods and analysis provided to allow replication by others?

No

Are the conclusions drawn adequately supported by the results presented in the review?

No

Reviewer Expertise:

Infectious diseases epidemiology, virology, public health.

We confirm that we have read this submission and believe that we have an appropriate level of expertise to state that we do not consider it to be of an acceptable scientific standard, for reasons outlined above.

## References

[1] : SARS-CoV-2 and Health Care Worker Protection in Low-Risk Settings: a Review of Modes of Transmission and a Novel Airborne Model Involving Inhalable Particles. *Clinical Microbiology Reviews* .2020;34(1) : 10.1128/CMR.00184-20 10.1128/CMR.00184-20 PMC760530933115724

[2] : Transmissibility and transmission of respiratory viruses. *Nat Rev Microbiol* .19(8) : 10.1038/s41579-021-00535-6 528-545 10.1038/s41579-021-00535-6 33753932PMC7982882

[3] : Covid-19 transmission in fitness centers in Norway - a randomized trial. *BMC Public Health* .2021;21(1) : 10.1186/s12889-021-12073-0 10.1186/s12889-021-12073-0 34789188PMC8595959

[4] : Early chains of transmission of COVID-19 in France, January to March 2020. *Euro Surveill* .27(6) : 10.2807/1560-7917.ES.2022.27.6.2001953 10.2807/1560-7917.ES.2022.27.6.2001953 35144724PMC8832523

[5] : Attack rates amongst household members of outpatients with confirmed COVID-19 in Bergen, Norway: A case-ascertained study. *Lancet Reg Health Eur* .2021;3: 10.1016/j.lanepe.2020.100014 100014 10.1016/j.lanepe.2020.100014 33871470PMC8009692

[6] : Nationwide study on SARS-CoV-2 transmission within households from lockdown to reopening, Denmark, 27 February 2020 to 1 August 2020. *Euro Surveill* .27(6) : 10.2807/1560-7917.ES.2022.27.6.2001800 10.2807/1560-7917.ES.2022.27.6.2001800 35144726PMC8832519

[7] : SARS-CoV-2 transmission during rugby league matches: do players become infected after participating with SARS-CoV-2 positive players?. *Br J Sports Med* .2021;55(14) : 10.1136/bjsports-2020-103714 807-813 10.1136/bjsports-2020-103714 33574043

[8] : Serological evidence of human infection with SARS-CoV-2: a systematic review and meta-analysis. *The Lancet Global Health* .2021;9(5) : 10.1016/S2214-109X(21)00026-7 e598-e609 10.1016/S2214-109X(21)00026-7 PMC804959233705690

[9] : SARS-CoV-2 infection in schools in a northern French city: a retrospective serological cohort study in an area of high transmission, France, January to April 2020. *Euro Surveill* .26(15) : 10.2807/1560-7917.ES.2021.26.15.2001695 10.2807/1560-7917.ES.2021.26.15.2001695 33860747PMC8167414

[10] : SARS-CoV-2 transmissions in students and teachers: seroprevalence follow-up study in a German secondary school in November and December 2020. *BMJ Paediatr Open* .2021;5(1) : 10.1136/bmjpo-2021-001036 e001036 10.1136/bmjpo-2021-001036 34192197PMC7992381

[11] : High Rate of SARS-CoV-2 Transmission Due to Choir Practice in France at the Beginning of the COVID-19 Pandemic. *J Voice* .2020; 10.1016/j.jvoice.2020.11.029 10.1016/j.jvoice.2020.11.029 33386191PMC7833901

[12] : Household transmission of SARS-CoV-2 (COVID-19) in Lima, Peru. *Cad Saude Publica* .2021;37(3) : 10.1590/0102-311X00238720 e00238720 10.1590/0102-311X00238720 33729282

# Response

**Published:** 2022-06-29

**Authors:** IGHO ONAKPOYA

**Affiliations:** University of Oxford, UK

**Peer reviewers, comment:** We commend the authors for attempting to conduct a systematic review on one of the most controversial topics related to the COVID-19 pandemic: SARS-CoV-2 transmission. This is an important effort that required much dedication and careful analysis. Unfortunately, we think this manuscript falls short of scientific quality and utility due to major methodologic and conceptual flaws.

**Authors' response:** We thank the reviewers for their positive comments and constructive criticisms. We have extensively revised the manuscript to address their concerns.

**Peer reviewers' comment: **First, this systematic review in its current version fails to provide an accurate and updated picture of the existing evidence. We reviewed this manuscript in February 2022, two years into the pandemic, and while SARS-CoV-2 transmission remains a topic of great relevance, the picture regarding the modes of transmission is much clearer now than one year ago due to numerous epidemiologic and lab-based studies. Given this evidence, the WHO and the general scientific community agree that SARS-CoV-2 can be transmitted via droplet, short-range aerosol, long-range aerosol, and less frequently via fomites. This systematic review should be updated to reflect the most recent evidence.

**Authors' response: **The review was submitted in March last year at the start of the pandemic; however, it took a long time before undergoing peer review. We have now updated the review to reflect the most recent evidence focused on the transmission associated with close contact. We updated our searches up till 30/04/2022.

**Peer reviewers' comment: **Second, and most importantly, there are methodological flaws, conceptual concerns, and unsupported conclusions, as detailed below. Systematic reviews are designed to summarize evidence on specific questions or focused problems with pre-defined criteria to bring understanding and clarity through insightful analyses (even if no meta-analyses are conducted) of existing evidence. Close contact is not only poorly defined in most articles, but is actually addressed in less than a half of the articles included. Importantly, close contact is not a transmission mechanism itself, rather a feature of transmission mechanism(s). The authors erroneously conclude that studies that identified identical strains in close contacts using genome sequencing and phylogenetic analysis support transmission via respiratory droplets. The authors do not acknowledge that short-range aerosol transmission is also a possible (and a likely one) explanation.

**Authors' response: **We used the term “close contact settings” for our review, and we acknowledge variations in the definitions of close contact across the studies included in our review (see Table 3). We do not make any claims that close contact is a transmission mechanism but is associated with transmission from any of a number of mechanisms. We have added (with reference) that short-range aerosol transmission is a possible explanation for the identified identical strains in close contacts.

**Peer reviewers' comment: **Note that given the absence of line numbers, we are not providing numbered comments.

**Authors' response: **OK

**Introduction**

**Peer reviewers' comment: **

- The introduction should be updated as statistics are over one year old (March 2021).

The authors mention that the “spread of the virus appears to be slowing”. This statement is not necessarily true considering the recent, recurring, and constantly evolving waves of infection attributed to increasingly transmissible variants of SARS-CoV-2. Furthermore, no citation is provided for this statement.

**Authors' response: **We have updated the searches to April 2022.

 We have provided a citation to show that the infection rate is decreasing ( https://www.ons.gov.uk/peoplepopulationandcommunity/healthandsocialcare/conditionsanddiseases/articles/coronaviruscovid19/latestinsights), and have also noted that the virus continues to evolve.

**Peer reviewers' comment: **

- "Current evidence from epidemiologic and virologic studies suggest SARS-CoV-2 is primarily transmitted via respiratory droplets and direct and indirect contact". This sentence is not properly supported by current data; the authors rather cited two WHO 2021 resources. The authors must acknowledge airborne transmission – a route of transmission accepted by both WHO and CDC. Note that respiratory transmission of inhalable particles is the dominant mode of transmission, especially short-range. Indirect droplet / contact / fomite transmission is estimated to be minor.
  - Recommended references: Zhang & Duchaine 2020 1 and Leung 2021 2.

**Authors' response: **We wish to thank the reviewer for this comment. We have updated the information and referenced the CDC and the WHO. The CDC statement suggests that exposure with infection occurs in 3 principal ways including inhalation of fine respiratory droplets, deposition of respiratory droplets and particles on exposed mucous membranes, splashes and sprays ‘and touching mucous membranes with hands soiled by virus contained in respiratory fluids” ( Scientific Brief: SARS-CoV-2 Transmission | CDC). They openly acknowledge the relative contributions of the modes of transmission outlined are unquantified and difficult to establish. We have revised the statement to state that the virus is primarily transmitted through exposure to infectious respiratory fluids such as fine aerosols, respiratory droplets, and added a further reference ( https://www.cdc.gov/coronavirus/2019-ncov/science/science-briefs/sars-cov-2-transmission.html). The WHO states “available evidence continues to suggest that SARS-CoV-2 can spread from an infected person’s mouth or nose in small liquid particles when the person coughs, sneezes, sings, breathes or talks, by inhalation or inoculation through the mouth, nose or eyes. These liquid particles are different sizes, ranging from larger ‘respiratory droplets’ to smaller ‘aerosols." Current evidence suggests that the virus spreads mainly between people who are in close contact with each other, typically within 1 metre, They also indicate that “the virus can also spread to others through aerosols at longer (beyond the typical 1 metre distance) distances. The risk of long-distance aerosol transmission is higher in poorly ventilated and/or crowded indoor settings” and further discuss transmission through fomites but acknowledge data is limited. Similar to the CDC they indicate the many challenges in working out the presence and transmission of infectious viruses. Rather than state the respiratory transmission of inhalable particles is the dominant mode of transmission we would prefer a more cautious scientifically based response and acknowledge the gap in knowledge in this area. Infection prevention and control during health care when coronavirus disease (‎COVID-19)‎ is suspected or confirmed (who.int)

**Peer reviewers' comment: **

- The aim of this study is to assess the evidence from primary studies and existing systematic reviews investigating the role of close contact on SARS-CoV-2 transmission. This is aligned with the manuscript title ("SARS-CoV-2 and the role of close contact in transmission..."), the introduction, the methods, and figure and table titles. However, one major challenge is that the "close contact" framework is neither clearly defined in the literature nor is it often standardized in methods of primary studies. Authors address several aspects of transmission, from settings to distance, populations, testing/lab methods (PCR, serology, viral culture, GS and phylogenetic analysis), attack rates, risk of infection, etc. We agree that all of these are variables that influence or describe transmission. But close contact is only defined in 46.8% of included studies which causes concern over the use of consistently applied inclusion criteria. This systematic review seems to evaluate different aims and ends providing general descriptions of different subtopics related to transmission, not only to close contact. It is not appropriate for this study on the role of close contact to make such inference on the interaction between an infector and an infectee if not explicitly stated in the primary study.

**Authors' response: **We thank the reviewer for this comment to which we have thought a great deal. We can respond by stating that although only 46.8% (now 39.1%) defined close contact, the authors in the other included studies reported within their studies that they were investigating close contact. We acknowledge that not all studies provided precise definitions and that is a limitation. We set out to summarize the evidence from published studies that assess the association with close contact transmission in COVID-19.

 As stated earlier, we have used the term “close contact settings” for our review.

**Terminology**

**Peer reviewers' comment: **

- The authors seem to be using the CDC criteria but this is not clarified in the box definition and the citation link seems to be inadequate.

**Authors' response: **Thanks. We have revised the definition.

**Peer reviewers' comment: **It should be "2 days prior to positive test" instead of "2 days prior to test specimen collection"

**Authors' response: **Thanks. We have revised the text accordingly.

**Peer reviewers' comment: **Some of the references are textbooks or web resources not easy to navigate or links are not active. Authors are encouraged to use the most relevant scientific articles on COVID-19 wherever possible

**Authors' response: **We have used the updated citations for the relevant articles.

**Peer reviewers' comment: **Synonyms often used to close contact are close/short range and close proximity. The latter could be more beneficial since the word “contact” is often understood as physical and conspicuous encounters.

**Authors' response: **We have broadly used “close contact setting” to allow us to capture the range of studies assessing transmission characteristics of SARS-CoV-2. We appreciate the reviewers’ suggestions of close proximity/range. However, these may be more useful for more focused review questions as they also have their limitations, e.g., direct contact would not be covered by these; however, direct contact has been described as a subset of close contact in some studies.

**Peer reviewers' comment: **

- While it's understandable that definitions are needed to assess evidence, it is equally important to mention their limitations from the outset. For example, close contact is arbitrary for purposes of contact tracing—we know that the definition results in missing cases of exposure and infection at longer ranges.

**Authors' response: **Thank you for this suggestion. We have noted this in the limitations section.

**Peer reviewers' comment: **

- The inclusion of articles identified via preprint servers is justifiable if the search criteria include very recent dates in an attempt to capture the most recent research. However, this systematic review only includes articles up to December 20th 2020 – if the authors choose to use this date or a recent one, articles on preprint servers should not be included.

**Authors' response: **We have updated the searches and used the most up-to-date citations for the included studies.

**Peer reviewers' comment: **The search strategy is not reproducible and more detail is required. For example, a detailed search strategy for the WHO COVID-19 database, LitCovid, medRxiv, and Google Scholar are not included in the Appendix 2. Nor is it clear exactly which keywords were used in the PubMed search, for example, were other terms used to capture the concept of duration and proximity of exposure?

**Authors' response: **Thank you. We have included the full search strategy.

**Peer reviewers' comment: **

- It is not clear if authors defined “close contact” for inclusion in their systematic review.

**Authors' response: **We used the CDC and WHO definitions (see Box 1).

**Peer reviewers' comment: **Additional detail is required to explain how the QUADAS-2 tool was adapted for this study and if or how it was validated for this purpose. https://figshare.com/articles/figure/Extended_data_SARS-CoV-2_and_the_Role_of_Close_Contact_in_Transmission_A_Systematic_Review/14312630/1?file=27243050

**Authors' response: **We did not use all the domains in QUADAS-2 and have clarified this in the manuscript methods section. We did not validate the checklist, and have noted this in our limitations.

**Peer reviewers' comment: **

- The QUADAS-2 tool was designed for studies primarily designed as diagnostic accuracy studies and its use is likely to fall short to assess the quality of studies for this systematic review.

**Authors' response: **QUADAS-2 is used to assess reporting quality in diagnostic reviews. See https://www.bristol.ac.uk/media-library/sites/quadas/migrated/documents/quadas2reportv4.pdf. As noted in our methods, we adapted the checklist for the review.

**Analyses of primary studies**

**Peer reviewers' comment: **

- Although authors have included extended data containing the protocol and references to included and excluded studies, this remains outdated (March 2021). Several features are discussed and while some of them could suggest close contact transmission, there is quite a bit of heterogeneity in how these studies of transmission inform the role of close contact.

**Authors' response: **We have updated the searches and included the most up-to-date citations.

**Peer reviewers' comment: **

- While we agree that close-contact transmission is a dominant feature of SARS-CoV-2 transmission through the inhalation of respiratory particles, this systematic review does not help advance the aim mentioned - understanding the role of close contact. The authors could revise this work so it separately addresses features of transmission using standardized or limited definitions for each one. Attribution of such analyses to close contact (i.e., its potential role) should result from the interpretation of such findings altogether rather than from "direct assessment of the impact of close contact in transmission" since most studies do not define or measure close contact systematically.

**Authors' response: **We included a table showing how the included studies defined close contact. Our objective was to identify, appraise and summarise the evidence from studies investigating transmission in close contact settings. It is equally possible that with close contact there may be transmission via large respiratory droplets and direct physical contact as well as the possibility of short-range fine aerosol and in all fairness, it should be stated as such and not that inhalation is the dominant route as this reviewer contends. It is best to be consistent with all possibilities as suggested by the CDC and WHO and many other publications.

**Analyses of reviews**

**Peer reviewers' comment: **

- The authors included 10 systematic reviews allegedly investigating close-contact SARS-CoV-2 transmission. Some concerns make questionable the inclusion of such heterogeneous publications for an analysis that at best describes these almost individually without advancing the understanding of the role of close contact in SARS-CoV-2 transmission, which is the ultimate purpose of the authors' systematic review. It is expected that studies included in such publications are primarily observational, since randomized designs are complex and it remains unclear how research should ideally address gaps in our understanding of transmission. Findings of some of the included reviews (Li, Ludvigsson, Zhu) are not described with regard to close contact, but only to population groups; others are only mentioned with regard to asymptomatic infection or attack rates. We find this analysis in general unhelpful.

**Authors' response: **Thanks for this comment. The included systematic reviews are considered appropriate since they do fit the definition of close contact based on our original protocol - this relates to structural and population settings. We can discuss the limitations of these reviews and the included studies. Li, Ludvigsson and Zhu fit the definitions. See https://www.ecdc.europa.eu/en/covid-19/surveillance/surveillance-definitions. Some of the findings from the reviews are helpful. Indeed at least 2 reviews included only studies with high reporting quality.

**Peer reviewers' comment: **

- The authors analyze features different from close contact in the sections following "Primary studies". All these analyses are related to primary studies, so this should be reorganized for clarity. Again, there is a detailed description of studies that do not address close contact, e.g., "The result of one study (Rosenberg 2020) showed that the incidence of infection significantly increased with age (p<0.0001), while those from another study (Poletti 2020) showed that being 70 years or older was associated with a significantly increased risk of SARS-CoV-2-related death (p<0.001),"

**Authors' response: **Thank you for pointing this out and we have revised the section on primary studies. We have enumerated the definitions of close contacts in the included studies in Table 3 and reported that several studies did not report a definition). Several studies showed that being elderly was significantly associated with increased risk of infection. Rosenberg 2020 included household contacts. We have revised the statement.

**Discussion**

**Peer reviewers' comment: **

- Authors should be careful in interpreting what their systematic review found, for instance that many studies report household transmission suggests that transmission occurs in indoor environments with higher exposure. Exposure results from concentration and time of contact with infectious respiratory particles. It, therefore, depends on a mix of frequency of contacts, range of contact, and infectiousness of index cases. But household transmission may perfectly encompass risk of infection beyond 3 m, 6 m, or any other definition of close contact. We do expect that the risk of infection is greatest with the longest contact, though, but the definition of a close contact remains nebulous and the associated risk could vary depending on the environmental conditions that favor transmission. Because the purpose of this systematic review is to address transmission, the authors must discuss at least generally the interplay of these factors. SARS-CoV-2 transmission is a complex phenomenon that depends on the interaction between viral properties (infectious dose and infectivity correlates), the host and their features (breathing rate, respiratory tract morphology, target tissues, receptor distribution, host barriers, immune responses), and the environment (temperature, humidity, salinity, pH, the medium or materials of the contaminated objects or surfaces, ventilation/airflow, ultraviolet radiation). Authors also should acknowledge that existing evidence suggests that transmission in close-contact settings is likely to be dominated by short-range respiratory inhalation of infectious virions.

**Authors' response: **Thank you. We have enumerated the complex range of factors at play and the epidemiologic associations in relation to close contact transmission (with references) and have acknowledged the various potential modes of transmission. We agree that transmission is a complex phenomenon and indeed is poorly understood. Musing about issues surrounding the biology of virus particles is beyond the scope of our review which focuses on epidemiologic associations.

**Peer reviewers' comment: **The authors superficially mention the role of face masks in decreasing the risk of transmission from the pediatric population. This is a dangerous misinterpretation of evidence of two things that should be analyzed separately, i.e., 1) the efficacy of respiratory protection and 2) the risk of transmissibility from different populations. Certainly, this review was not designed to assess either of these aspects as a research question. As for respiratory protection, not all masks (or respirators) are expected to provide the same degree of protection. And if not correctly/consistently worn, they may not reduce risk. As for the usually overclaimed risk of children to transmit SARS-CoV-2, many potential confounders easily make this group appear highly contagious but it is unlikely this is due to intrinsic features of that population. Therefore, claims about it should be carefully framed to avoid stigmatization or unfair focalization and perceived efficacy of preventive strategies.

**Authors' response: **We do not believe we are being superficial but rather trying to stay true to the findings. Our statements regarding the effectiveness of face masks are based on the findings from the included studies and we need to stay focused on the findings and avoid making speculative statements. We have added a caveat that there is uncertainty about the extent to which the different types of masks influence the risk of transmission.

**Peer reviewers' comment: **Discussion unrelated to close contact is seen, e.g., "being elderly is also associated with increased risks of transmission and mortality".

**Authors' response: **Thank you for pointing this out. We have removed the statement.

**Peer reviewers' comment: **

- The positive results of viral cultures observed in two studies support the results of PCR and serologic tests showing that close contact setting was associated with transmission of SARS-CoV-2".
  - We are unsure how this manuscript supports this conclusion. Similarly, this conclusion is not supported by the data "The positive findings from all 10 studies that performed GS and phylogenetic analysis with identical strains supports the hypothesis that close contact setting is associated with SARS-CoV-2 transmission through respiratory droplets or direct contact."

**Authors' response: **We have revised the statement to indicate that transmission can occur in close contact settings.

**Peer reviewers' comment: **We agree with the authors on the high heterogeneity of existing studies and that "The variations observed in the definitions of close contacts also cast further doubts on the validity of overall results."

**Authors' response: **Thank you.

**Peer reviewers' comment: **

- "The results of our review are consistent with several guidelines suggesting that close contact with index cases can result in transmission of SARS-CoV-2"
  - The authors acknowledge that the evidence for close contact transmission is only low-to-moderate quality, thus, it is a stretch to say that close contact is consistently demonstrated as a risk factor. Guidelines have been evolving throughout the pandemic and while close contact may be associated with increased risk of transmission, this study could provide much stronger evidence if transmission properties were assessed in a clearly defined, systematic, and reproducible way.

**Authors' response: **Thank you for this comment. We have revised the sentence to discuss the “association” with transmission as opposed to "can result in transmission”. We have added a caveat to note that guidelines keep evolving based on emerging evidence.

**Peer reviewers' comment: **

- "Our findings are also consistent with those of a systematic review which concluded that face masks are effective for preventing transmission of respiratory viruses."

It is unclear how this systematic review regarding the role of close contract on transmission events contributes to the discussion regarding the role of face masks – more description is needed to make this link explicit. Furthermore, the efficacy of face masks is a complex topic and confounded by a number of factors.

**Authors' response: **We have revised the statements. We have deleted the texts relating to mortality in the elderly.

**Peer reviewers' comment:** **Necessary updates**

- Several of the preprints included in the systematic review and/or cited in the manuscript have been published; given that this manuscript is outdated and an update is needed, authors should take that opportunity to update preprints that have been published and make sure their findings remain unchanged and adjust accordingly. Some of the preprints now published are:
  Helsingen 2020 3
  Paireau 2020 4
  Kuwelker 2020 5
  Lyngse 2020 6
  Jones 2020 7
  Chen 2020 8
  Fontanet 2020 9
  Armann 2020 10
  Charlotte 2020 11
  Angulo-Bazan 2020 12

**Authors' response: **Thank you. We have now updated the citations for the references.
